# Supplementary material for: Lenacapavir-induced capsid damage uncovers HIV-1 genomes emanating from nuclear speckles
Source: EMBO J. 2025 Dec 1;45(2):449–70. doi: 10.1038/s44318-025-00652-5 (PMC12811339; doi:10.1038/s44318-025-00652-5)
Supplement: Supplementary file 2 — Movie EV1 [file 44318_2025_652_MOESM2_ESM.zip › EMBOJ-2025-121832R_MovieEV1_title_legend.docx]

**MovieEV1** - **CLEM-ET analysis of capsid clusters in MDM.**

Reconstructed electron tomogram correlated to the IN.SNAP.SiR signal of the infected MDM shown in Figure1D-G overlayed with a 3D rendering of the tomogram.
