# Supplementary material for: Lenacapavir-induced capsid damage uncovers HIV-1 genomes emanating from nuclear speckles
Source: EMBO J. 2025 Dec 1;45(2):449–70. doi: 10.1038/s44318-025-00652-5 (PMC12811339; doi:10.1038/s44318-025-00652-5)
Supplement: Supplementary file 3 — Movie EV2 [file 44318_2025_652_MOESM3_ESM.zip › EMBOJ-2025-121832R_MovieEV2_title_legend.docx]

**MovieEV2** – **Exposure of previously hidden eGFP.OR3 signals inside the nucleus.**

Live imaging of HeLa-based cells using 3D confocal spinning disc microscopy. New eGFP.OR3 (green) signals appear at positions of IN.SNAP (magenta) objects. Recording starts at 22 h p. i. with a time resolution of 3 minutes per frame. 500 nM LEN was added 6 min after start of imaging (see Figure 3 E,F). Shown is a MIP and scale bar: 2 µm
