# Supplementary material for: Lenacapavir-induced capsid damage uncovers HIV-1 genomes emanating from nuclear speckles
Source: EMBO J. 2025 Dec 1;45(2):449–70. doi: 10.1038/s44318-025-00652-5 (PMC12811339; doi:10.1038/s44318-025-00652-5)
Supplement: Supplementary file 4 — Movie EV3 [file 44318_2025_652_MOESM4_ESM.zip › EMBOJ-2025-121832R_MovieEV3_title_legend.docx]

**MovieEV3 – IN.SNAP objects move to positions of previously present eGFP.OR3 objects.**

Live imaging of HeLa-based cells using 3D confocal spinning disc microscopy. The IN.SNAP (magenta) object moves to the position of the eGFP.OR3 (green) signal, which had been already present prior to the start of imaging. Recording starts at 22 h p. i. with a time resolution of 3 minutes per frame. 500 nM LEN was added 6 min after start of imaging. The white circle indicates the position of the detected eGFP.OR3 signal and the track is shown in blue (see Figure 3G,H). Shown is a MIP and scale bar: 2 µm
