# Supplementary material for: Lenacapavir-induced capsid damage uncovers HIV-1 genomes emanating from nuclear speckles
Source: EMBO J. 2025 Dec 1;45(2):449–70. doi: 10.1038/s44318-025-00652-5 (PMC12811339; doi:10.1038/s44318-025-00652-5)
Supplement: Supplementary file 5 — Movie EV4 [file 44318_2025_652_MOESM5_ESM.zip › EMBOJ-2025-121832R_MovieEV4_title_legend.docx]

**MovieEV4** - **CLEM-ET analysis of LEN-induced capsid alterations.**

Reconstructed electron tomogram correlated to an IN.SNAP.SiR/eGFP.OR3 signal in the nucleus of an infected and LEN treated HeLa-based cell shown in Figure5D overlayed with a 3D rendering of the tomogram. The tomogram shows protrusions at the narrow ends of conical capsid objects and empty/fused lattices.
