# Supplementary figures and images for: Lenacapavir-induced capsid damage uncovers HIV-1 genomes emanating from nuclear speckles (part 1 of 3)
Source: EMBO J. 2025 Dec 1;45(2):449–70. doi: 10.1038/s44318-025-00652-5 (PMC12811339; doi:10.1038/s44318-025-00652-5)

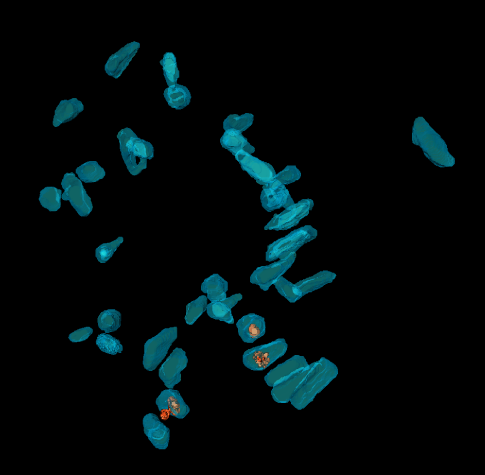

Supplement: Supplementary file 6 — Source data Fig. 1 [file 44318_2025_652_MOESM6_ESM.zip › Figure 1/1F/1F.tif]

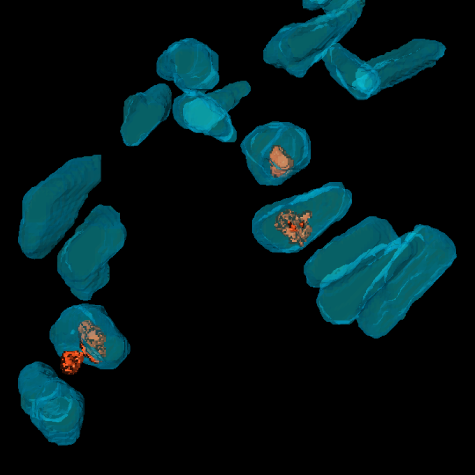

Supplement: Supplementary file 6 — Source data Fig. 1 [file 44318_2025_652_MOESM6_ESM.zip › Figure 1/1G/1G.tif]

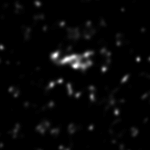

Supplement: Supplementary file 6 — Source data Fig. 1 [file 44318_2025_652_MOESM6_ESM.zip › Figure 1/1B/1B_c1_CA_c2_SRRM2.tif]

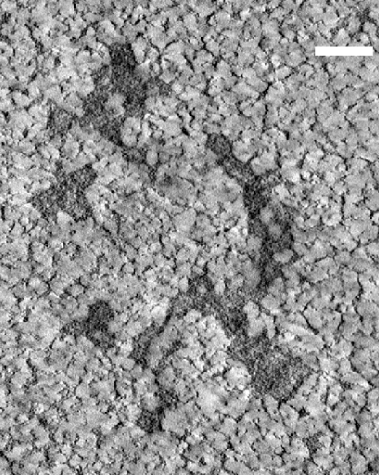

Supplement: Supplementary file 6 — Source data Fig. 1 [file 44318_2025_652_MOESM6_ESM.zip › Figure 1/1E/1E.tif]

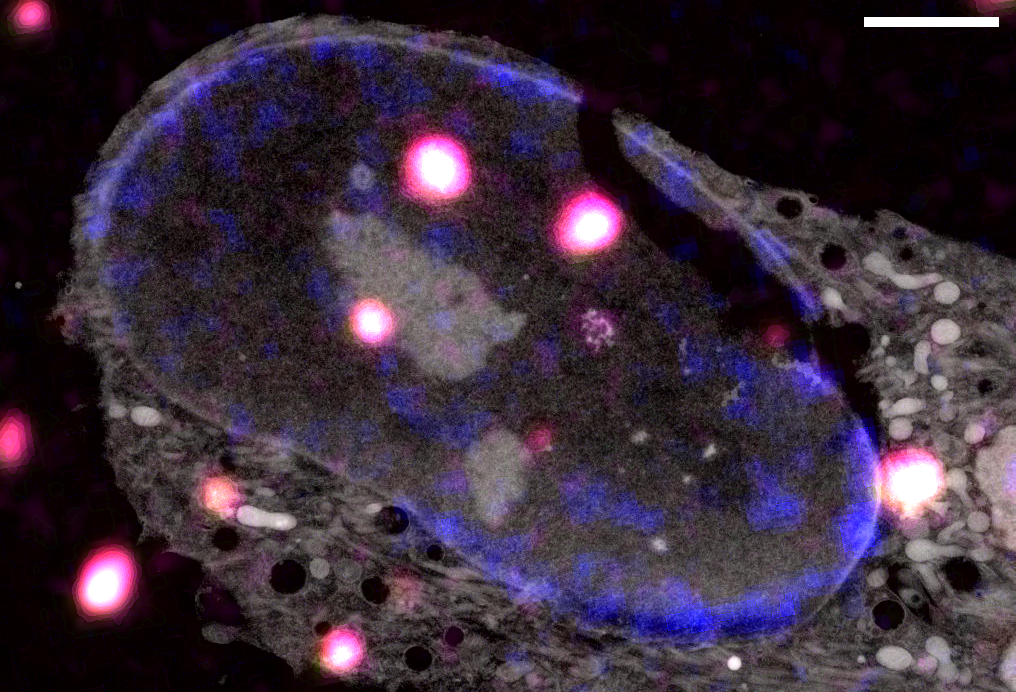

Supplement: Supplementary file 6 — Source data Fig. 1 [file 44318_2025_652_MOESM6_ESM.zip › Figure 1/1D/1D.tif]

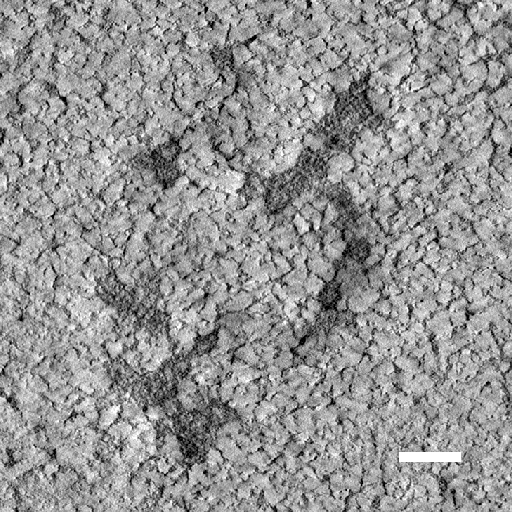

Supplement: Supplementary file 6 — Source data Fig. 1 [file 44318_2025_652_MOESM6_ESM.zip › Figure 1/1E/Fig1E_tiff_stack/modv0013.tif]

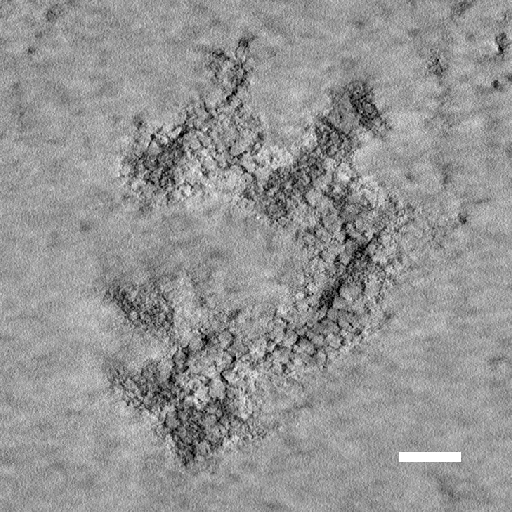

Supplement: Supplementary file 6 — Source data Fig. 1 [file 44318_2025_652_MOESM6_ESM.zip › Figure 1/1E/Fig1E_tiff_stack/modv0007.tif]

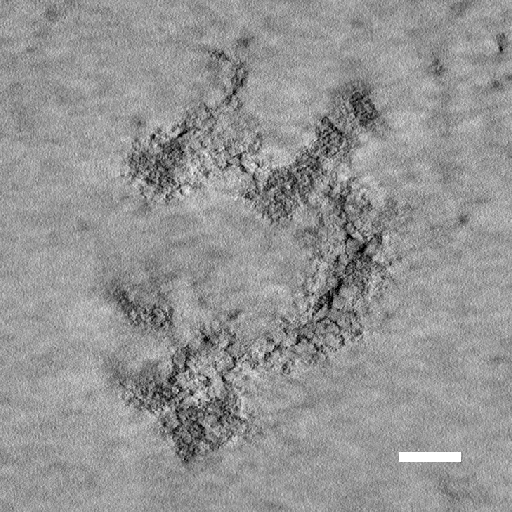

Supplement: Supplementary file 6 — Source data Fig. 1 [file 44318_2025_652_MOESM6_ESM.zip › Figure 1/1E/Fig1E_tiff_stack/modv0006.tif]

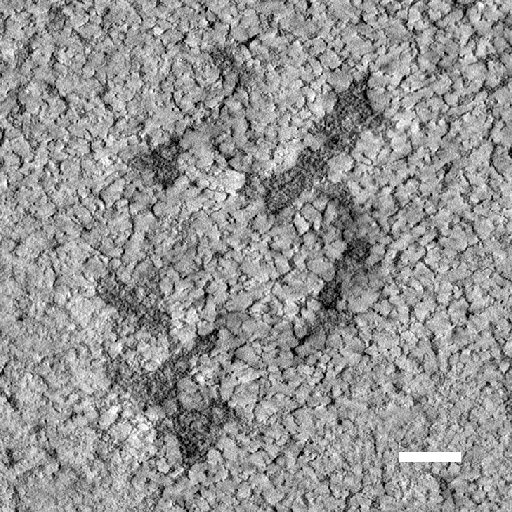

Supplement: Supplementary file 6 — Source data Fig. 1 [file 44318_2025_652_MOESM6_ESM.zip › Figure 1/1E/Fig1E_tiff_stack/modv0012.tif]

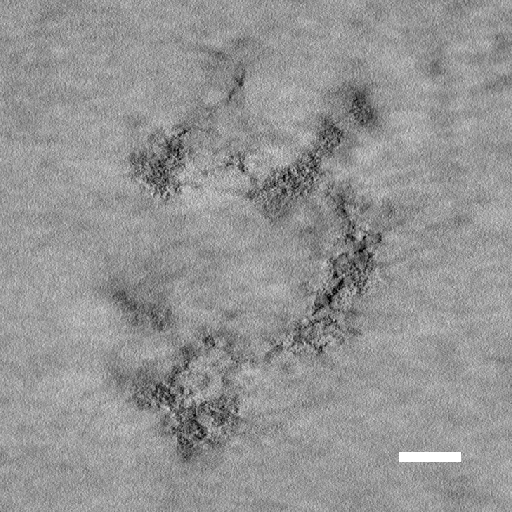

Supplement: Supplementary file 6 — Source data Fig. 1 [file 44318_2025_652_MOESM6_ESM.zip › Figure 1/1E/Fig1E_tiff_stack/modv0004.tif]

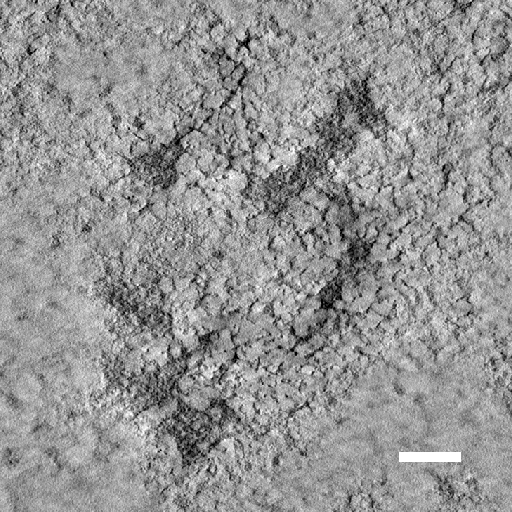

Supplement: Supplementary file 6 — Source data Fig. 1 [file 44318_2025_652_MOESM6_ESM.zip › Figure 1/1E/Fig1E_tiff_stack/modv0010.tif]

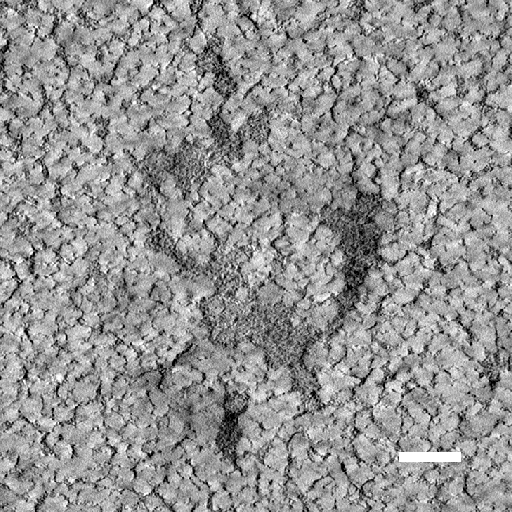

Supplement: Supplementary file 6 — Source data Fig. 1 [file 44318_2025_652_MOESM6_ESM.zip › Figure 1/1E/Fig1E_tiff_stack/modv0038.tif]

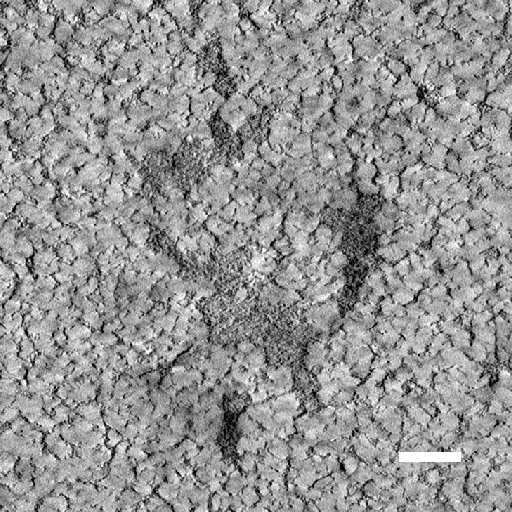

Supplement: Supplementary file 6 — Source data Fig. 1 [file 44318_2025_652_MOESM6_ESM.zip › Figure 1/1E/Fig1E_tiff_stack/modv0039.tif]

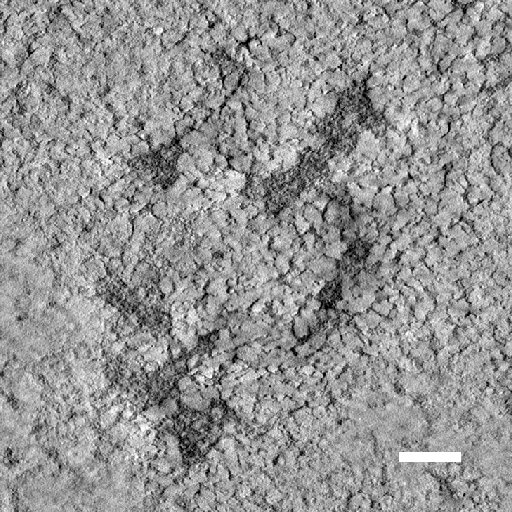

Supplement: Supplementary file 6 — Source data Fig. 1 [file 44318_2025_652_MOESM6_ESM.zip › Figure 1/1E/Fig1E_tiff_stack/modv0011.tif]

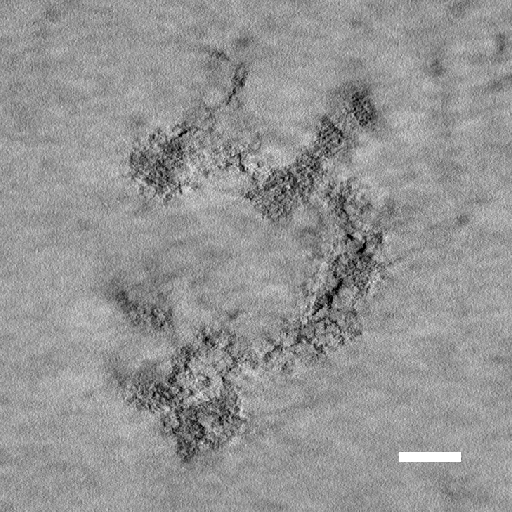

Supplement: Supplementary file 6 — Source data Fig. 1 [file 44318_2025_652_MOESM6_ESM.zip › Figure 1/1E/Fig1E_tiff_stack/modv0005.tif]

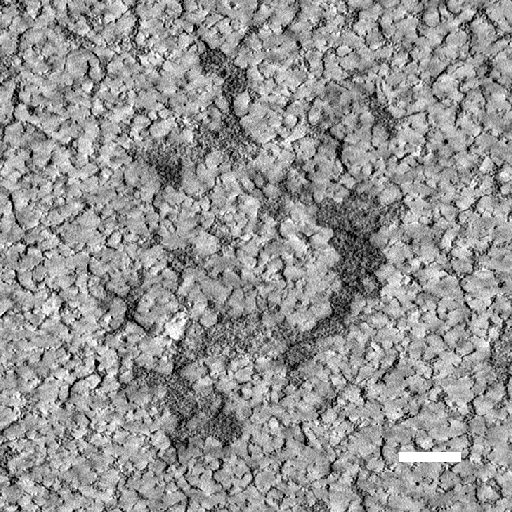

Supplement: Supplementary file 6 — Source data Fig. 1 [file 44318_2025_652_MOESM6_ESM.zip › Figure 1/1E/Fig1E_tiff_stack/modv0029.tif]

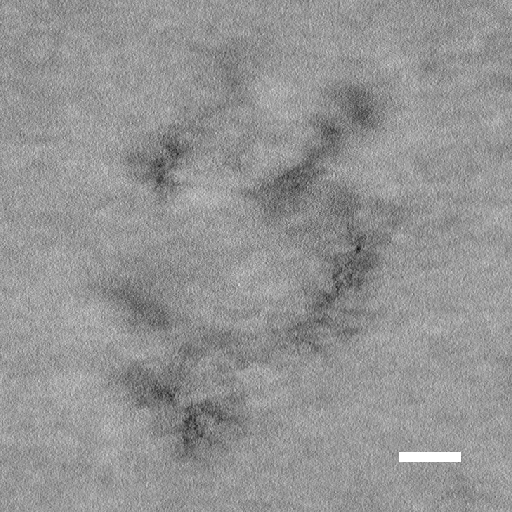

Supplement: Supplementary file 6 — Source data Fig. 1 [file 44318_2025_652_MOESM6_ESM.zip › Figure 1/1E/Fig1E_tiff_stack/modv0001.tif]

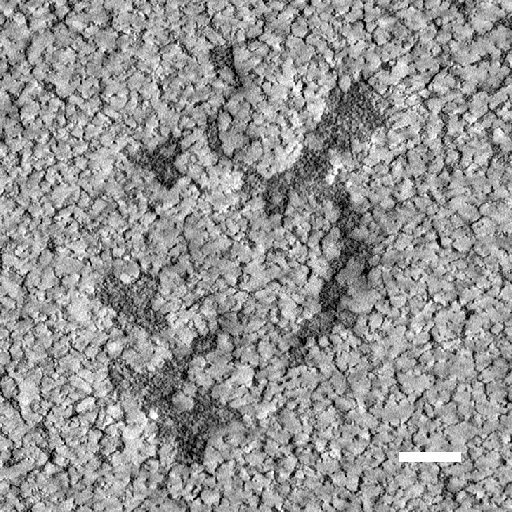

Supplement: Supplementary file 6 — Source data Fig. 1 [file 44318_2025_652_MOESM6_ESM.zip › Figure 1/1E/Fig1E_tiff_stack/modv0015.tif]

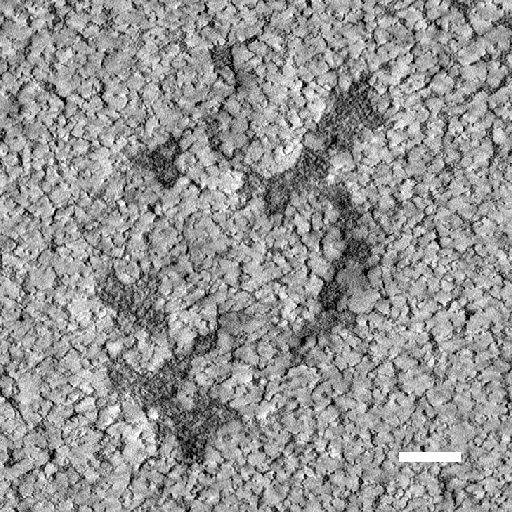

Supplement: Supplementary file 6 — Source data Fig. 1 [file 44318_2025_652_MOESM6_ESM.zip › Figure 1/1E/Fig1E_tiff_stack/modv0014.tif]

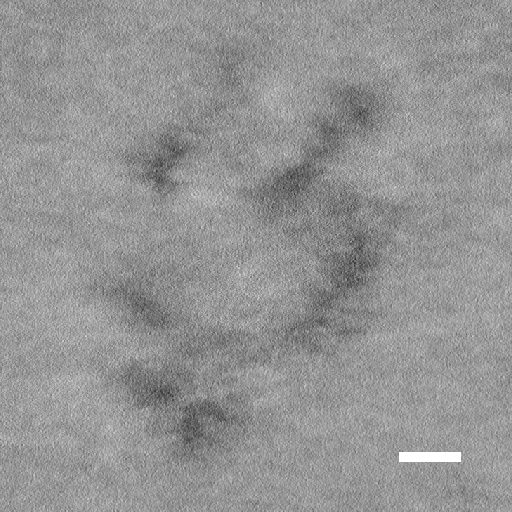

Supplement: Supplementary file 6 — Source data Fig. 1 [file 44318_2025_652_MOESM6_ESM.zip › Figure 1/1E/Fig1E_tiff_stack/modv0000.tif]

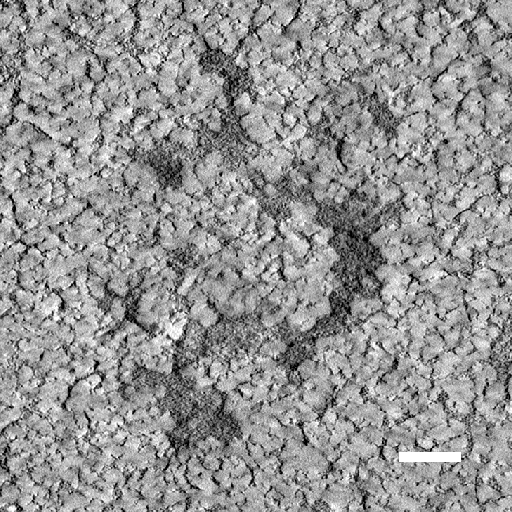

Supplement: Supplementary file 6 — Source data Fig. 1 [file 44318_2025_652_MOESM6_ESM.zip › Figure 1/1E/Fig1E_tiff_stack/modv0028.tif]

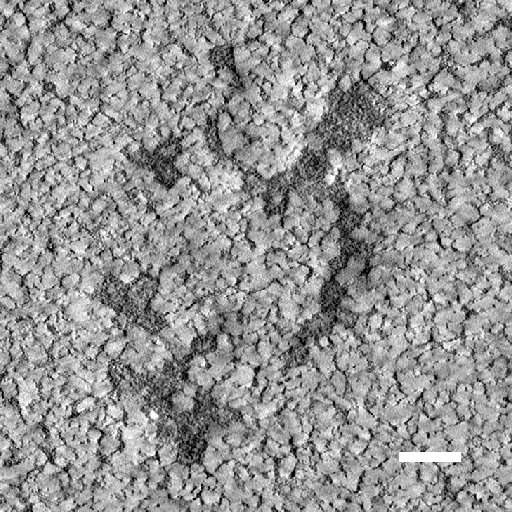

Supplement: Supplementary file 6 — Source data Fig. 1 [file 44318_2025_652_MOESM6_ESM.zip › Figure 1/1E/Fig1E_tiff_stack/modv0016.tif]

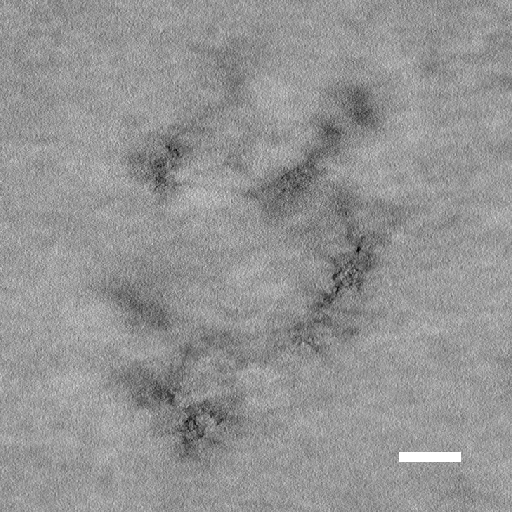

Supplement: Supplementary file 6 — Source data Fig. 1 [file 44318_2025_652_MOESM6_ESM.zip › Figure 1/1E/Fig1E_tiff_stack/modv0002.tif]

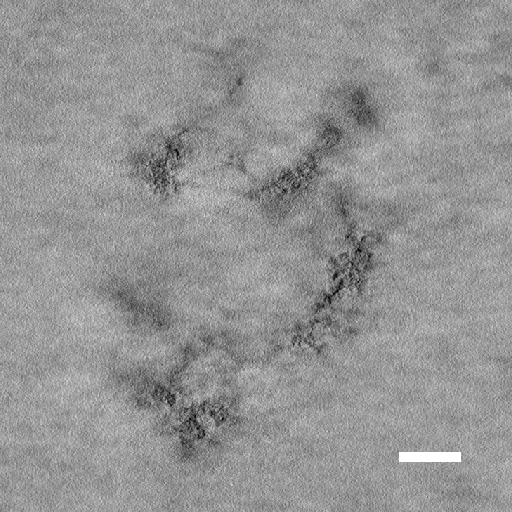

Supplement: Supplementary file 6 — Source data Fig. 1 [file 44318_2025_652_MOESM6_ESM.zip › Figure 1/1E/Fig1E_tiff_stack/modv0003.tif]

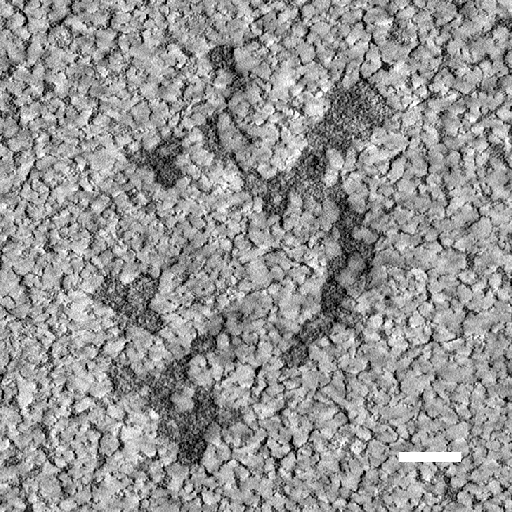

Supplement: Supplementary file 6 — Source data Fig. 1 [file 44318_2025_652_MOESM6_ESM.zip › Figure 1/1E/Fig1E_tiff_stack/modv0017.tif]

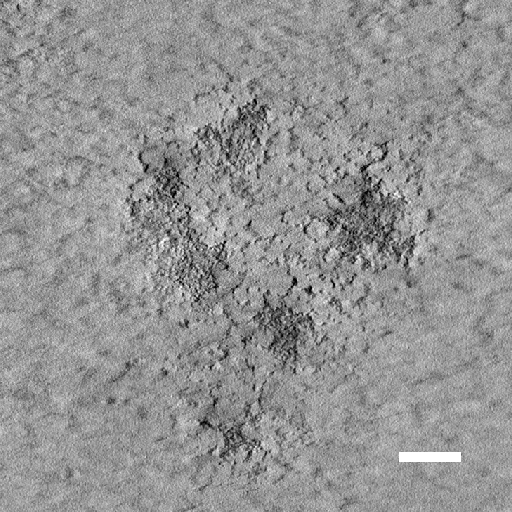

Supplement: Supplementary file 6 — Source data Fig. 1 [file 44318_2025_652_MOESM6_ESM.zip › Figure 1/1E/Fig1E_tiff_stack/modv0058.tif]

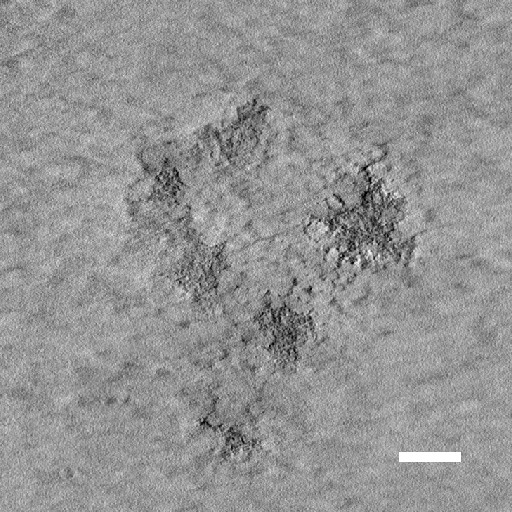

Supplement: Supplementary file 6 — Source data Fig. 1 [file 44318_2025_652_MOESM6_ESM.zip › Figure 1/1E/Fig1E_tiff_stack/modv0059.tif]

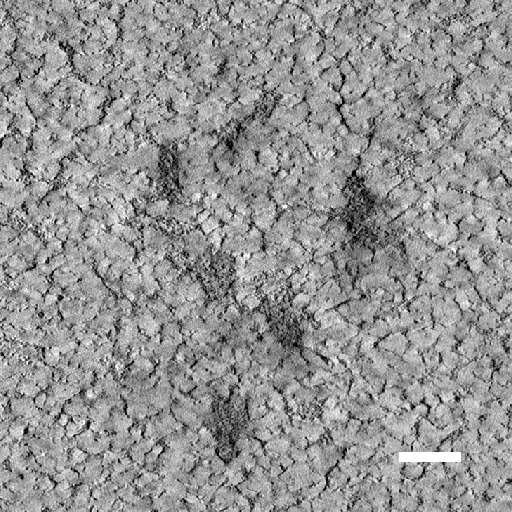

Supplement: Supplementary file 6 — Source data Fig. 1 [file 44318_2025_652_MOESM6_ESM.zip › Figure 1/1E/Fig1E_tiff_stack/modv0049.tif]

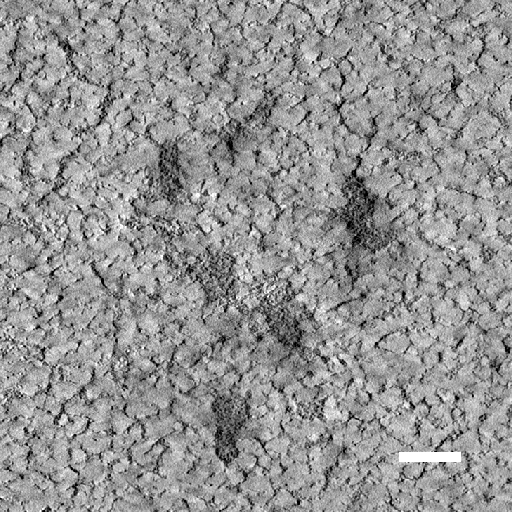

Supplement: Supplementary file 6 — Source data Fig. 1 [file 44318_2025_652_MOESM6_ESM.zip › Figure 1/1E/Fig1E_tiff_stack/modv0048.tif]

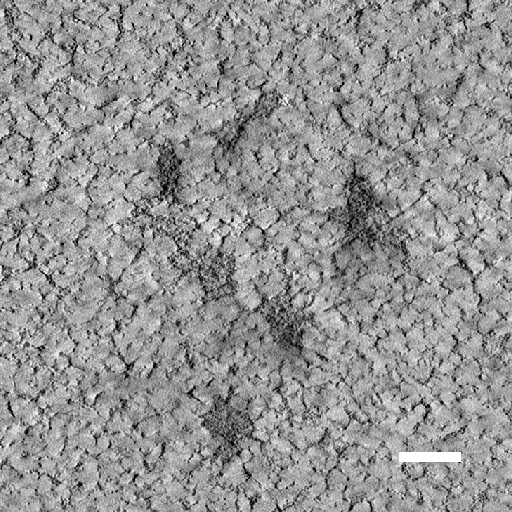

Supplement: Supplementary file 6 — Source data Fig. 1 [file 44318_2025_652_MOESM6_ESM.zip › Figure 1/1E/Fig1E_tiff_stack/modv0051.tif]

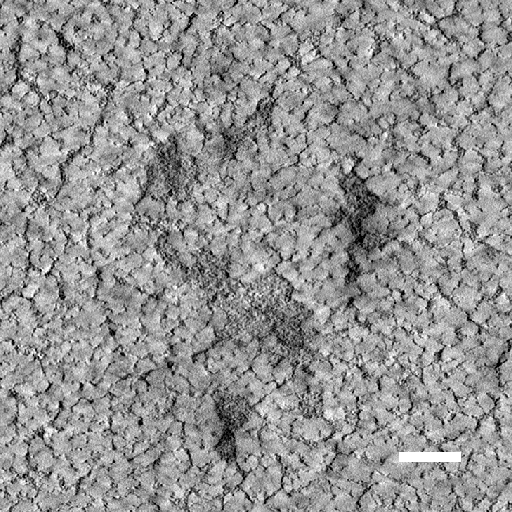

Supplement: Supplementary file 6 — Source data Fig. 1 [file 44318_2025_652_MOESM6_ESM.zip › Figure 1/1E/Fig1E_tiff_stack/modv0045.tif]

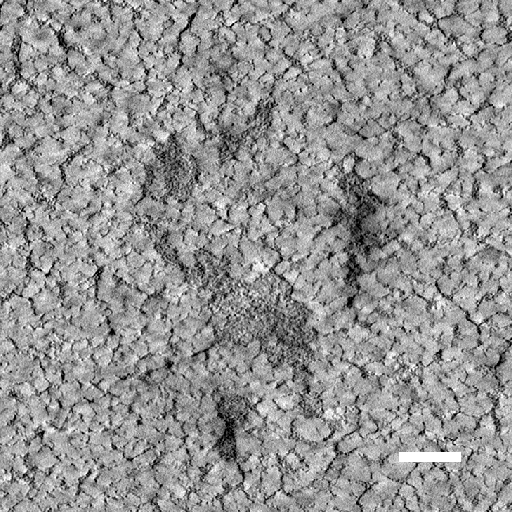

Supplement: Supplementary file 6 — Source data Fig. 1 [file 44318_2025_652_MOESM6_ESM.zip › Figure 1/1E/Fig1E_tiff_stack/modv0044.tif]

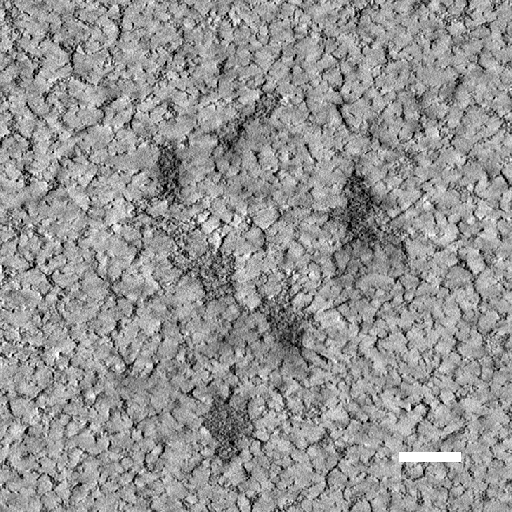

Supplement: Supplementary file 6 — Source data Fig. 1 [file 44318_2025_652_MOESM6_ESM.zip › Figure 1/1E/Fig1E_tiff_stack/modv0050.tif]

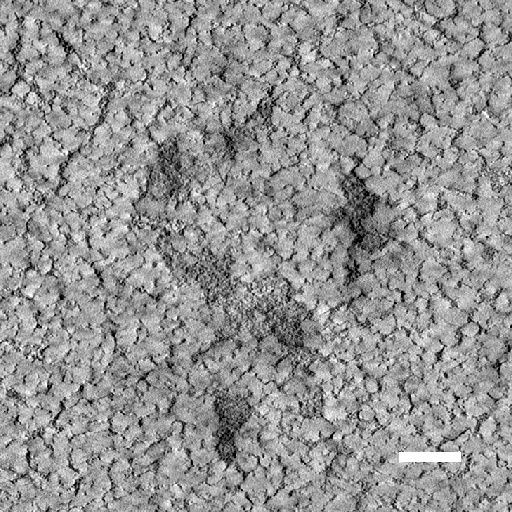

Supplement: Supplementary file 6 — Source data Fig. 1 [file 44318_2025_652_MOESM6_ESM.zip › Figure 1/1E/Fig1E_tiff_stack/modv0046.tif]

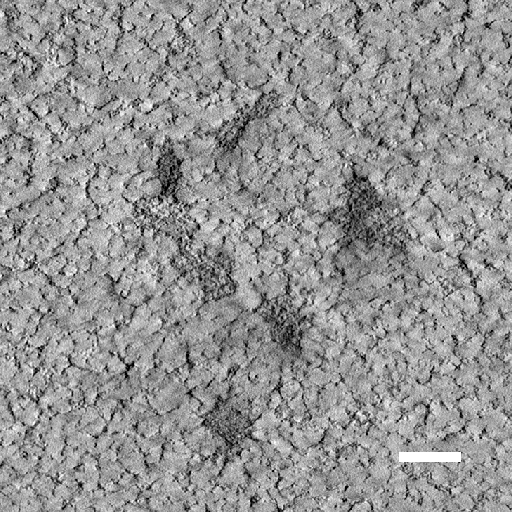

Supplement: Supplementary file 6 — Source data Fig. 1 [file 44318_2025_652_MOESM6_ESM.zip › Figure 1/1E/Fig1E_tiff_stack/modv0052.tif]

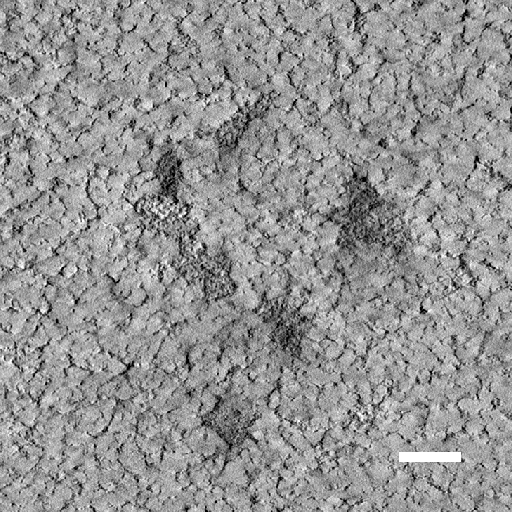

Supplement: Supplementary file 6 — Source data Fig. 1 [file 44318_2025_652_MOESM6_ESM.zip › Figure 1/1E/Fig1E_tiff_stack/modv0053.tif]

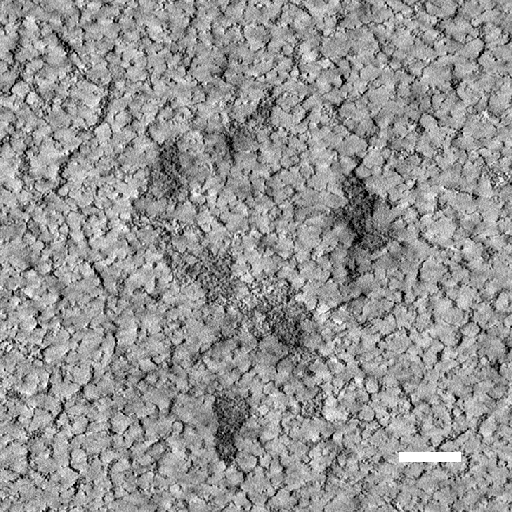

Supplement: Supplementary file 6 — Source data Fig. 1 [file 44318_2025_652_MOESM6_ESM.zip › Figure 1/1E/Fig1E_tiff_stack/modv0047.tif]

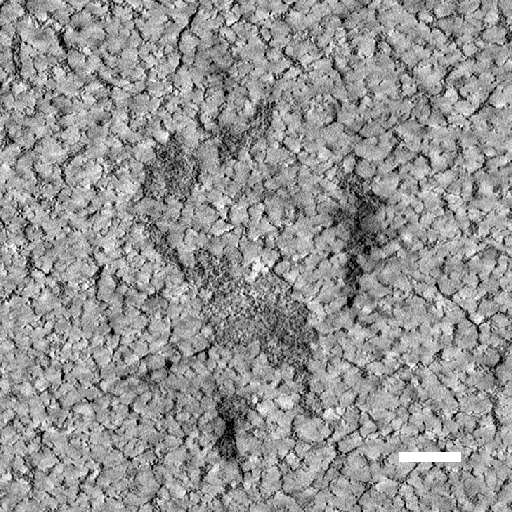

Supplement: Supplementary file 6 — Source data Fig. 1 [file 44318_2025_652_MOESM6_ESM.zip › Figure 1/1E/Fig1E_tiff_stack/modv0043.tif]

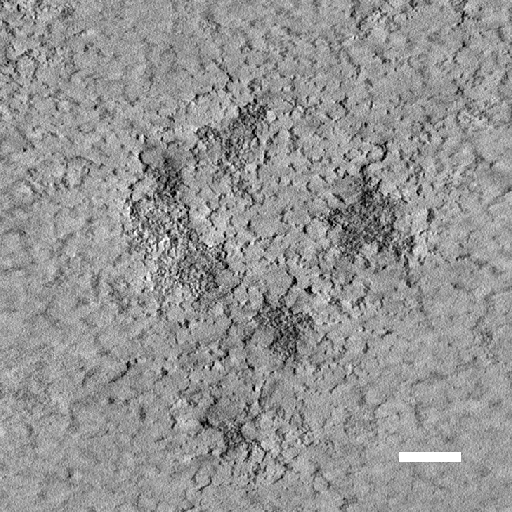

Supplement: Supplementary file 6 — Source data Fig. 1 [file 44318_2025_652_MOESM6_ESM.zip › Figure 1/1E/Fig1E_tiff_stack/modv0057.tif]

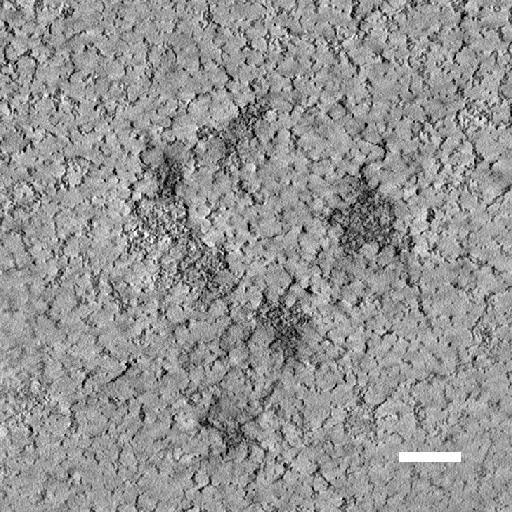

Supplement: Supplementary file 6 — Source data Fig. 1 [file 44318_2025_652_MOESM6_ESM.zip › Figure 1/1E/Fig1E_tiff_stack/modv0056.tif]

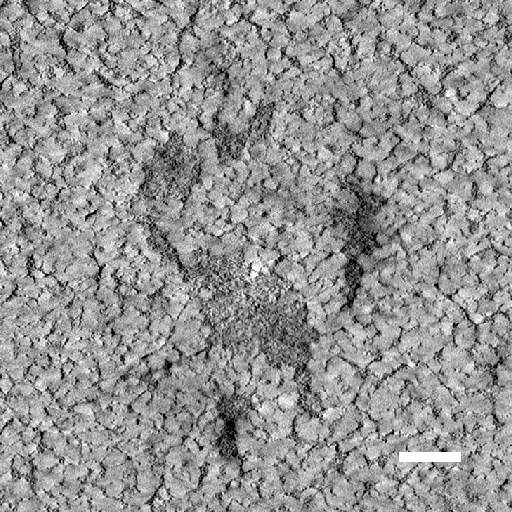

Supplement: Supplementary file 6 — Source data Fig. 1 [file 44318_2025_652_MOESM6_ESM.zip › Figure 1/1E/Fig1E_tiff_stack/modv0042.tif]

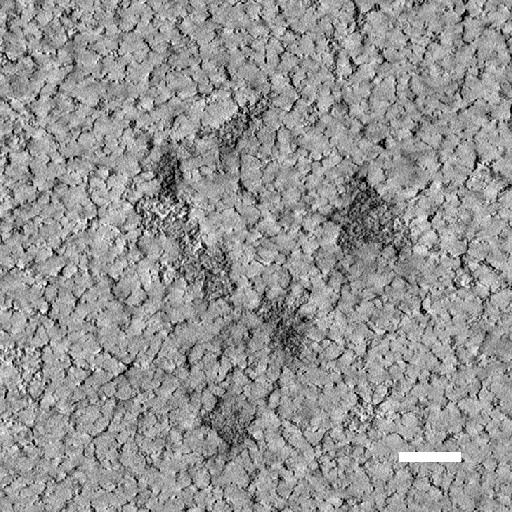

Supplement: Supplementary file 6 — Source data Fig. 1 [file 44318_2025_652_MOESM6_ESM.zip › Figure 1/1E/Fig1E_tiff_stack/modv0054.tif]

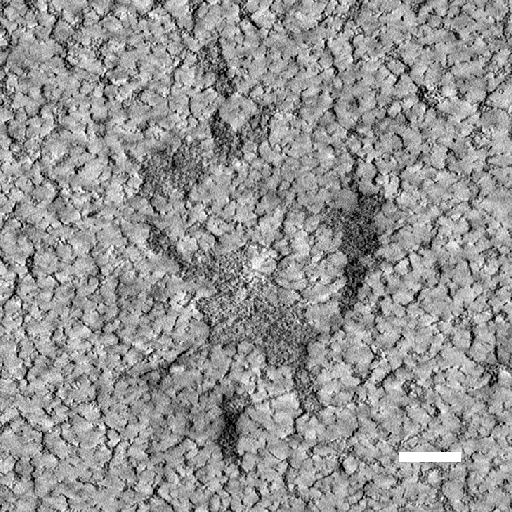

Supplement: Supplementary file 6 — Source data Fig. 1 [file 44318_2025_652_MOESM6_ESM.zip › Figure 1/1E/Fig1E_tiff_stack/modv0040.tif]

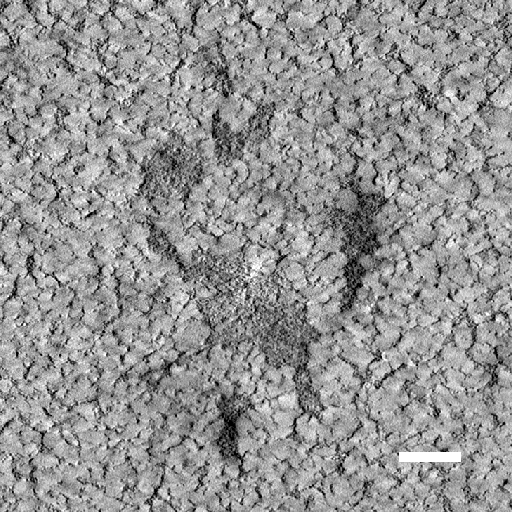

Supplement: Supplementary file 6 — Source data Fig. 1 [file 44318_2025_652_MOESM6_ESM.zip › Figure 1/1E/Fig1E_tiff_stack/modv0041.tif]

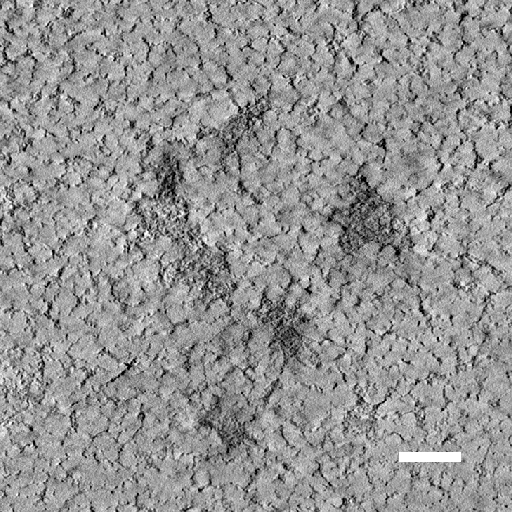

Supplement: Supplementary file 6 — Source data Fig. 1 [file 44318_2025_652_MOESM6_ESM.zip › Figure 1/1E/Fig1E_tiff_stack/modv0055.tif]

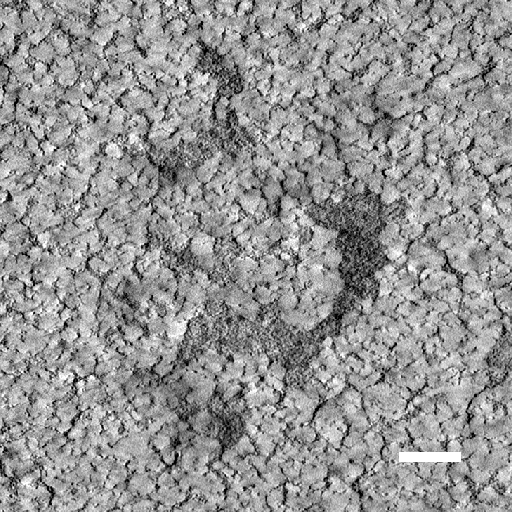

Supplement: Supplementary file 6 — Source data Fig. 1 [file 44318_2025_652_MOESM6_ESM.zip › Figure 1/1E/Fig1E_tiff_stack/modv0032.tif]

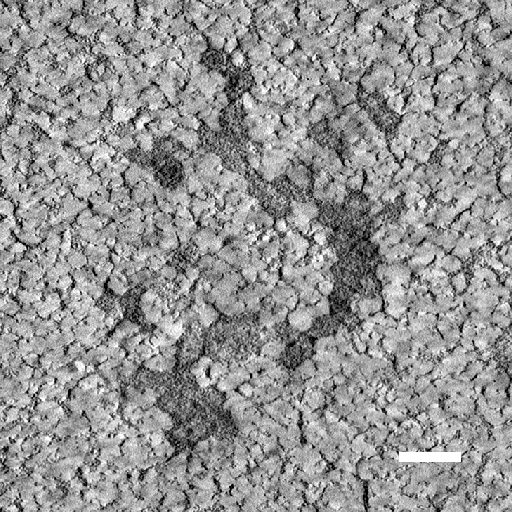

Supplement: Supplementary file 6 — Source data Fig. 1 [file 44318_2025_652_MOESM6_ESM.zip › Figure 1/1E/Fig1E_tiff_stack/modv0026.tif]

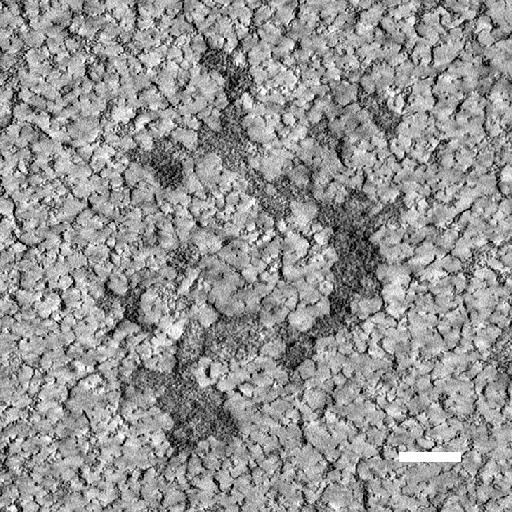

Supplement: Supplementary file 6 — Source data Fig. 1 [file 44318_2025_652_MOESM6_ESM.zip › Figure 1/1E/Fig1E_tiff_stack/modv0027.tif]

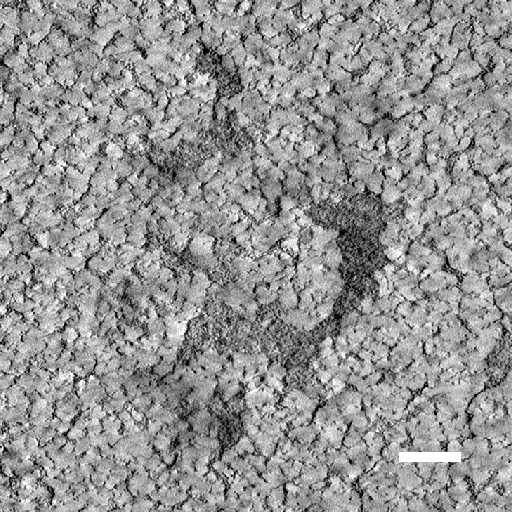

Supplement: Supplementary file 6 — Source data Fig. 1 [file 44318_2025_652_MOESM6_ESM.zip › Figure 1/1E/Fig1E_tiff_stack/modv0033.tif]

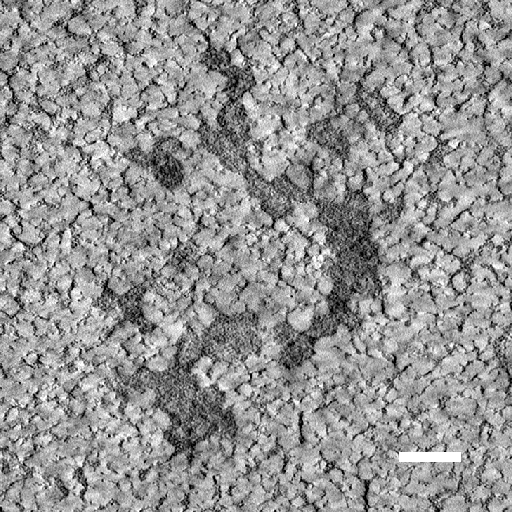

Supplement: Supplementary file 6 — Source data Fig. 1 [file 44318_2025_652_MOESM6_ESM.zip › Figure 1/1E/Fig1E_tiff_stack/modv0025.tif]

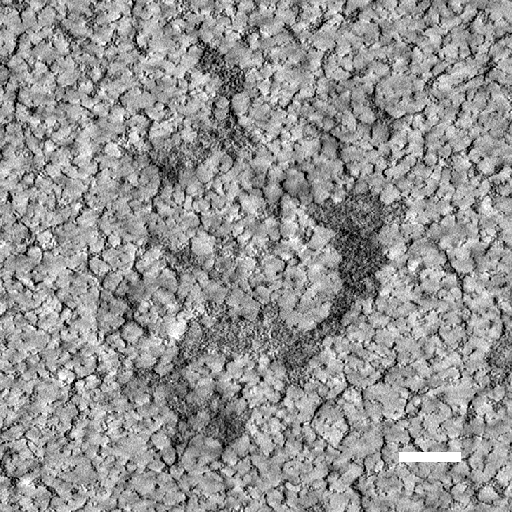

Supplement: Supplementary file 6 — Source data Fig. 1 [file 44318_2025_652_MOESM6_ESM.zip › Figure 1/1E/Fig1E_tiff_stack/modv0031.tif]

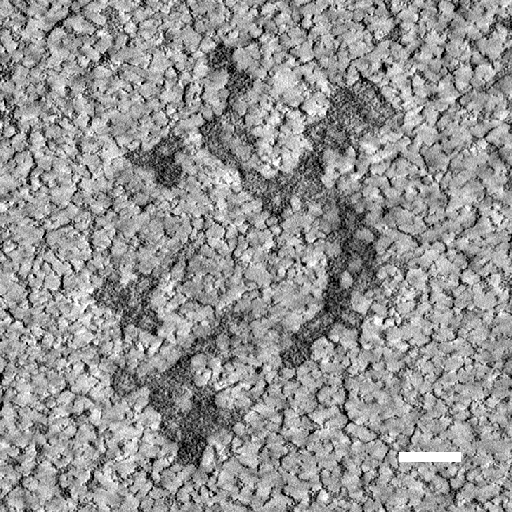

Supplement: Supplementary file 6 — Source data Fig. 1 [file 44318_2025_652_MOESM6_ESM.zip › Figure 1/1E/Fig1E_tiff_stack/modv0019.tif]

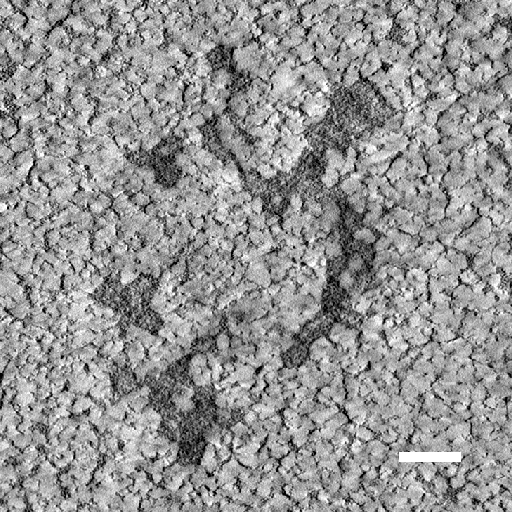

Supplement: Supplementary file 6 — Source data Fig. 1 [file 44318_2025_652_MOESM6_ESM.zip › Figure 1/1E/Fig1E_tiff_stack/modv0018.tif]

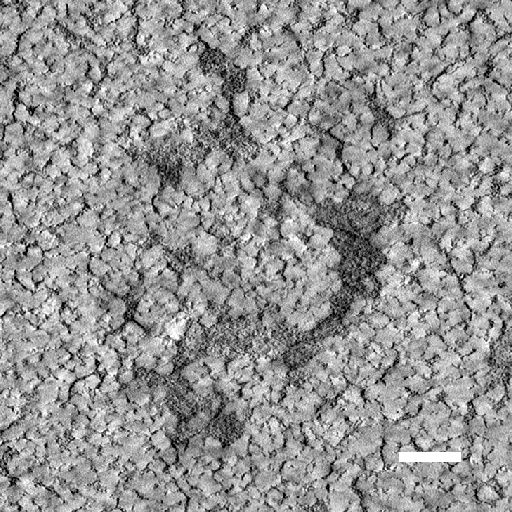

Supplement: Supplementary file 6 — Source data Fig. 1 [file 44318_2025_652_MOESM6_ESM.zip › Figure 1/1E/Fig1E_tiff_stack/modv0030.tif]

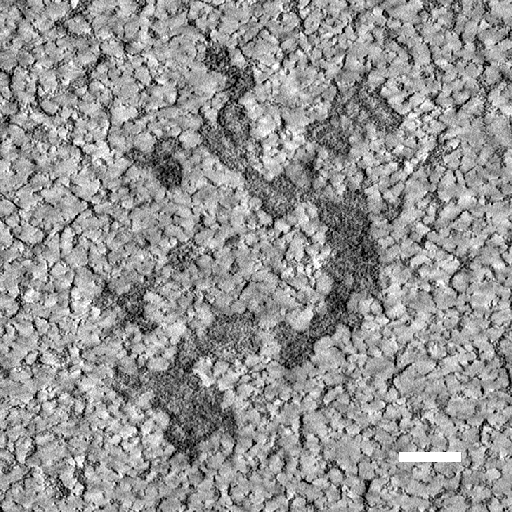

Supplement: Supplementary file 6 — Source data Fig. 1 [file 44318_2025_652_MOESM6_ESM.zip › Figure 1/1E/Fig1E_tiff_stack/modv0024.tif]

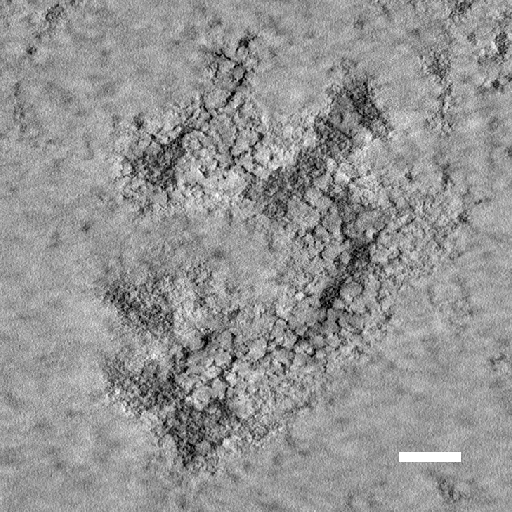

Supplement: Supplementary file 6 — Source data Fig. 1 [file 44318_2025_652_MOESM6_ESM.zip › Figure 1/1E/Fig1E_tiff_stack/modv0008.tif]

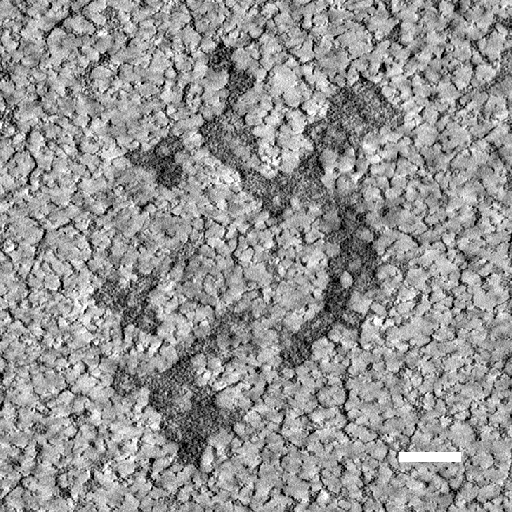

Supplement: Supplementary file 6 — Source data Fig. 1 [file 44318_2025_652_MOESM6_ESM.zip › Figure 1/1E/Fig1E_tiff_stack/modv0020.tif]

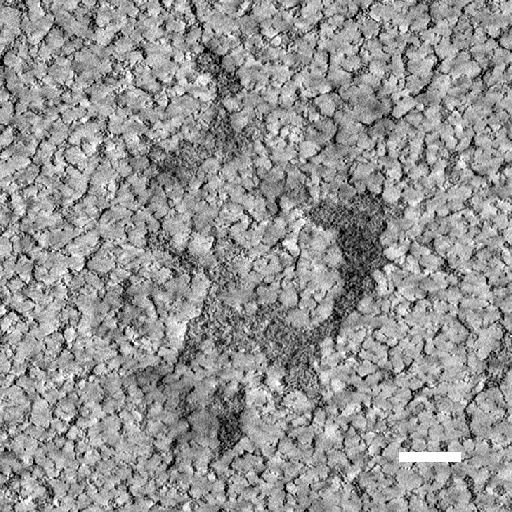

Supplement: Supplementary file 6 — Source data Fig. 1 [file 44318_2025_652_MOESM6_ESM.zip › Figure 1/1E/Fig1E_tiff_stack/modv0034.tif]

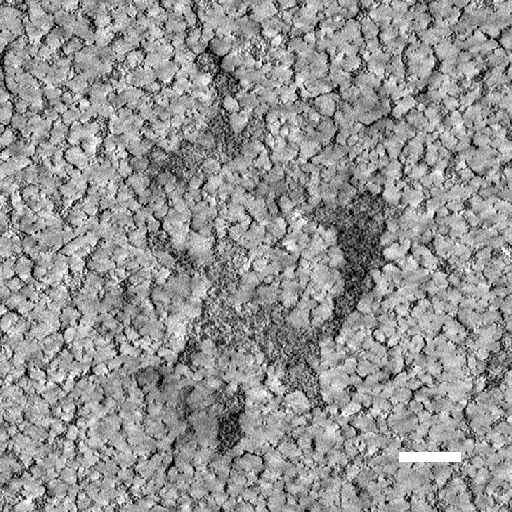

Supplement: Supplementary file 6 — Source data Fig. 1 [file 44318_2025_652_MOESM6_ESM.zip › Figure 1/1E/Fig1E_tiff_stack/modv0035.tif]

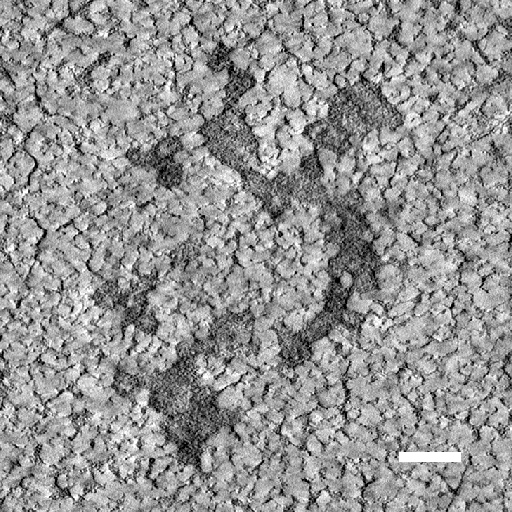

Supplement: Supplementary file 6 — Source data Fig. 1 [file 44318_2025_652_MOESM6_ESM.zip › Figure 1/1E/Fig1E_tiff_stack/modv0021.tif]

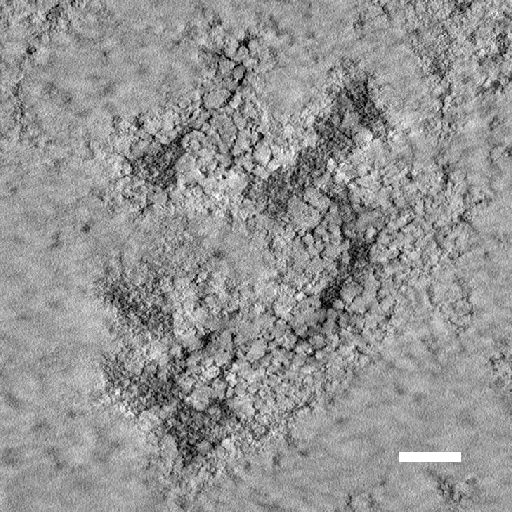

Supplement: Supplementary file 6 — Source data Fig. 1 [file 44318_2025_652_MOESM6_ESM.zip › Figure 1/1E/Fig1E_tiff_stack/modv0009.tif]

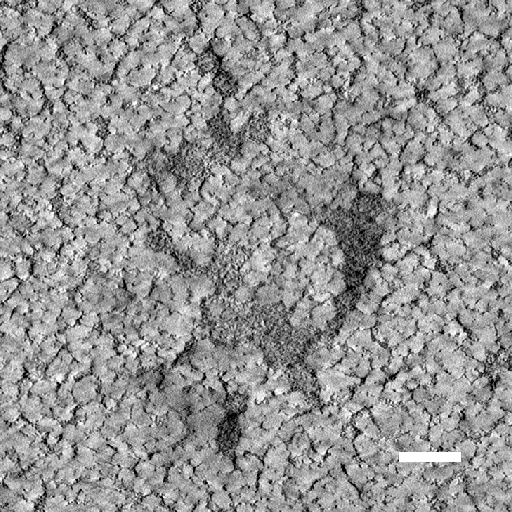

Supplement: Supplementary file 6 — Source data Fig. 1 [file 44318_2025_652_MOESM6_ESM.zip › Figure 1/1E/Fig1E_tiff_stack/modv0037.tif]

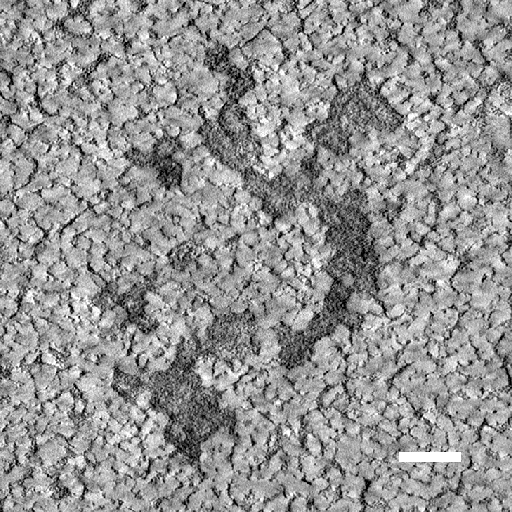

Supplement: Supplementary file 6 — Source data Fig. 1 [file 44318_2025_652_MOESM6_ESM.zip › Figure 1/1E/Fig1E_tiff_stack/modv0023.tif]

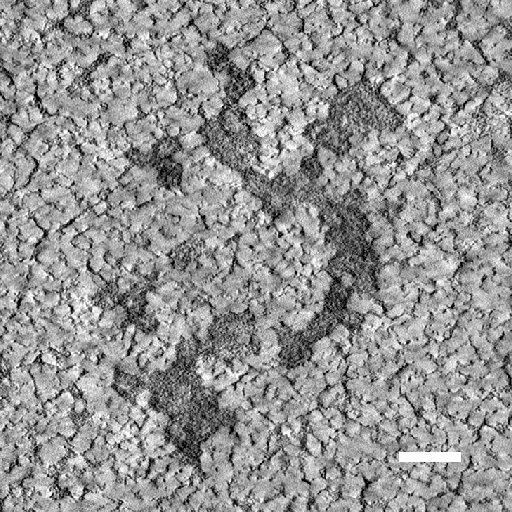

Supplement: Supplementary file 6 — Source data Fig. 1 [file 44318_2025_652_MOESM6_ESM.zip › Figure 1/1E/Fig1E_tiff_stack/modv0022.tif]

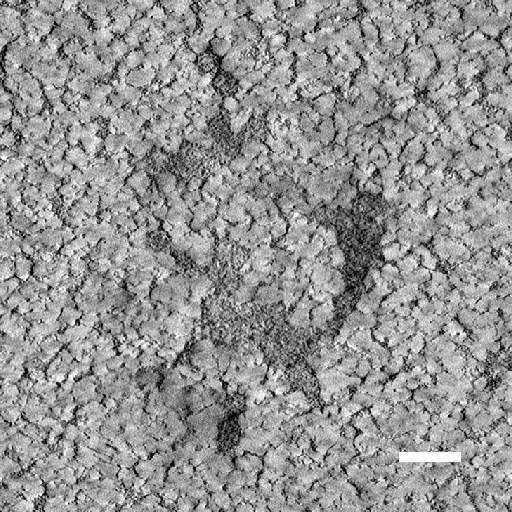

Supplement: Supplementary file 6 — Source data Fig. 1 [file 44318_2025_652_MOESM6_ESM.zip › Figure 1/1E/Fig1E_tiff_stack/modv0036.tif]

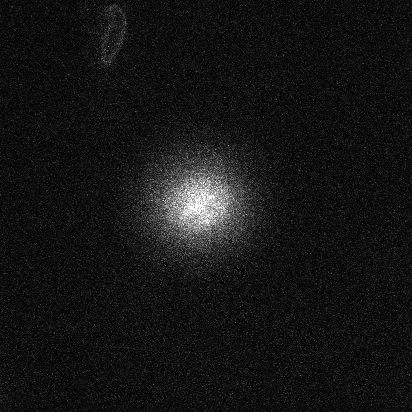

Supplement: Supplementary file 7 — Source data Fig. 2 [file 44318_2025_652_MOESM7_ESM.zip › Figure 2/2A/Fig2a-500nM_Raw.tif]

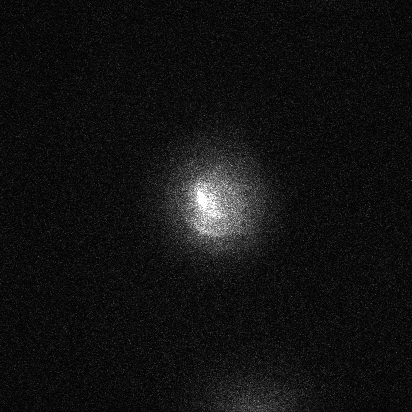

Supplement: Supplementary file 7 — Source data Fig. 2 [file 44318_2025_652_MOESM7_ESM.zip › Figure 2/2A/Fig2a-DMSO_Raw.tif]

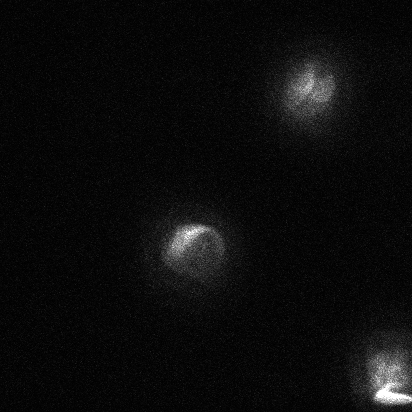

Supplement: Supplementary file 7 — Source data Fig. 2 [file 44318_2025_652_MOESM7_ESM.zip › Figure 2/2A/Fig2a-5nM_Raw.tif]

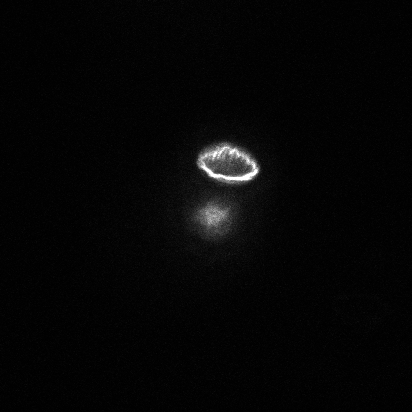

Supplement: Supplementary file 7 — Source data Fig. 2 [file 44318_2025_652_MOESM7_ESM.zip › Figure 2/2A/Fig2a-50nM_Raw.tif]

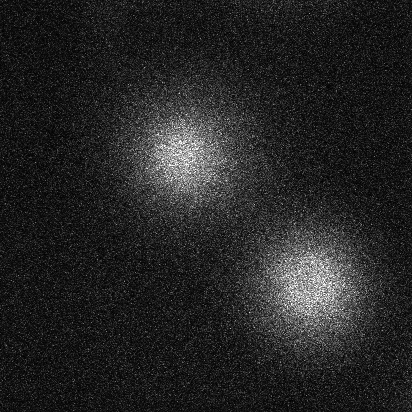

Supplement: Supplementary file 7 — Source data Fig. 2 [file 44318_2025_652_MOESM7_ESM.zip › Figure 2/2C/Fig2c-50nM_Raw.tif]

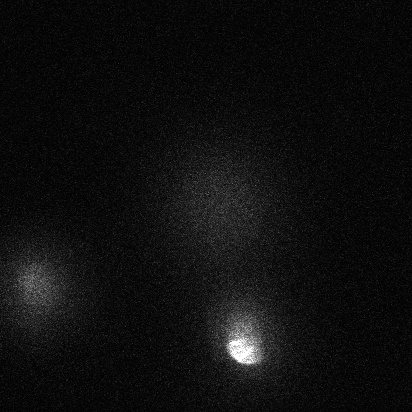

Supplement: Supplementary file 7 — Source data Fig. 2 [file 44318_2025_652_MOESM7_ESM.zip › Figure 2/2C/Fig2c-5nM_Raw.tif]

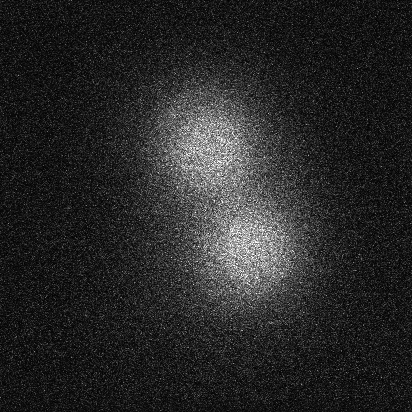

Supplement: Supplementary file 7 — Source data Fig. 2 [file 44318_2025_652_MOESM7_ESM.zip › Figure 2/2C/Fig2c-500nM_Raw.tif]

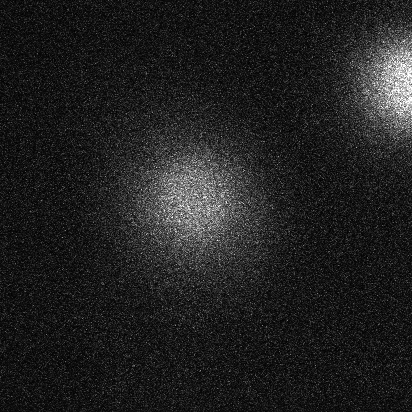

Supplement: Supplementary file 7 — Source data Fig. 2 [file 44318_2025_652_MOESM7_ESM.zip › Figure 2/2C/Fig2c-DMSO_Raw.tif]

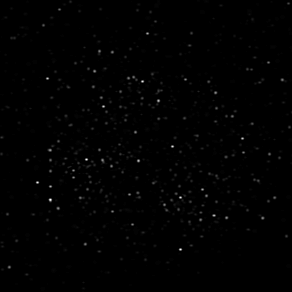

Supplement: Supplementary file 7 — Source data Fig. 2 [file 44318_2025_652_MOESM7_ESM.zip › Figure 2/2E/2E_c1_IN-eGFP_c2_SRRM2_c3_EdU.tif]

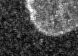

Supplement: Supplementary file 8 — Source data Fig. 3 [file 44318_2025_652_MOESM8_ESM.zip › Figure 3/3E/3E_c1_LMNB1_c2_eGFP-OR3_c3_IN-SNAP.tif]

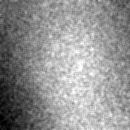

Supplement: Supplementary file 8 — Source data Fig. 3 [file 44318_2025_652_MOESM8_ESM.zip › Figure 3/3B/3B_c1_LMNB1_c2_eGFP-OR3_c3_IN-SNAP_c4_CA.tif]

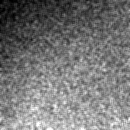

Supplement: Supplementary file 8 — Source data Fig. 3 [file 44318_2025_652_MOESM8_ESM.zip › Figure 3/3A/3A_c1_LMNB1_c2_eGFP-OR3_c3_IN-SNAP.tif]

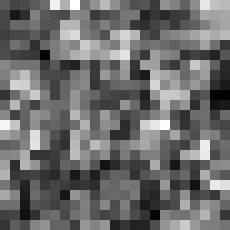

Supplement: Supplementary file 8 — Source data Fig. 3 [file 44318_2025_652_MOESM8_ESM.zip › Figure 3/3G/3G_c1_LMNB1_c2_eGFP-OR3_c3_IN-SNAP.tif]

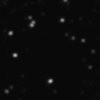

Supplement: Supplementary file 9 — Source data Fig. 4 [file 44318_2025_652_MOESM9_ESM.zip › Figure 4/4B/4B_c1_IN-eGFP_c2_mScarlet-OR3.tif]

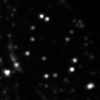

Supplement: Supplementary file 9 — Source data Fig. 4 [file 44318_2025_652_MOESM9_ESM.zip › Figure 4/4C/4C_c1_IN-eGFP_c2_mScarlet-OR3.tif]

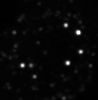

Supplement: Supplementary file 9 — Source data Fig. 4 [file 44318_2025_652_MOESM9_ESM.zip › Figure 4/4A/4A_c1_IN-eGFP_c2_mScarlet-OR3.tif]

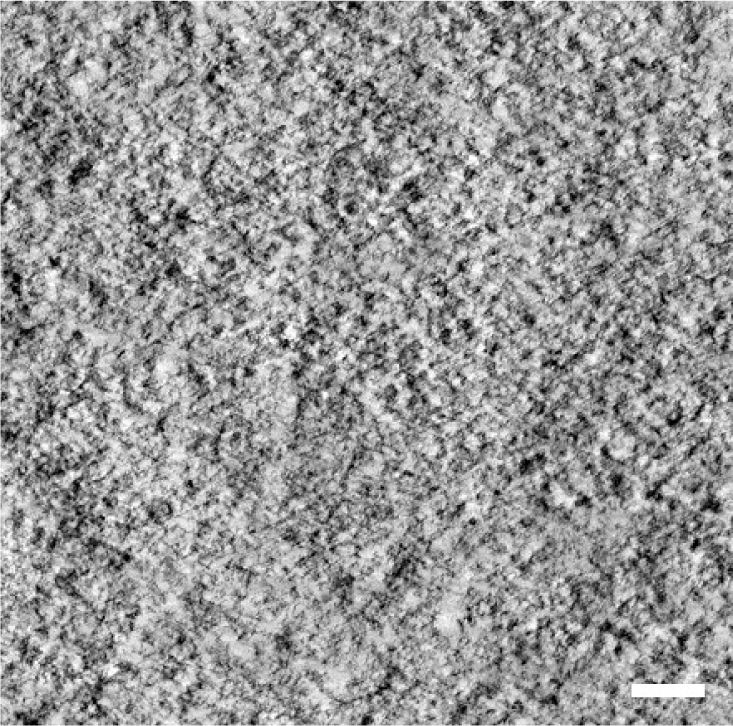

Supplement: Supplementary file 10 — Source data Fig. 5 [file 44318_2025_652_MOESM10_ESM.zip › Figure 5/5A/5A_ETii.tif]

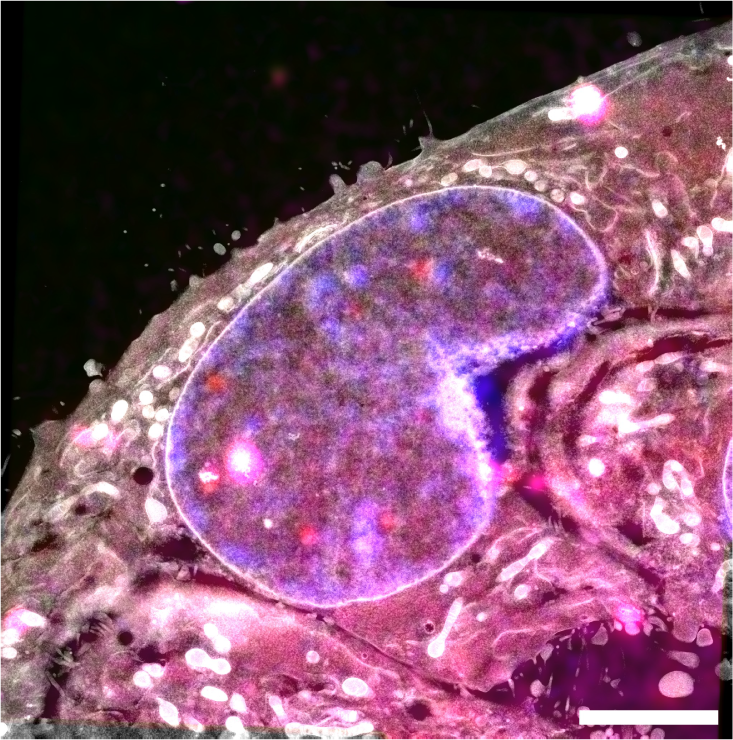

Supplement: Supplementary file 10 — Source data Fig. 5 [file 44318_2025_652_MOESM10_ESM.zip › Figure 5/5A/5A_overview.tif]

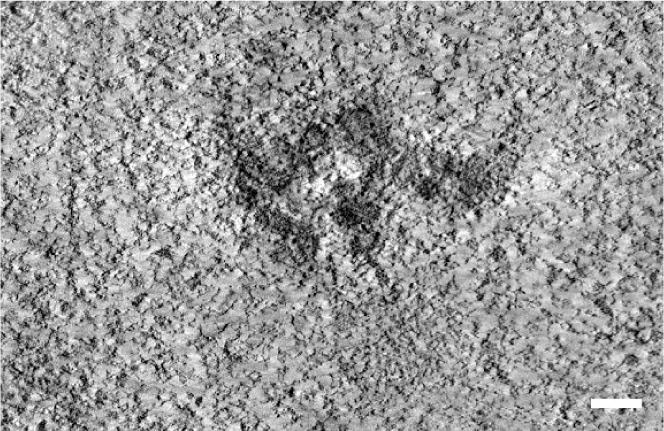

Supplement: Supplementary file 10 — Source data Fig. 5 [file 44318_2025_652_MOESM10_ESM.zip › Figure 5/5A/5A_ETi.tif]

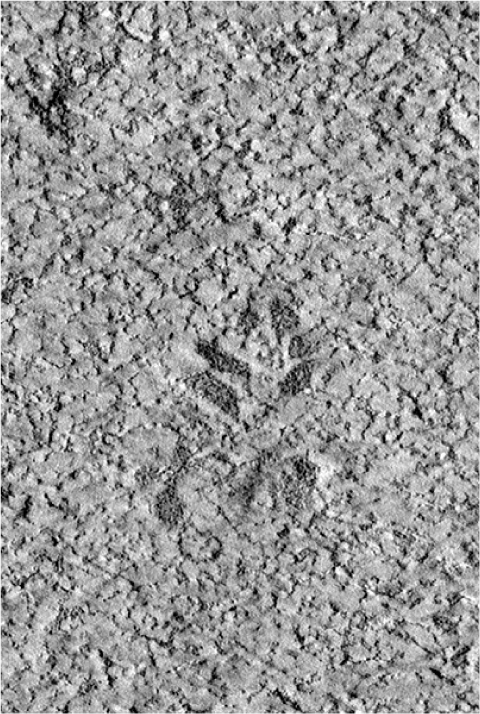

Supplement: Supplementary file 10 — Source data Fig. 5 [file 44318_2025_652_MOESM10_ESM.zip › Figure 5/5C/5C_ETii.tif]

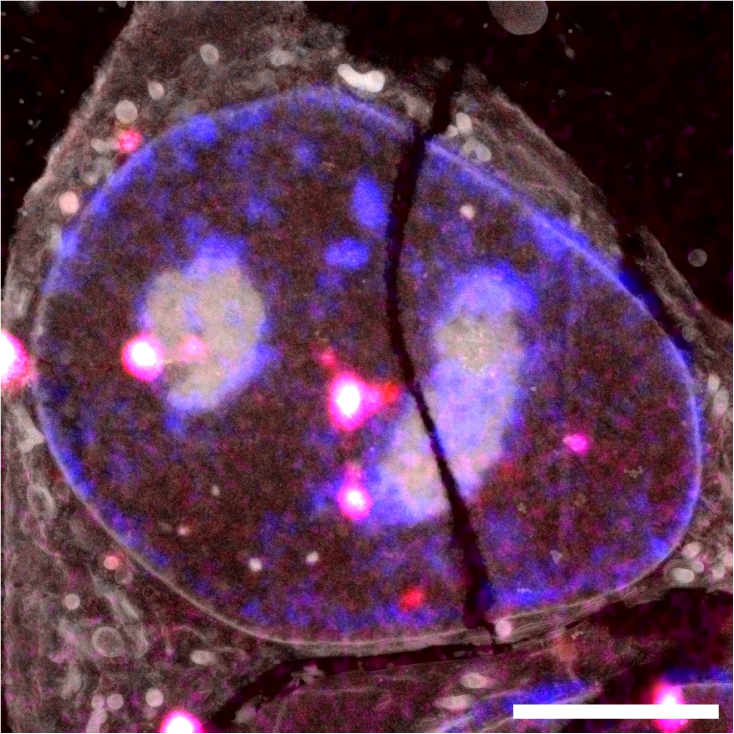

Supplement: Supplementary file 10 — Source data Fig. 5 [file 44318_2025_652_MOESM10_ESM.zip › Figure 5/5C/5C_overview.png]

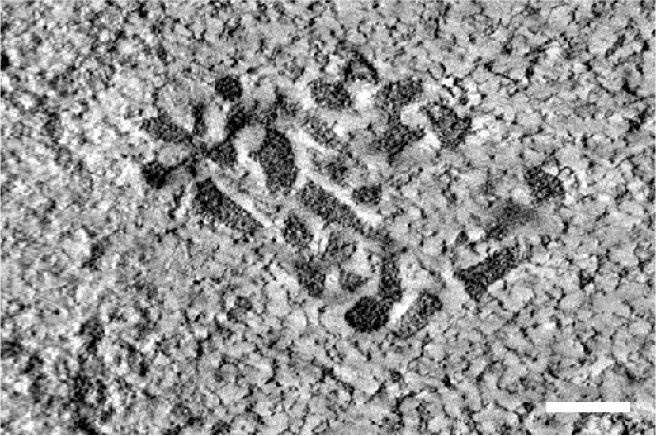

Supplement: Supplementary file 10 — Source data Fig. 5 [file 44318_2025_652_MOESM10_ESM.zip › Figure 5/5C/5C_ETi.tif]

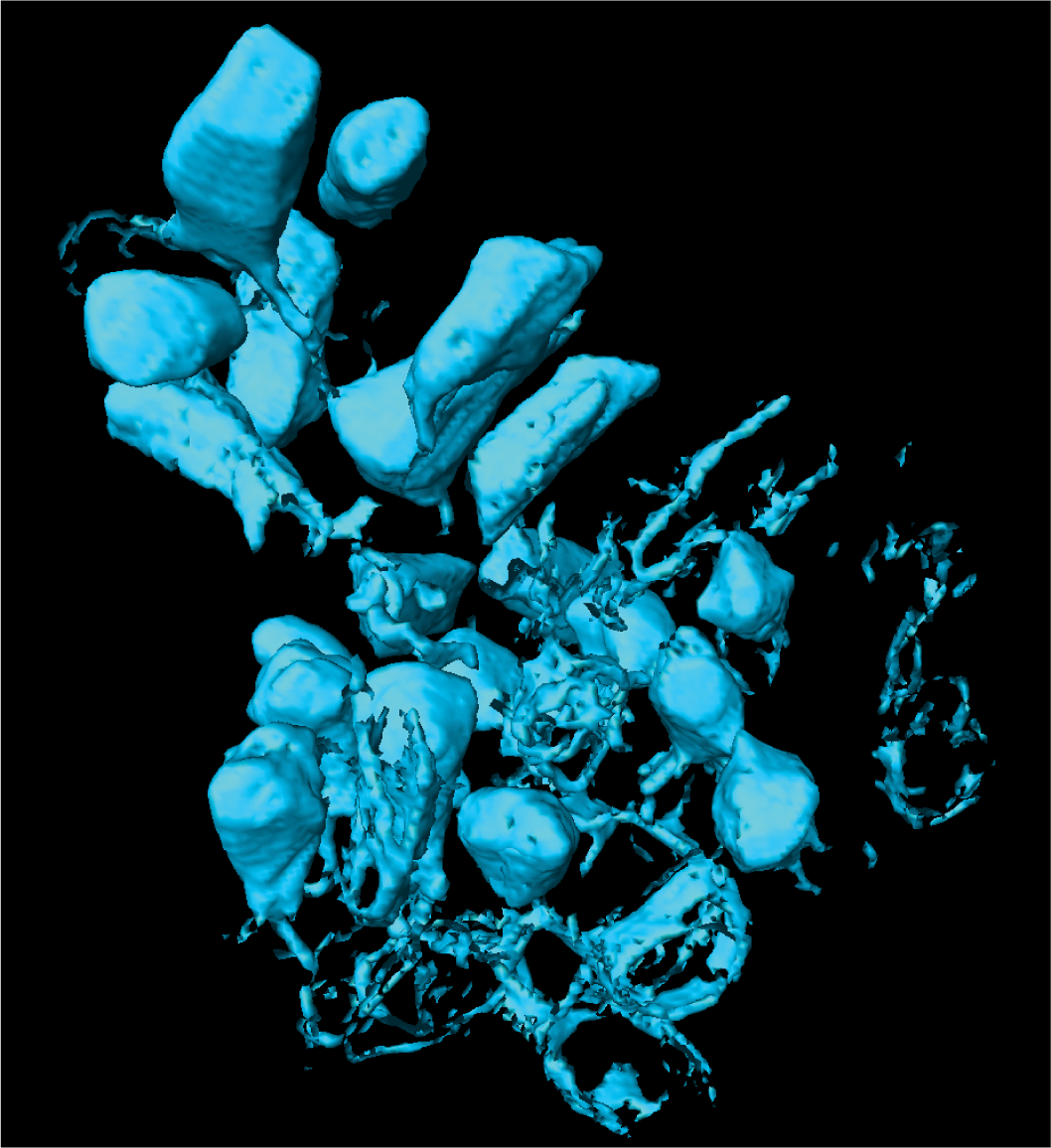

Supplement: Supplementary file 10 — Source data Fig. 5 [file 44318_2025_652_MOESM10_ESM.zip › Figure 5/5D/5D.tif]

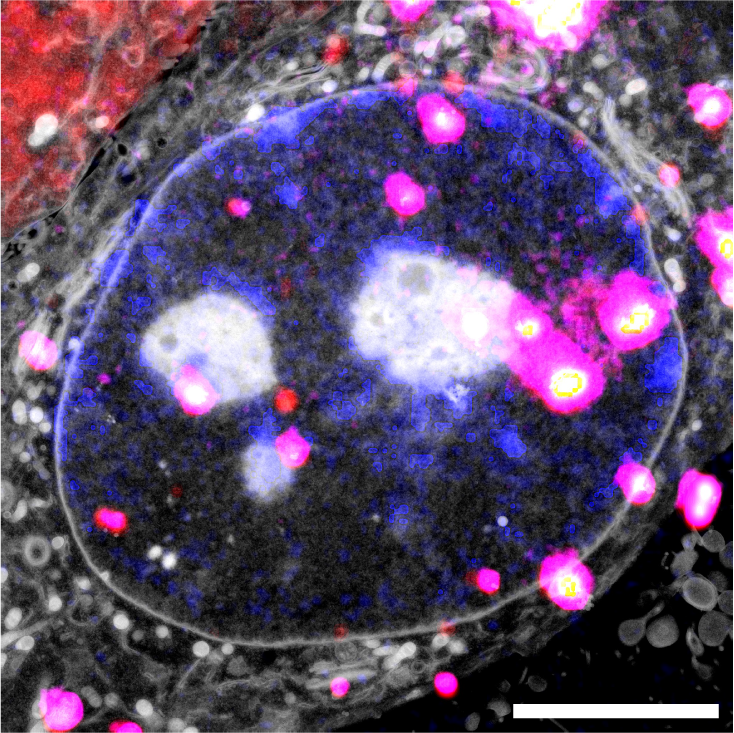

Supplement: Supplementary file 10 — Source data Fig. 5 [file 44318_2025_652_MOESM10_ESM.zip › Figure 5/5B/5B_overview.tif]

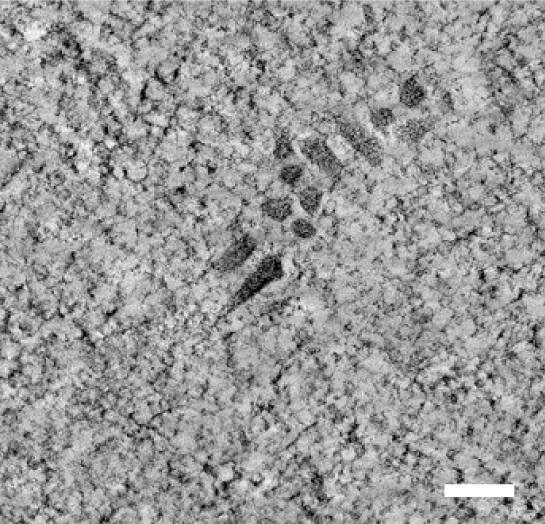

Supplement: Supplementary file 10 — Source data Fig. 5 [file 44318_2025_652_MOESM10_ESM.zip › Figure 5/5B/5B_ETii.tif]

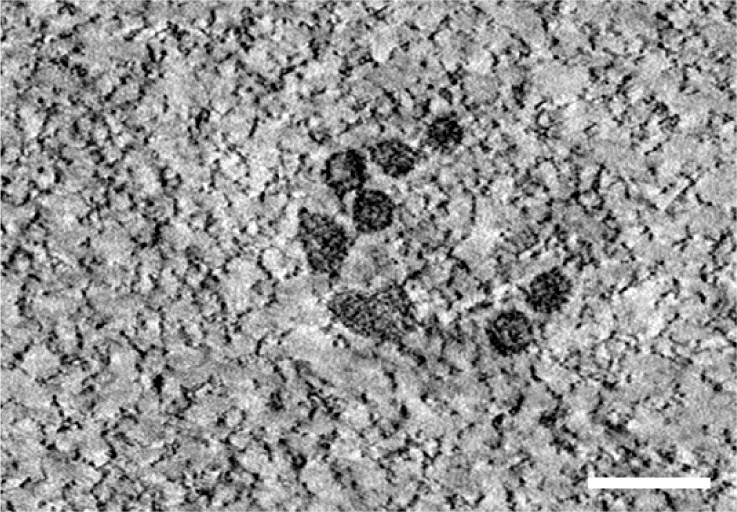

Supplement: Supplementary file 10 — Source data Fig. 5 [file 44318_2025_652_MOESM10_ESM.zip › Figure 5/5B/5B_ETi.tif]

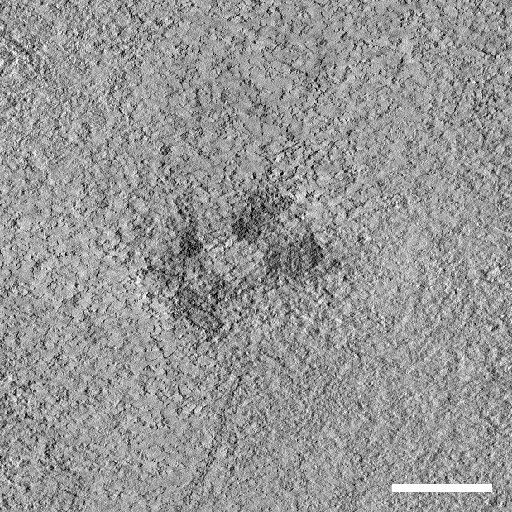

Supplement: Supplementary file 10 — Source data Fig. 5 [file 44318_2025_652_MOESM10_ESM.zip › Figure 5/5A/5A_ETi_stack/modv0329.jpg]

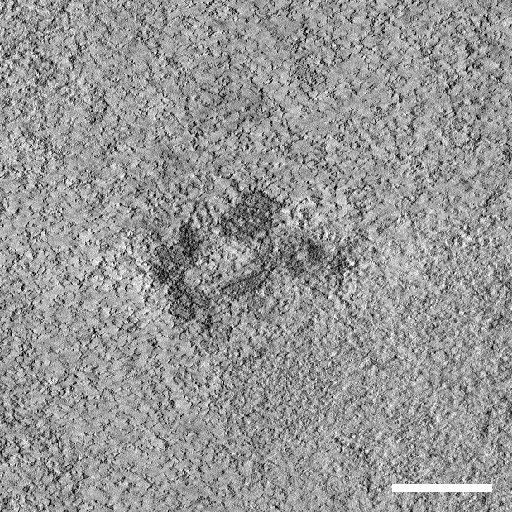

Supplement: Supplementary file 10 — Source data Fig. 5 [file 44318_2025_652_MOESM10_ESM.zip › Figure 5/5A/5A_ETi_stack/modv0301.jpg]

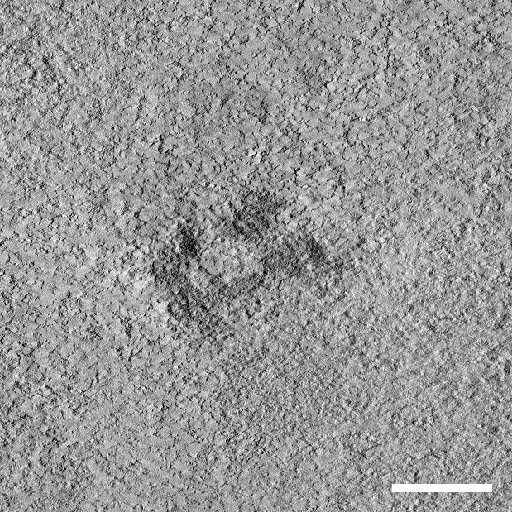

Supplement: Supplementary file 10 — Source data Fig. 5 [file 44318_2025_652_MOESM10_ESM.zip › Figure 5/5A/5A_ETi_stack/modv0315.jpg]

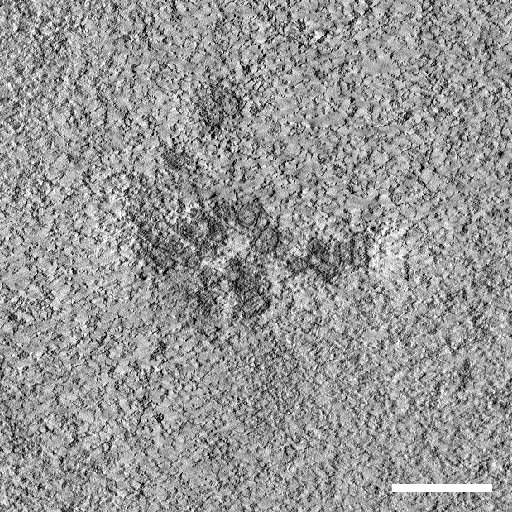

Supplement: Supplementary file 10 — Source data Fig. 5 [file 44318_2025_652_MOESM10_ESM.zip › Figure 5/5A/5A_ETi_stack/modv0249.jpg]

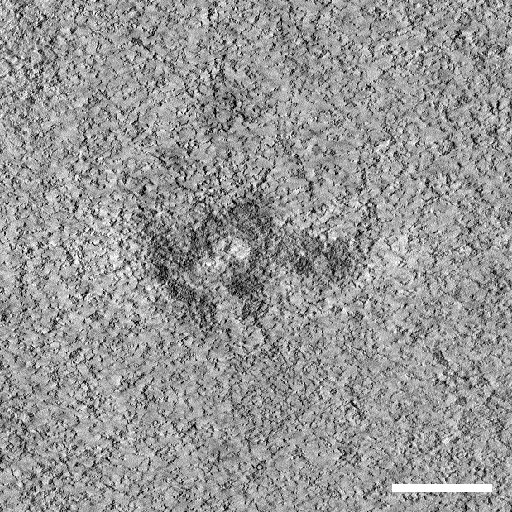

Supplement: Supplementary file 10 — Source data Fig. 5 [file 44318_2025_652_MOESM10_ESM.zip › Figure 5/5A/5A_ETi_stack/modv0275.jpg]

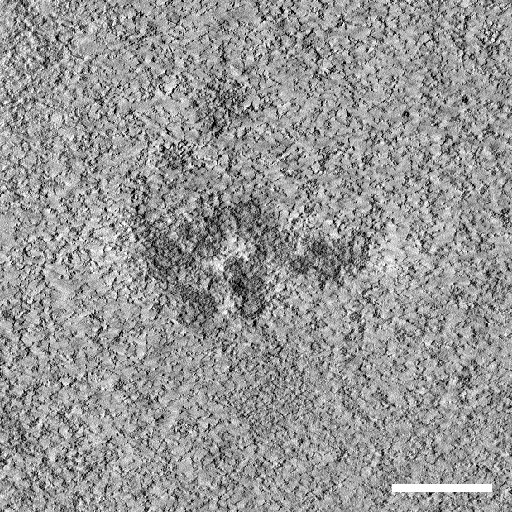

Supplement: Supplementary file 10 — Source data Fig. 5 [file 44318_2025_652_MOESM10_ESM.zip › Figure 5/5A/5A_ETi_stack/modv0261.jpg]

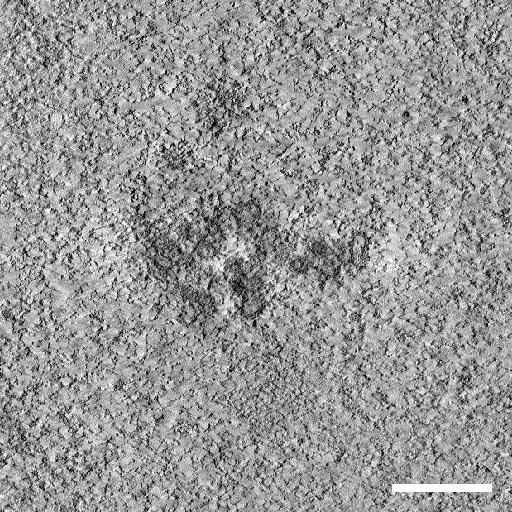

Supplement: Supplementary file 10 — Source data Fig. 5 [file 44318_2025_652_MOESM10_ESM.zip › Figure 5/5A/5A_ETi_stack/modv0260.jpg]

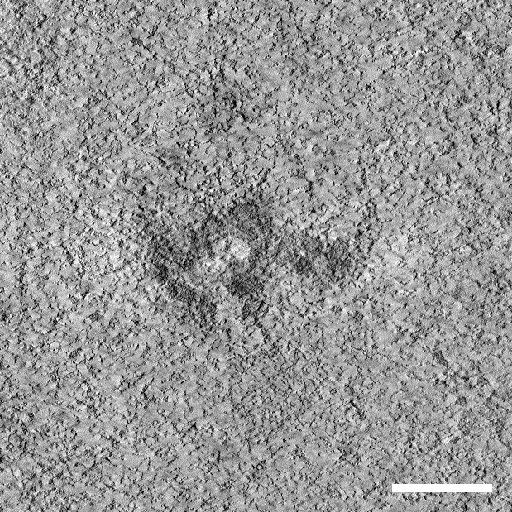

Supplement: Supplementary file 10 — Source data Fig. 5 [file 44318_2025_652_MOESM10_ESM.zip › Figure 5/5A/5A_ETi_stack/modv0274.jpg]

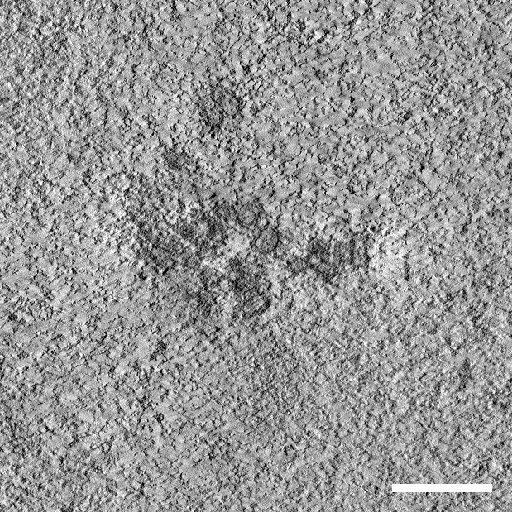

Supplement: Supplementary file 10 — Source data Fig. 5 [file 44318_2025_652_MOESM10_ESM.zip › Figure 5/5A/5A_ETi_stack/modv0248.jpg]
