# Supplementary material for: Lenacapavir-induced capsid damage uncovers HIV-1 genomes emanating from nuclear speckles
Source: EMBO J. 2025 Dec 1;45(2):449–70. doi: 10.1038/s44318-025-00652-5 (PMC12811339; doi:10.1038/s44318-025-00652-5)
Supplement: Supplementary file 7 — Source data Fig. 2 [file 44318_2025_652_MOESM7_ESM.zip › Figure 2/2A/channel information.rtf]

channel 1: LMNAchannel 2: IN.eGFPchannel 3: CPSF6
