# Supplementary figures and images for: Lenacapavir-induced capsid damage uncovers HIV-1 genomes emanating from nuclear speckles (part 3 of 3)
Source: EMBO J. 2025 Dec 1;45(2):449–70. doi: 10.1038/s44318-025-00652-5 (PMC12811339; doi:10.1038/s44318-025-00652-5)

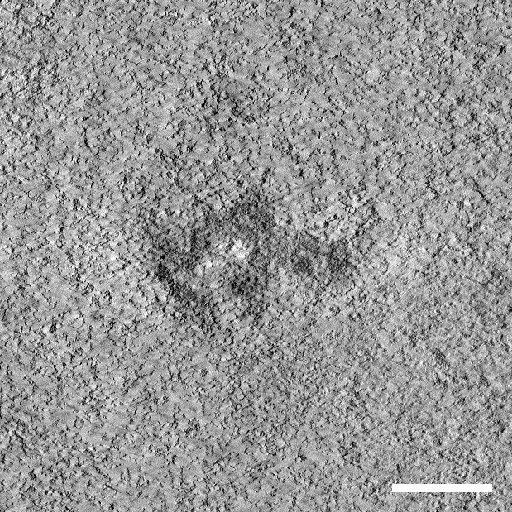

Supplement: Supplementary file 10 — Source data Fig. 5 [file 44318_2025_652_MOESM10_ESM.zip › Figure 5/5A/5A_ETi_stack/modv0281.jpg]

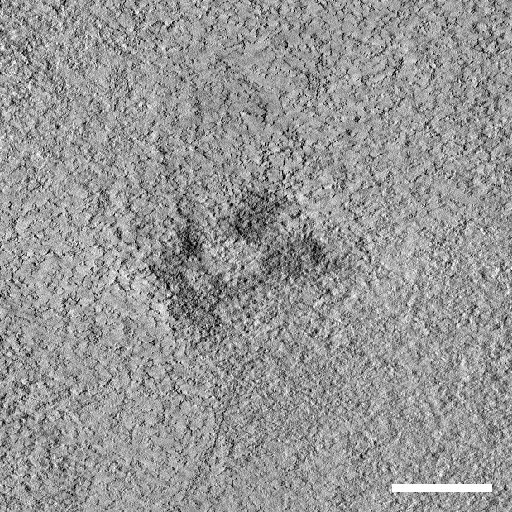

Supplement: Supplementary file 10 — Source data Fig. 5 [file 44318_2025_652_MOESM10_ESM.zip › Figure 5/5A/5A_ETi_stack/modv0322.jpg]

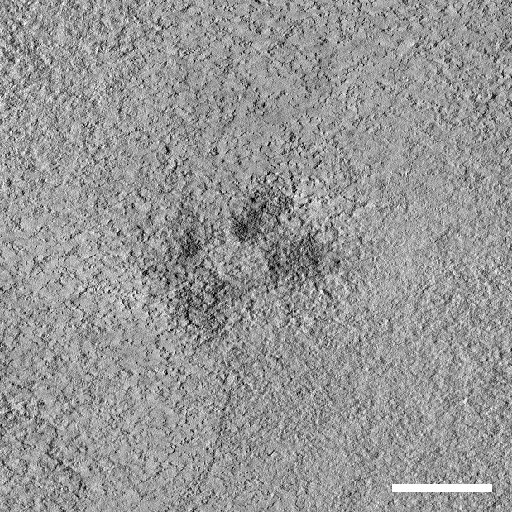

Supplement: Supplementary file 10 — Source data Fig. 5 [file 44318_2025_652_MOESM10_ESM.zip › Figure 5/5A/5A_ETi_stack/modv0336.jpg]

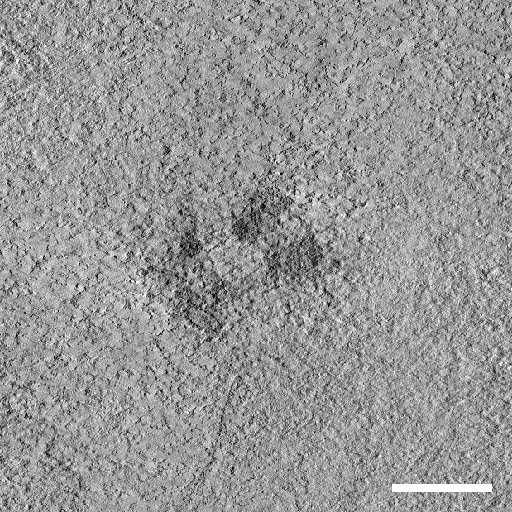

Supplement: Supplementary file 10 — Source data Fig. 5 [file 44318_2025_652_MOESM10_ESM.zip › Figure 5/5A/5A_ETi_stack/modv0332.jpg]

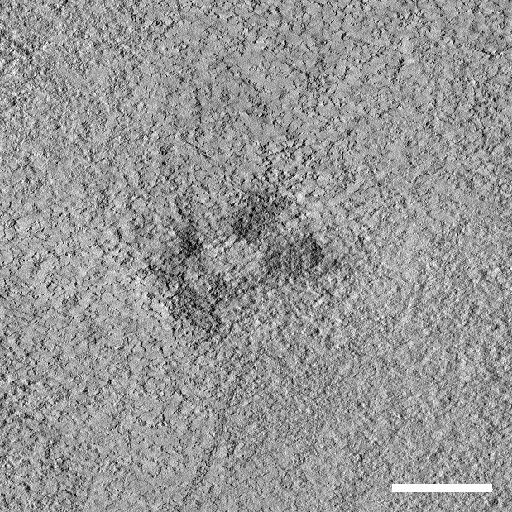

Supplement: Supplementary file 10 — Source data Fig. 5 [file 44318_2025_652_MOESM10_ESM.zip › Figure 5/5A/5A_ETi_stack/modv0326.jpg]

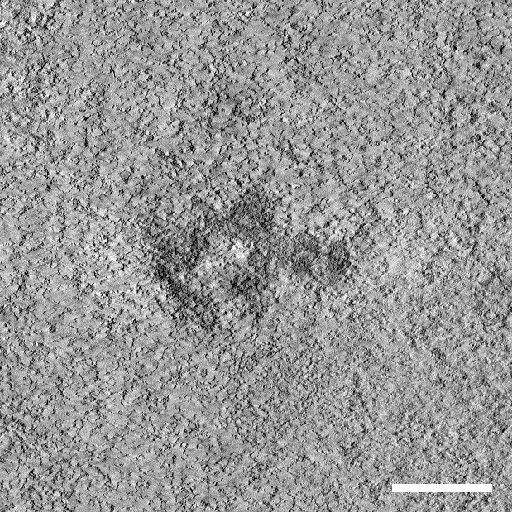

Supplement: Supplementary file 10 — Source data Fig. 5 [file 44318_2025_652_MOESM10_ESM.zip › Figure 5/5A/5A_ETi_stack/modv0285.jpg]

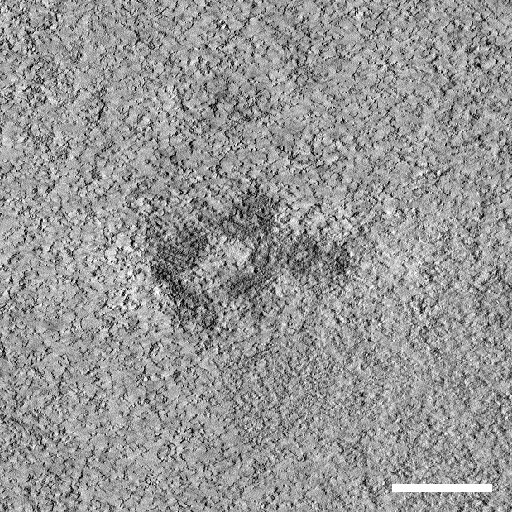

Supplement: Supplementary file 10 — Source data Fig. 5 [file 44318_2025_652_MOESM10_ESM.zip › Figure 5/5A/5A_ETi_stack/modv0291.jpg]

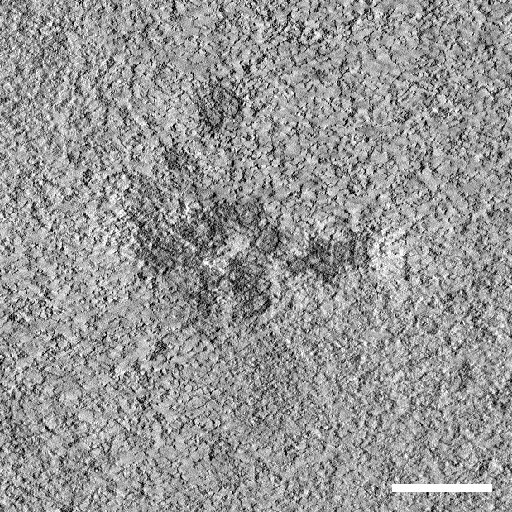

Supplement: Supplementary file 10 — Source data Fig. 5 [file 44318_2025_652_MOESM10_ESM.zip › Figure 5/5A/5A_ETi_stack/modv0246.jpg]

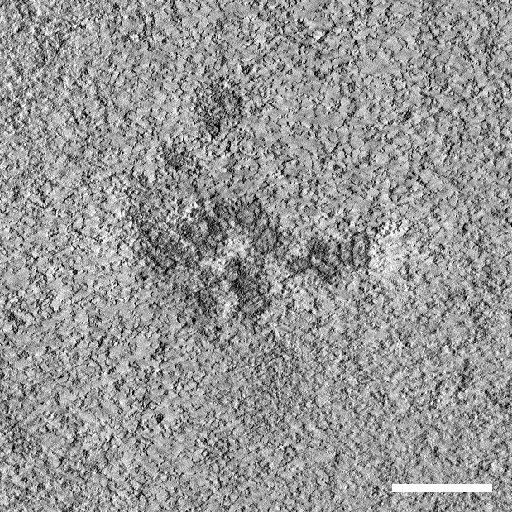

Supplement: Supplementary file 10 — Source data Fig. 5 [file 44318_2025_652_MOESM10_ESM.zip › Figure 5/5A/5A_ETi_stack/modv0252.jpg]

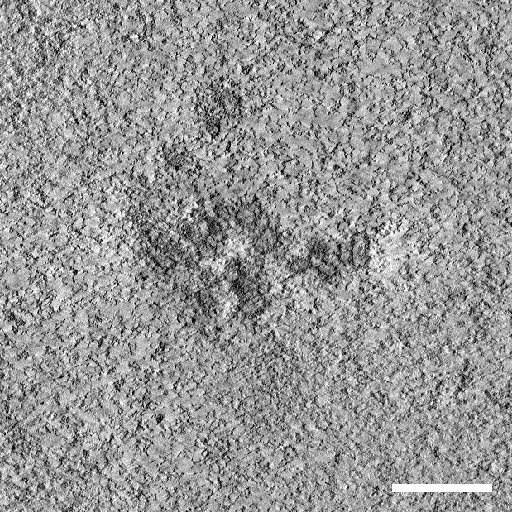

Supplement: Supplementary file 10 — Source data Fig. 5 [file 44318_2025_652_MOESM10_ESM.zip › Figure 5/5A/5A_ETi_stack/modv0253.jpg]

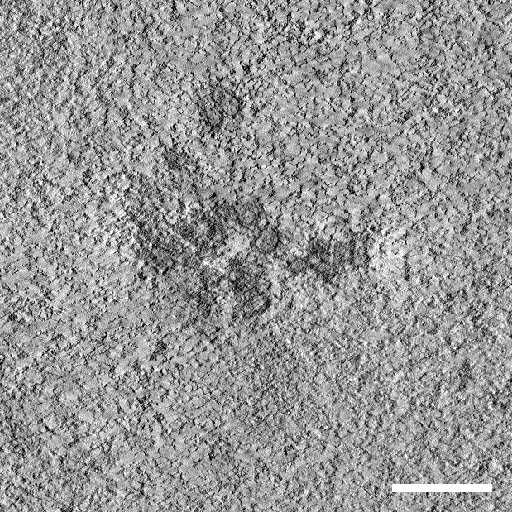

Supplement: Supplementary file 10 — Source data Fig. 5 [file 44318_2025_652_MOESM10_ESM.zip › Figure 5/5A/5A_ETi_stack/modv0247.jpg]

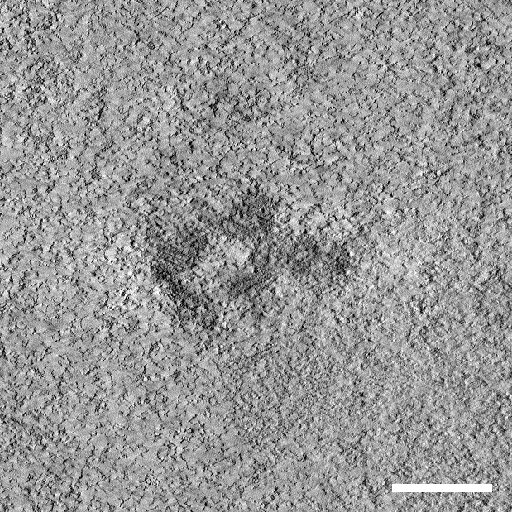

Supplement: Supplementary file 10 — Source data Fig. 5 [file 44318_2025_652_MOESM10_ESM.zip › Figure 5/5A/5A_ETi_stack/modv0290.jpg]

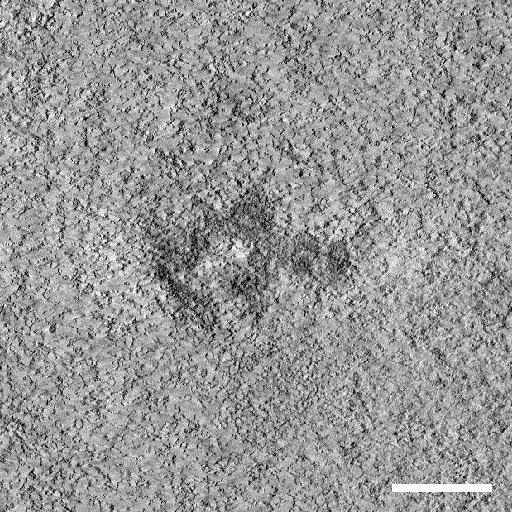

Supplement: Supplementary file 10 — Source data Fig. 5 [file 44318_2025_652_MOESM10_ESM.zip › Figure 5/5A/5A_ETi_stack/modv0284.jpg]

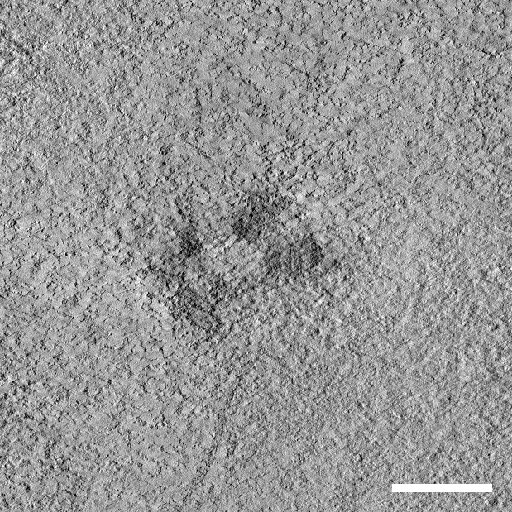

Supplement: Supplementary file 10 — Source data Fig. 5 [file 44318_2025_652_MOESM10_ESM.zip › Figure 5/5A/5A_ETi_stack/modv0327.jpg]

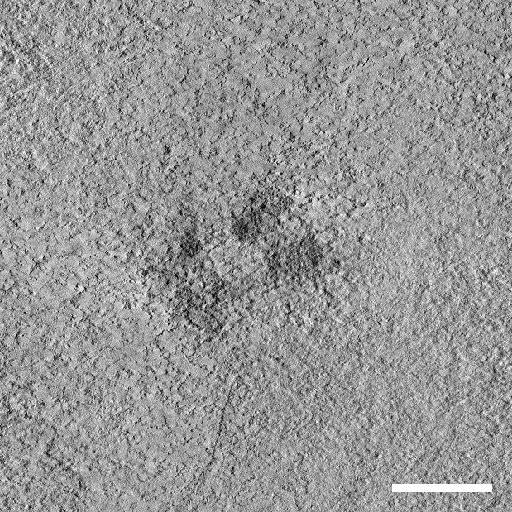

Supplement: Supplementary file 10 — Source data Fig. 5 [file 44318_2025_652_MOESM10_ESM.zip › Figure 5/5A/5A_ETi_stack/modv0333.jpg]

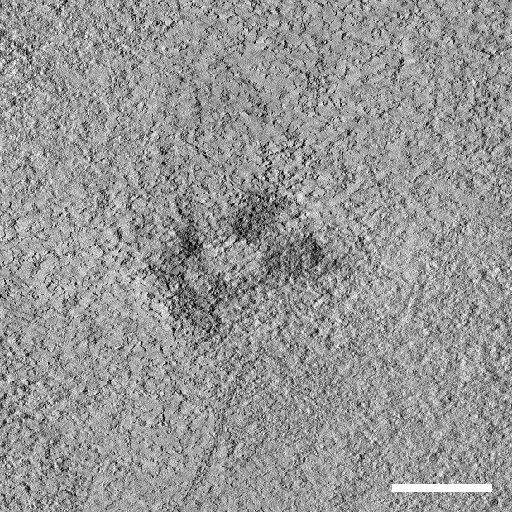

Supplement: Supplementary file 10 — Source data Fig. 5 [file 44318_2025_652_MOESM10_ESM.zip › Figure 5/5A/5A_ETi_stack/modv0325.jpg]

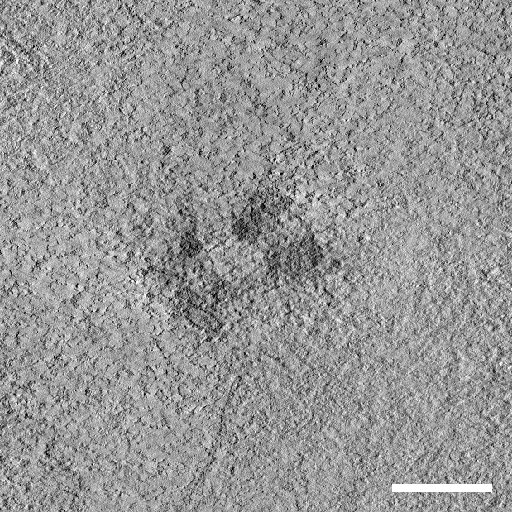

Supplement: Supplementary file 10 — Source data Fig. 5 [file 44318_2025_652_MOESM10_ESM.zip › Figure 5/5A/5A_ETi_stack/modv0331.jpg]

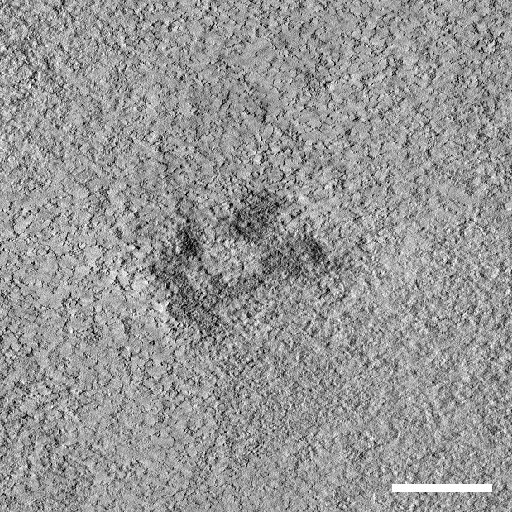

Supplement: Supplementary file 10 — Source data Fig. 5 [file 44318_2025_652_MOESM10_ESM.zip › Figure 5/5A/5A_ETi_stack/modv0319.jpg]

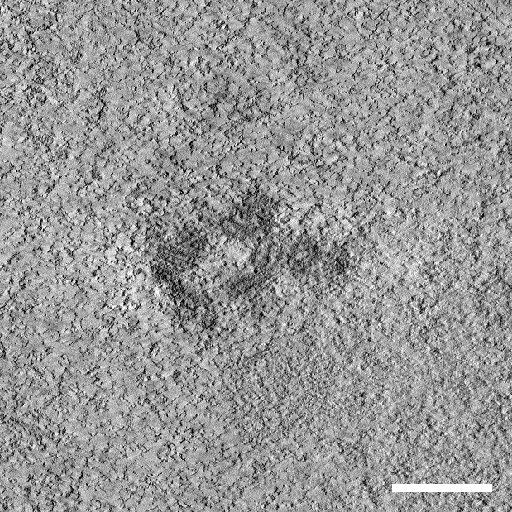

Supplement: Supplementary file 10 — Source data Fig. 5 [file 44318_2025_652_MOESM10_ESM.zip › Figure 5/5A/5A_ETi_stack/modv0292.jpg]

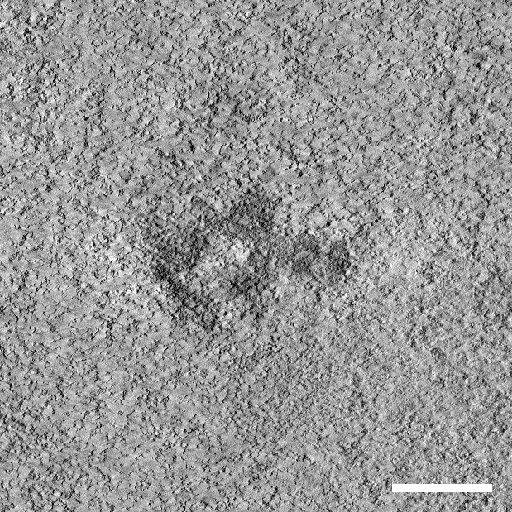

Supplement: Supplementary file 10 — Source data Fig. 5 [file 44318_2025_652_MOESM10_ESM.zip › Figure 5/5A/5A_ETi_stack/modv0286.jpg]

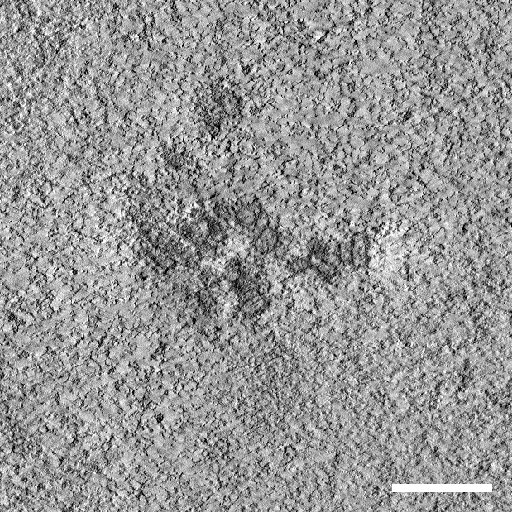

Supplement: Supplementary file 10 — Source data Fig. 5 [file 44318_2025_652_MOESM10_ESM.zip › Figure 5/5A/5A_ETi_stack/modv0251.jpg]

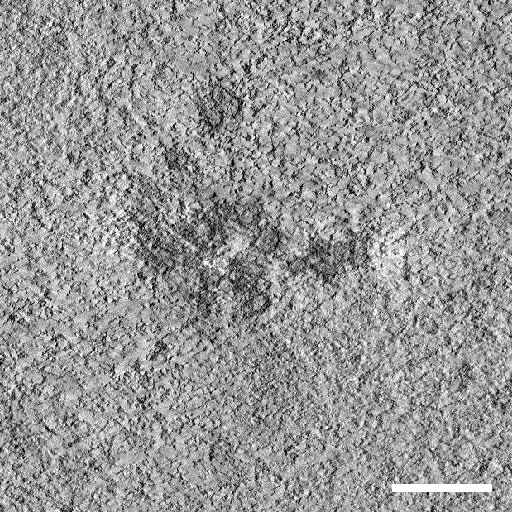

Supplement: Supplementary file 10 — Source data Fig. 5 [file 44318_2025_652_MOESM10_ESM.zip › Figure 5/5A/5A_ETi_stack/modv0245.jpg]

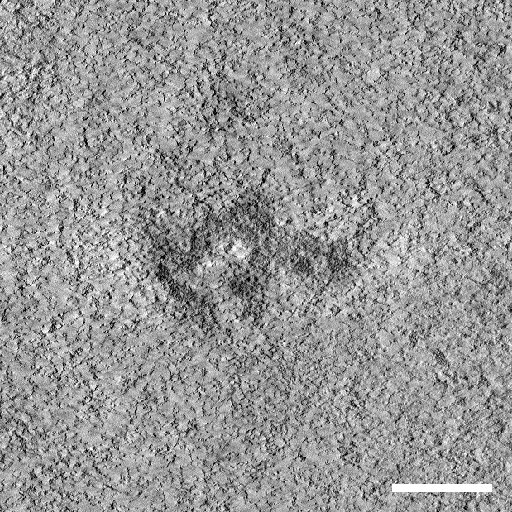

Supplement: Supplementary file 10 — Source data Fig. 5 [file 44318_2025_652_MOESM10_ESM.zip › Figure 5/5A/5A_ETi_stack/modv0279.jpg]

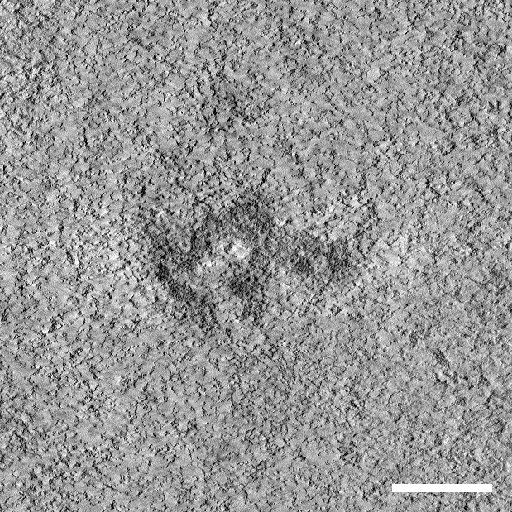

Supplement: Supplementary file 10 — Source data Fig. 5 [file 44318_2025_652_MOESM10_ESM.zip › Figure 5/5A/5A_ETi_stack/modv0278.jpg]

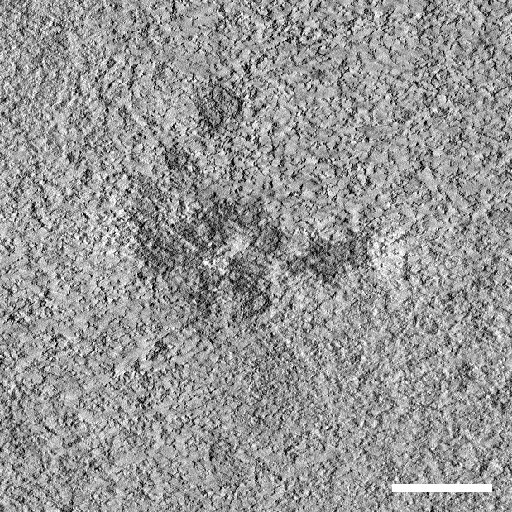

Supplement: Supplementary file 10 — Source data Fig. 5 [file 44318_2025_652_MOESM10_ESM.zip › Figure 5/5A/5A_ETi_stack/modv0244.jpg]

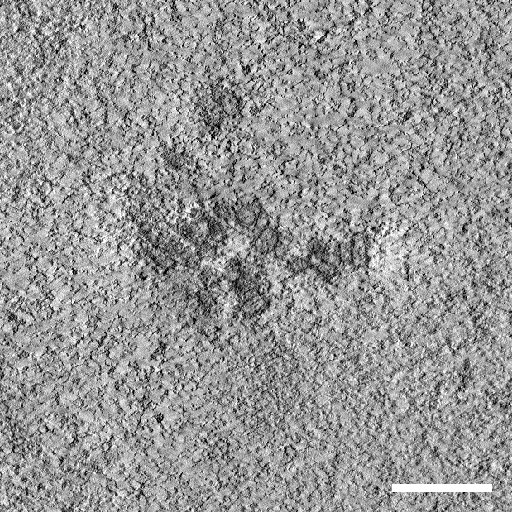

Supplement: Supplementary file 10 — Source data Fig. 5 [file 44318_2025_652_MOESM10_ESM.zip › Figure 5/5A/5A_ETi_stack/modv0250.jpg]

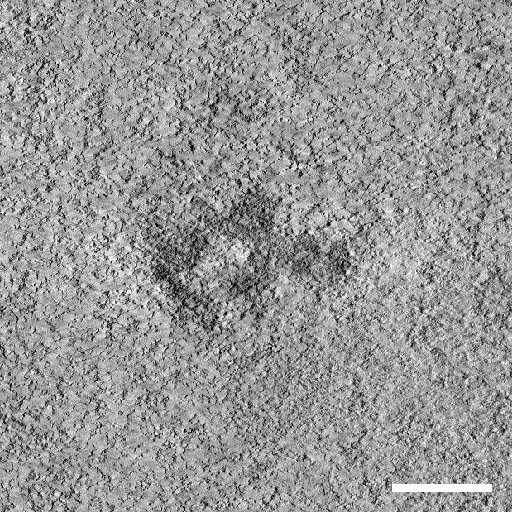

Supplement: Supplementary file 10 — Source data Fig. 5 [file 44318_2025_652_MOESM10_ESM.zip › Figure 5/5A/5A_ETi_stack/modv0287.jpg]

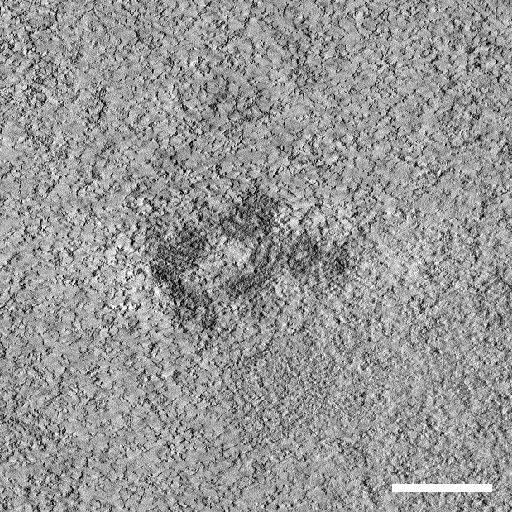

Supplement: Supplementary file 10 — Source data Fig. 5 [file 44318_2025_652_MOESM10_ESM.zip › Figure 5/5A/5A_ETi_stack/modv0293.jpg]

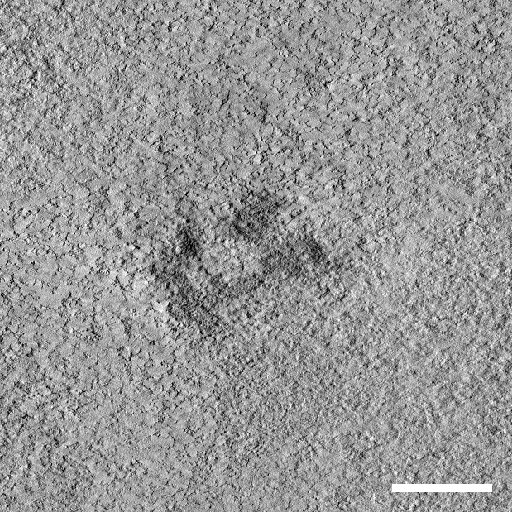

Supplement: Supplementary file 10 — Source data Fig. 5 [file 44318_2025_652_MOESM10_ESM.zip › Figure 5/5A/5A_ETi_stack/modv0318.jpg]

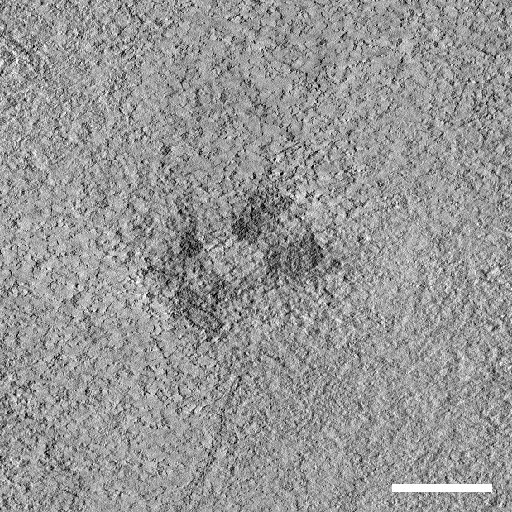

Supplement: Supplementary file 10 — Source data Fig. 5 [file 44318_2025_652_MOESM10_ESM.zip › Figure 5/5A/5A_ETi_stack/modv0330.jpg]

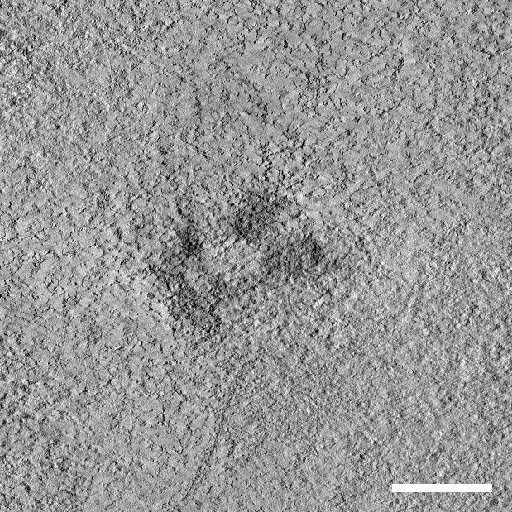

Supplement: Supplementary file 10 — Source data Fig. 5 [file 44318_2025_652_MOESM10_ESM.zip › Figure 5/5A/5A_ETi_stack/modv0324.jpg]

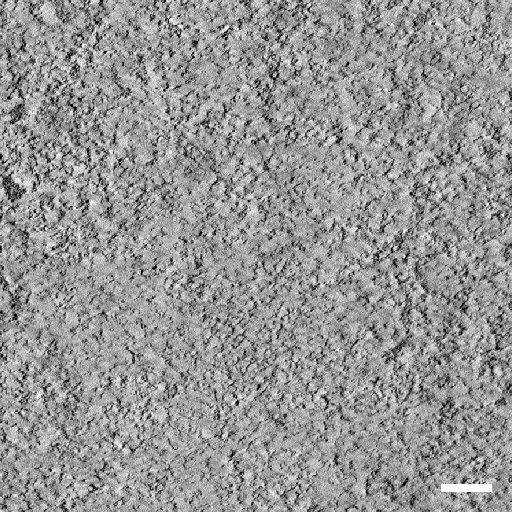

Supplement: Supplementary file 10 — Source data Fig. 5 [file 44318_2025_652_MOESM10_ESM.zip › Figure 5/5A/5A_ETii_stack/modv0103.jpg]

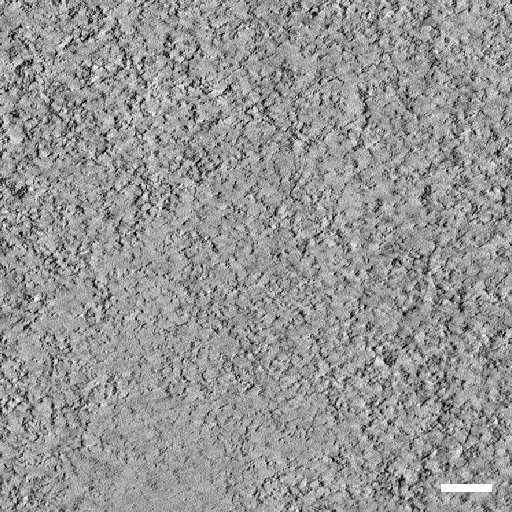

Supplement: Supplementary file 10 — Source data Fig. 5 [file 44318_2025_652_MOESM10_ESM.zip › Figure 5/5A/5A_ETii_stack/modv0117.jpg]

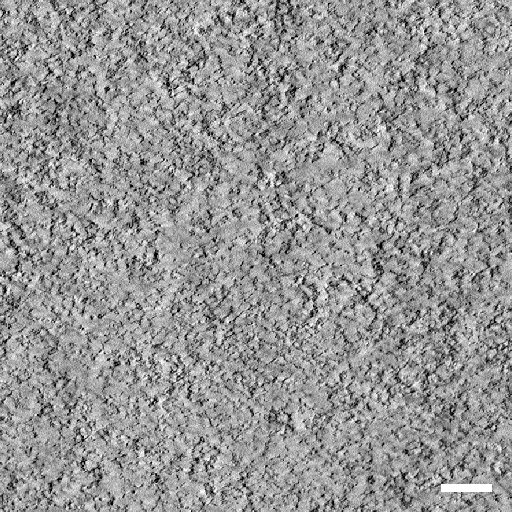

Supplement: Supplementary file 10 — Source data Fig. 5 [file 44318_2025_652_MOESM10_ESM.zip › Figure 5/5A/5A_ETii_stack/modv0088.jpg]

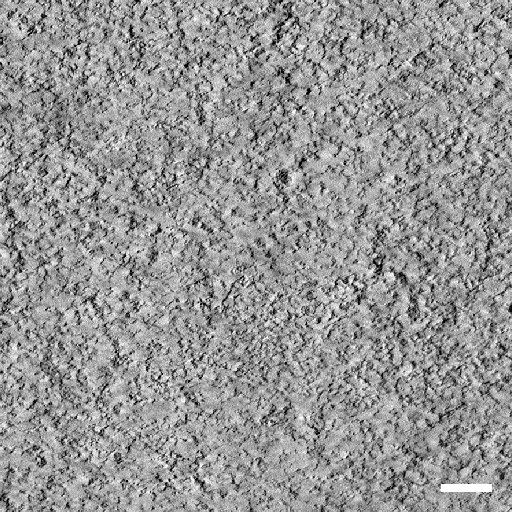

Supplement: Supplementary file 10 — Source data Fig. 5 [file 44318_2025_652_MOESM10_ESM.zip › Figure 5/5A/5A_ETii_stack/modv0077.jpg]

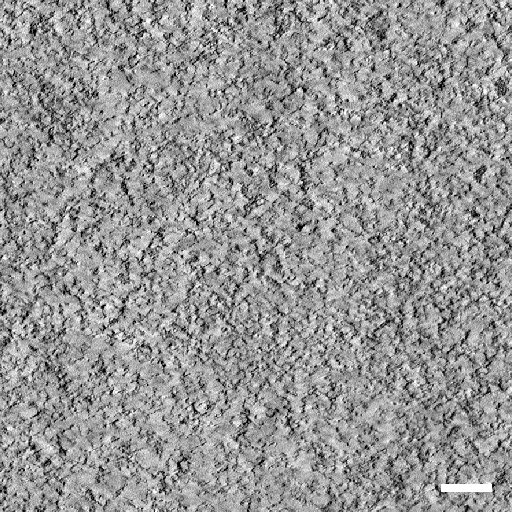

Supplement: Supplementary file 10 — Source data Fig. 5 [file 44318_2025_652_MOESM10_ESM.zip › Figure 5/5A/5A_ETii_stack/modv0063.jpg]

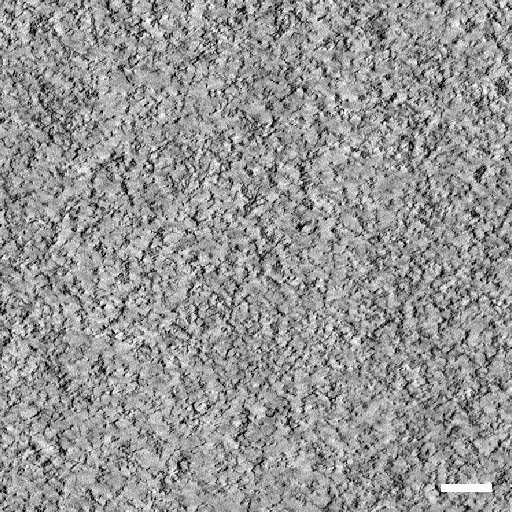

Supplement: Supplementary file 10 — Source data Fig. 5 [file 44318_2025_652_MOESM10_ESM.zip › Figure 5/5A/5A_ETii_stack/modv0062.jpg]

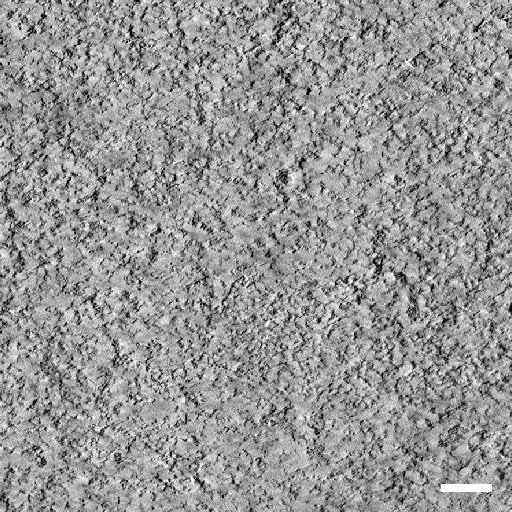

Supplement: Supplementary file 10 — Source data Fig. 5 [file 44318_2025_652_MOESM10_ESM.zip › Figure 5/5A/5A_ETii_stack/modv0076.jpg]

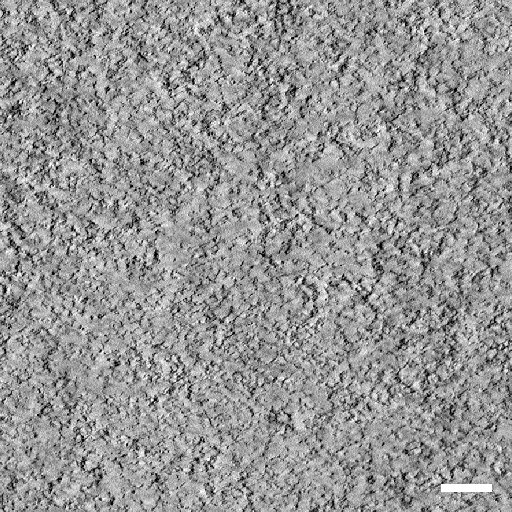

Supplement: Supplementary file 10 — Source data Fig. 5 [file 44318_2025_652_MOESM10_ESM.zip › Figure 5/5A/5A_ETii_stack/modv0089.jpg]

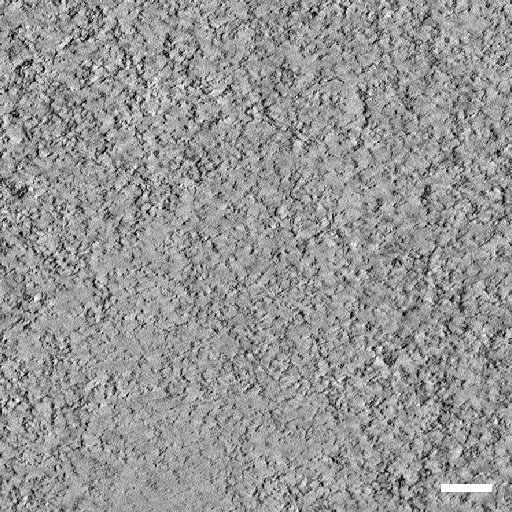

Supplement: Supplementary file 10 — Source data Fig. 5 [file 44318_2025_652_MOESM10_ESM.zip › Figure 5/5A/5A_ETii_stack/modv0116.jpg]

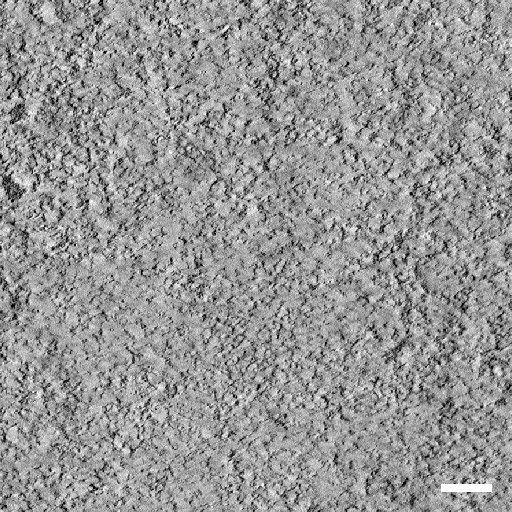

Supplement: Supplementary file 10 — Source data Fig. 5 [file 44318_2025_652_MOESM10_ESM.zip › Figure 5/5A/5A_ETii_stack/modv0102.jpg]

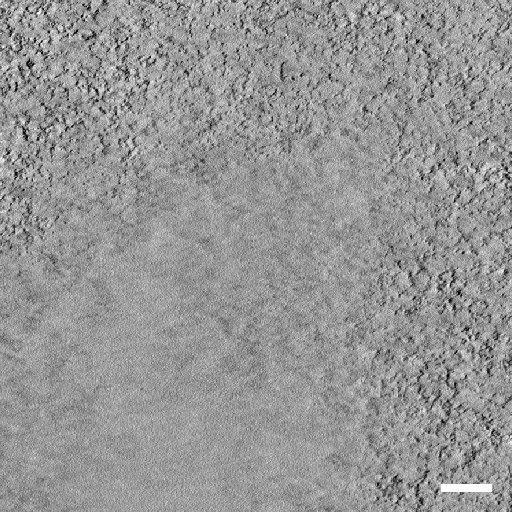

Supplement: Supplementary file 10 — Source data Fig. 5 [file 44318_2025_652_MOESM10_ESM.zip › Figure 5/5A/5A_ETii_stack/modv0128.jpg]

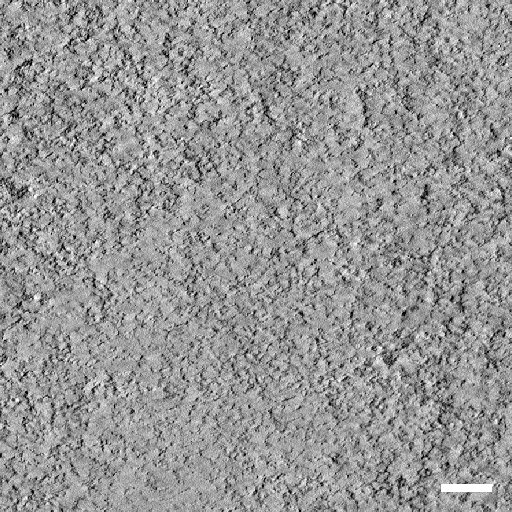

Supplement: Supplementary file 10 — Source data Fig. 5 [file 44318_2025_652_MOESM10_ESM.zip › Figure 5/5A/5A_ETii_stack/modv0114.jpg]

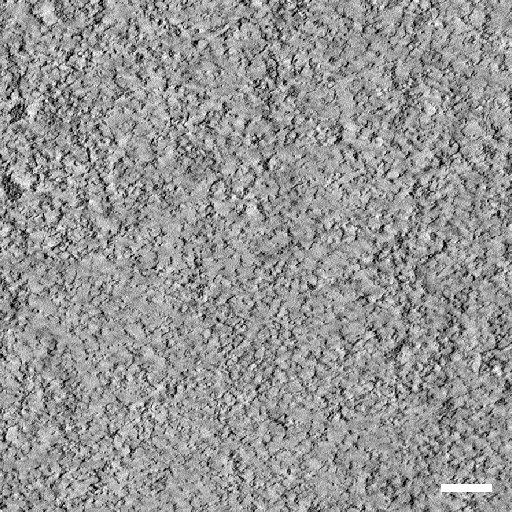

Supplement: Supplementary file 10 — Source data Fig. 5 [file 44318_2025_652_MOESM10_ESM.zip › Figure 5/5A/5A_ETii_stack/modv0100.jpg]

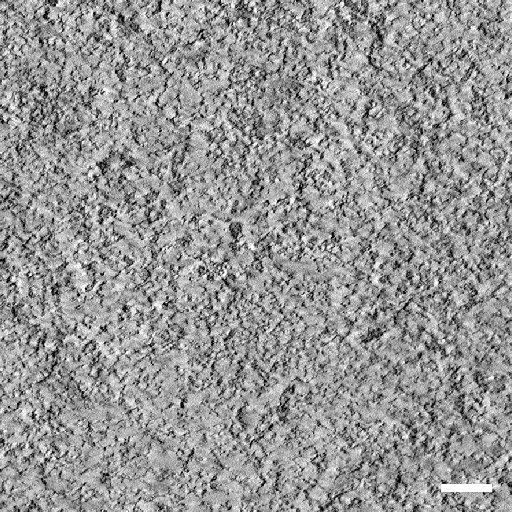

Supplement: Supplementary file 10 — Source data Fig. 5 [file 44318_2025_652_MOESM10_ESM.zip › Figure 5/5A/5A_ETii_stack/modv0048.jpg]

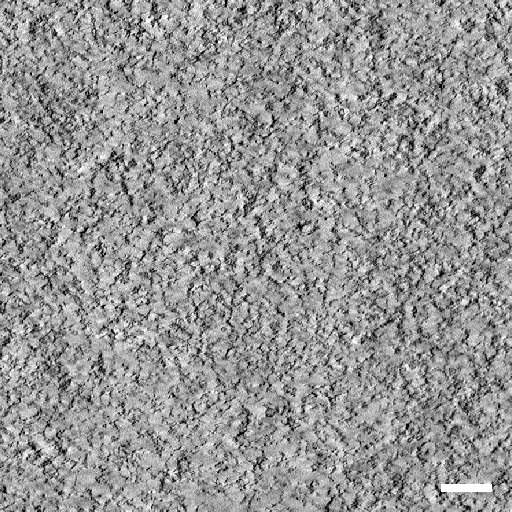

Supplement: Supplementary file 10 — Source data Fig. 5 [file 44318_2025_652_MOESM10_ESM.zip › Figure 5/5A/5A_ETii_stack/modv0060.jpg]

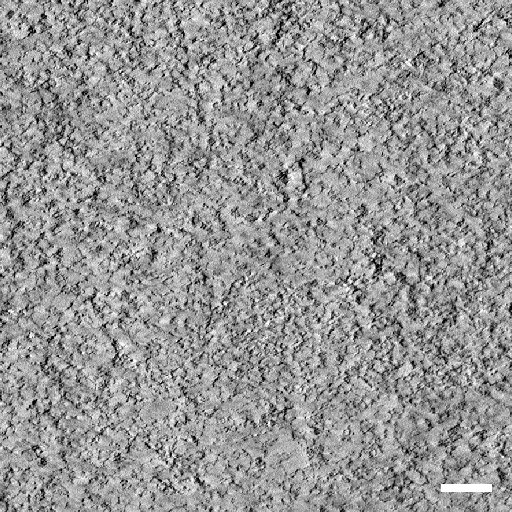

Supplement: Supplementary file 10 — Source data Fig. 5 [file 44318_2025_652_MOESM10_ESM.zip › Figure 5/5A/5A_ETii_stack/modv0074.jpg]

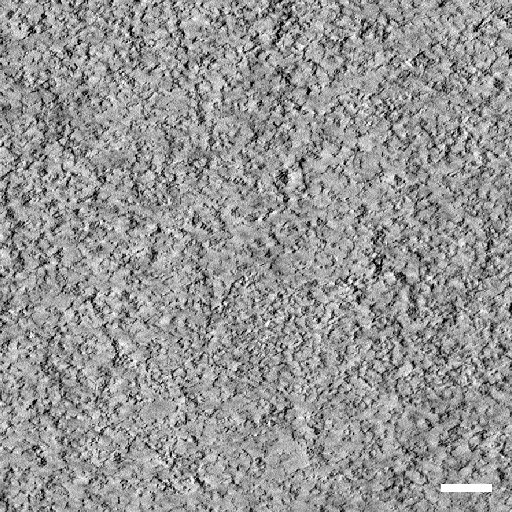

Supplement: Supplementary file 10 — Source data Fig. 5 [file 44318_2025_652_MOESM10_ESM.zip › Figure 5/5A/5A_ETii_stack/modv0075.jpg]

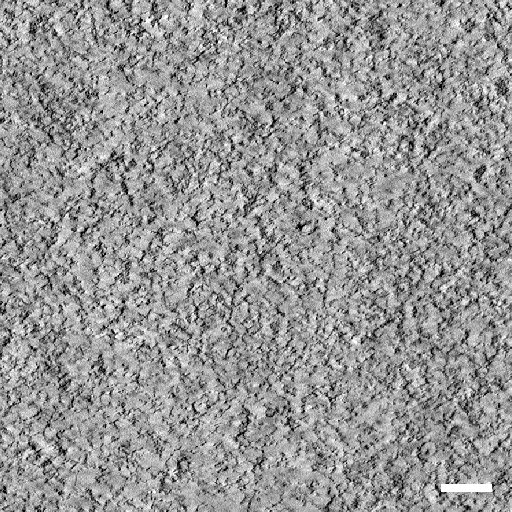

Supplement: Supplementary file 10 — Source data Fig. 5 [file 44318_2025_652_MOESM10_ESM.zip › Figure 5/5A/5A_ETii_stack/modv0061.jpg]

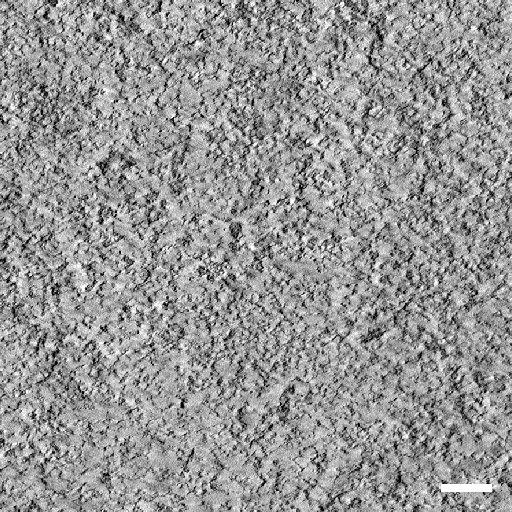

Supplement: Supplementary file 10 — Source data Fig. 5 [file 44318_2025_652_MOESM10_ESM.zip › Figure 5/5A/5A_ETii_stack/modv0049.jpg]

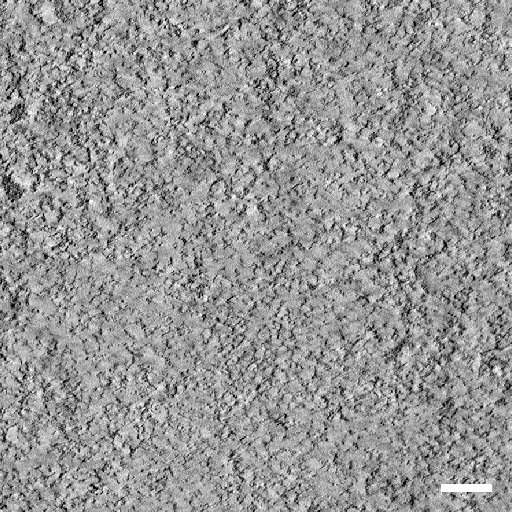

Supplement: Supplementary file 10 — Source data Fig. 5 [file 44318_2025_652_MOESM10_ESM.zip › Figure 5/5A/5A_ETii_stack/modv0101.jpg]

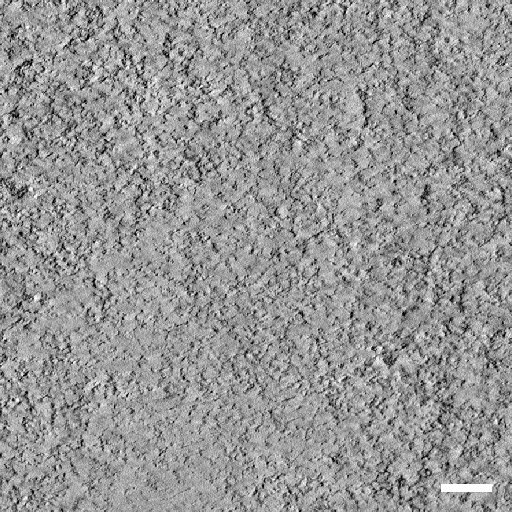

Supplement: Supplementary file 10 — Source data Fig. 5 [file 44318_2025_652_MOESM10_ESM.zip › Figure 5/5A/5A_ETii_stack/modv0115.jpg]

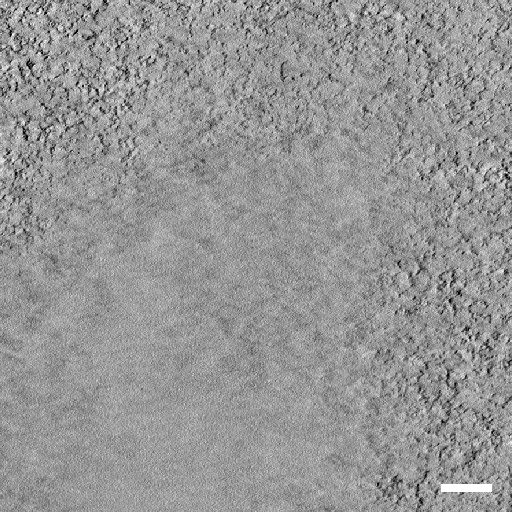

Supplement: Supplementary file 10 — Source data Fig. 5 [file 44318_2025_652_MOESM10_ESM.zip › Figure 5/5A/5A_ETii_stack/modv0129.jpg]

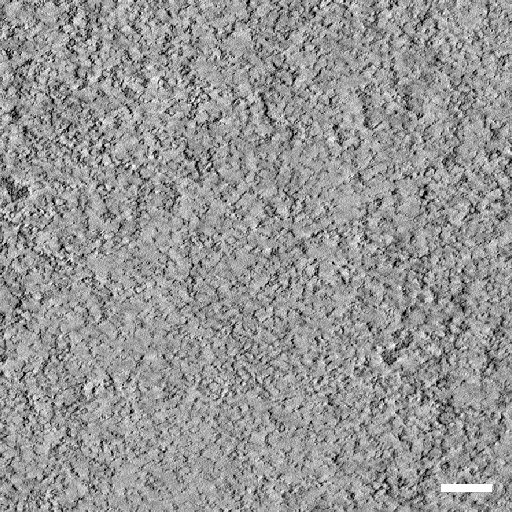

Supplement: Supplementary file 10 — Source data Fig. 5 [file 44318_2025_652_MOESM10_ESM.zip › Figure 5/5A/5A_ETii_stack/modv0111.jpg]

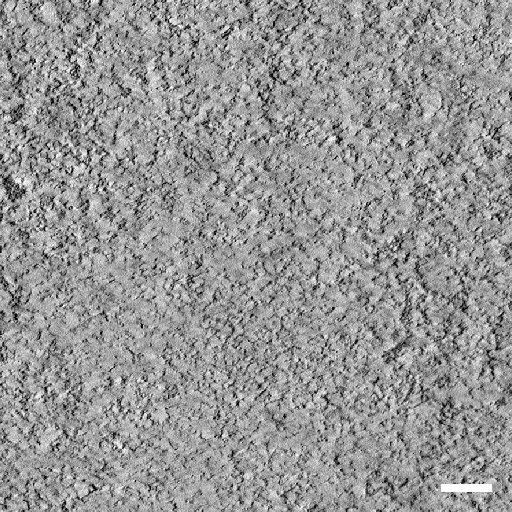

Supplement: Supplementary file 10 — Source data Fig. 5 [file 44318_2025_652_MOESM10_ESM.zip › Figure 5/5A/5A_ETii_stack/modv0105.jpg]

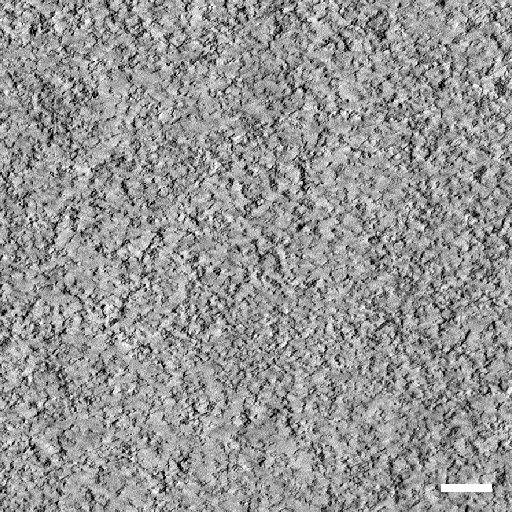

Supplement: Supplementary file 10 — Source data Fig. 5 [file 44318_2025_652_MOESM10_ESM.zip › Figure 5/5A/5A_ETii_stack/modv0065.jpg]

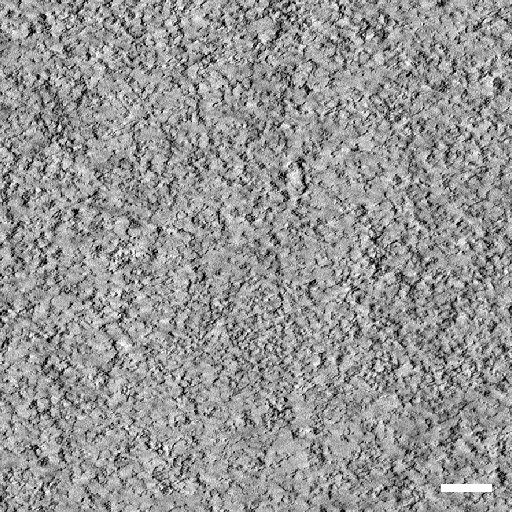

Supplement: Supplementary file 10 — Source data Fig. 5 [file 44318_2025_652_MOESM10_ESM.zip › Figure 5/5A/5A_ETii_stack/modv0071.jpg]

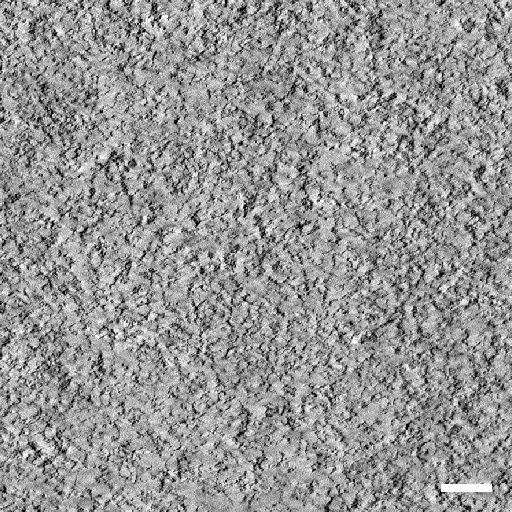

Supplement: Supplementary file 10 — Source data Fig. 5 [file 44318_2025_652_MOESM10_ESM.zip › Figure 5/5A/5A_ETii_stack/modv0059.jpg]

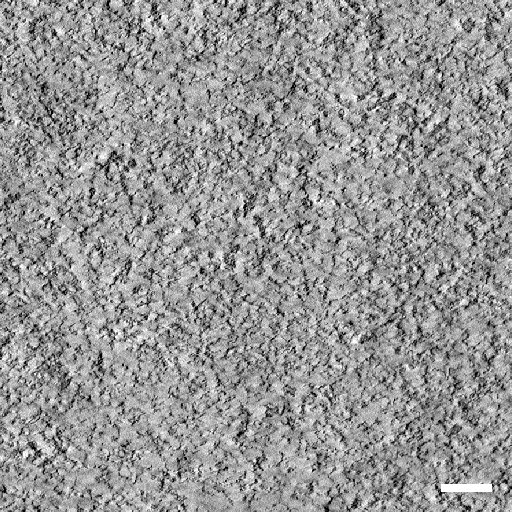

Supplement: Supplementary file 10 — Source data Fig. 5 [file 44318_2025_652_MOESM10_ESM.zip › Figure 5/5A/5A_ETii_stack/modv0058.jpg]

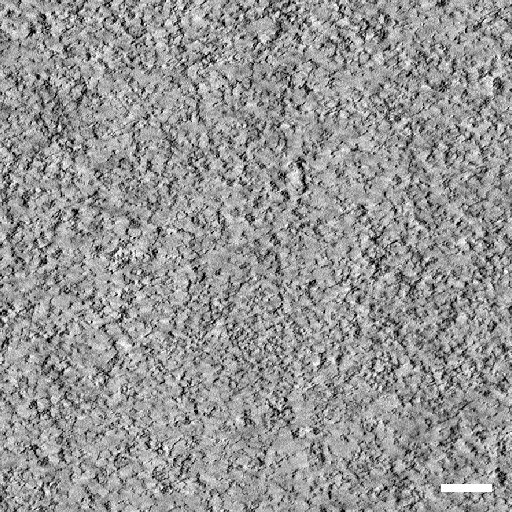

Supplement: Supplementary file 10 — Source data Fig. 5 [file 44318_2025_652_MOESM10_ESM.zip › Figure 5/5A/5A_ETii_stack/modv0070.jpg]

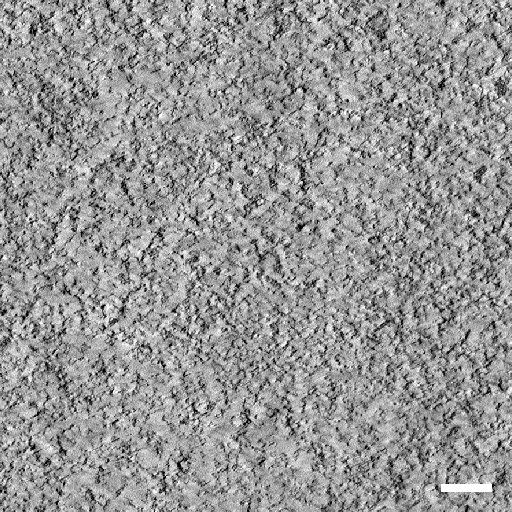

Supplement: Supplementary file 10 — Source data Fig. 5 [file 44318_2025_652_MOESM10_ESM.zip › Figure 5/5A/5A_ETii_stack/modv0064.jpg]

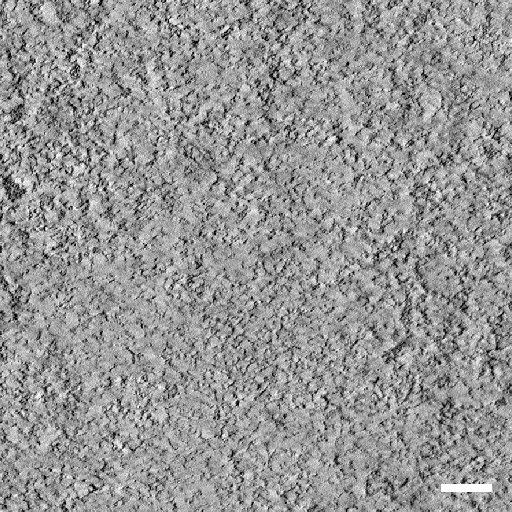

Supplement: Supplementary file 10 — Source data Fig. 5 [file 44318_2025_652_MOESM10_ESM.zip › Figure 5/5A/5A_ETii_stack/modv0104.jpg]

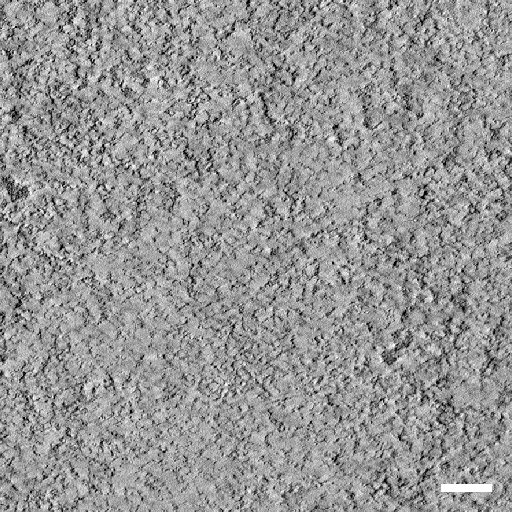

Supplement: Supplementary file 10 — Source data Fig. 5 [file 44318_2025_652_MOESM10_ESM.zip › Figure 5/5A/5A_ETii_stack/modv0110.jpg]

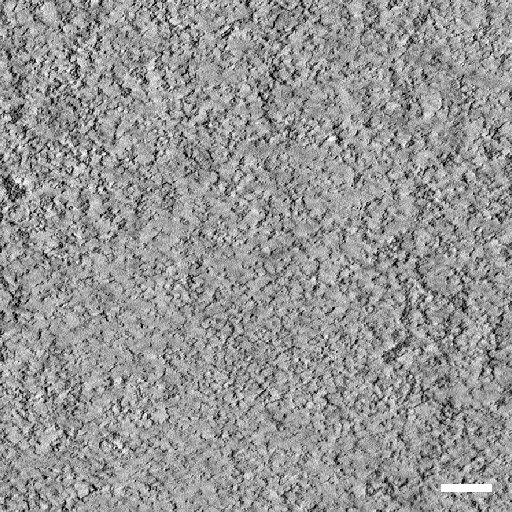

Supplement: Supplementary file 10 — Source data Fig. 5 [file 44318_2025_652_MOESM10_ESM.zip › Figure 5/5A/5A_ETii_stack/modv0106.jpg]

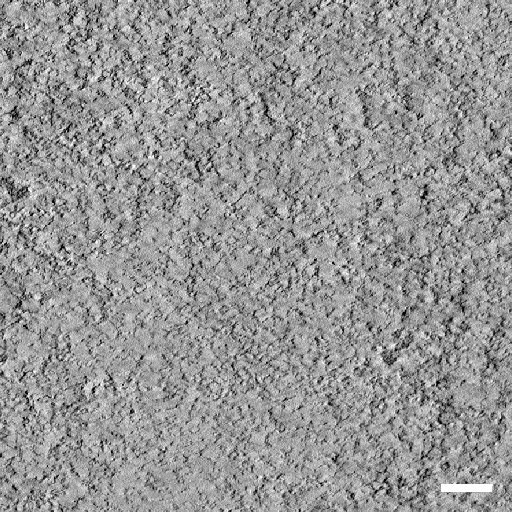

Supplement: Supplementary file 10 — Source data Fig. 5 [file 44318_2025_652_MOESM10_ESM.zip › Figure 5/5A/5A_ETii_stack/modv0112.jpg]

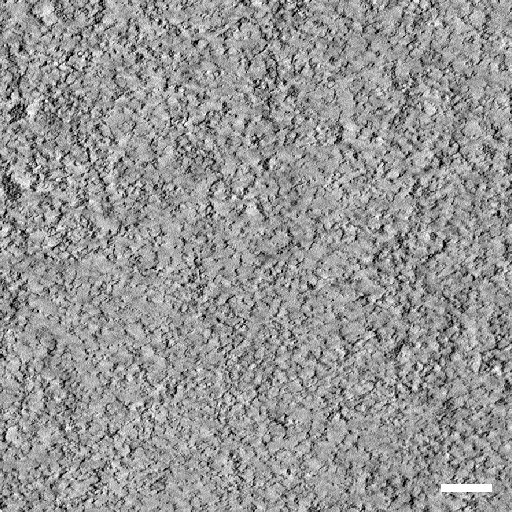

Supplement: Supplementary file 10 — Source data Fig. 5 [file 44318_2025_652_MOESM10_ESM.zip › Figure 5/5A/5A_ETii_stack/modv0099.jpg]

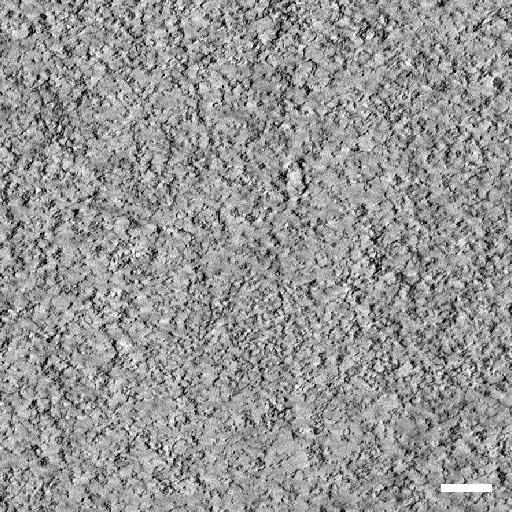

Supplement: Supplementary file 10 — Source data Fig. 5 [file 44318_2025_652_MOESM10_ESM.zip › Figure 5/5A/5A_ETii_stack/modv0072.jpg]

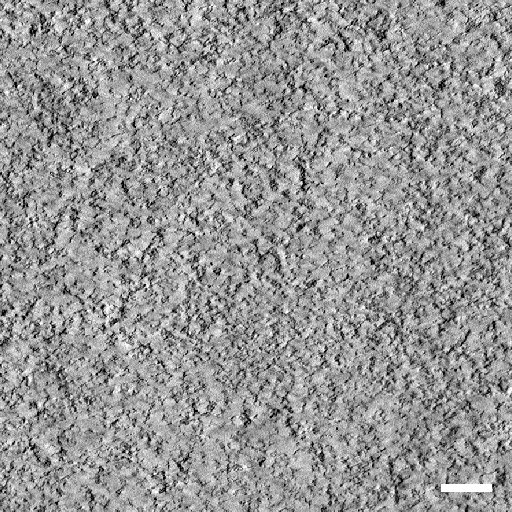

Supplement: Supplementary file 10 — Source data Fig. 5 [file 44318_2025_652_MOESM10_ESM.zip › Figure 5/5A/5A_ETii_stack/modv0066.jpg]

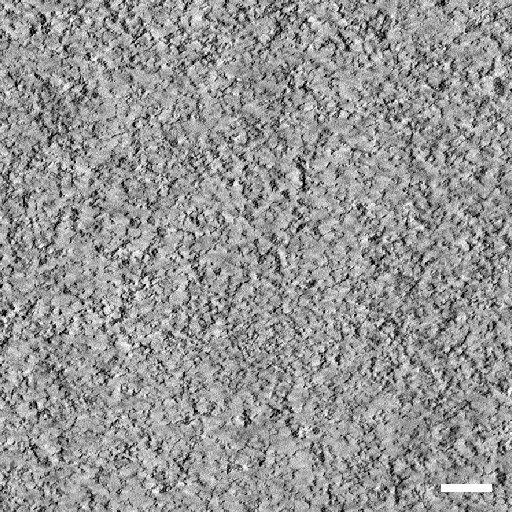

Supplement: Supplementary file 10 — Source data Fig. 5 [file 44318_2025_652_MOESM10_ESM.zip › Figure 5/5A/5A_ETii_stack/modv0067.jpg]

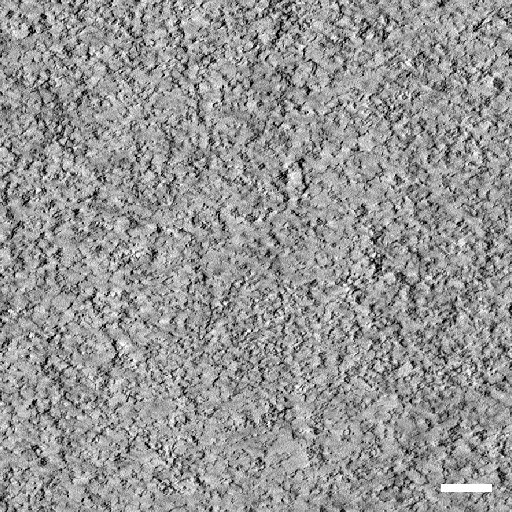

Supplement: Supplementary file 10 — Source data Fig. 5 [file 44318_2025_652_MOESM10_ESM.zip › Figure 5/5A/5A_ETii_stack/modv0073.jpg]

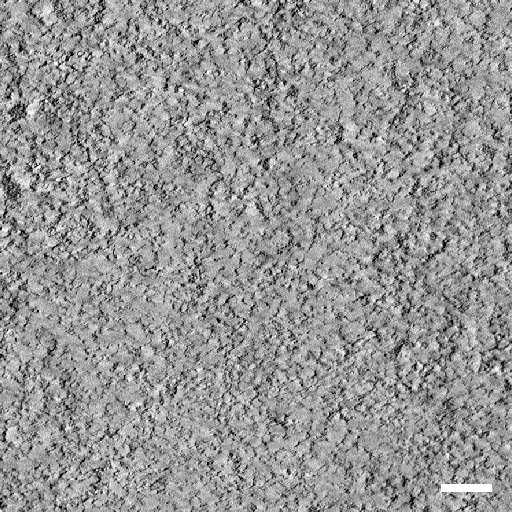

Supplement: Supplementary file 10 — Source data Fig. 5 [file 44318_2025_652_MOESM10_ESM.zip › Figure 5/5A/5A_ETii_stack/modv0098.jpg]

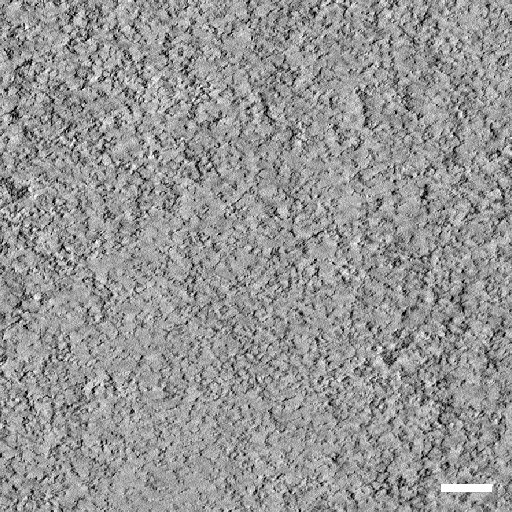

Supplement: Supplementary file 10 — Source data Fig. 5 [file 44318_2025_652_MOESM10_ESM.zip › Figure 5/5A/5A_ETii_stack/modv0113.jpg]

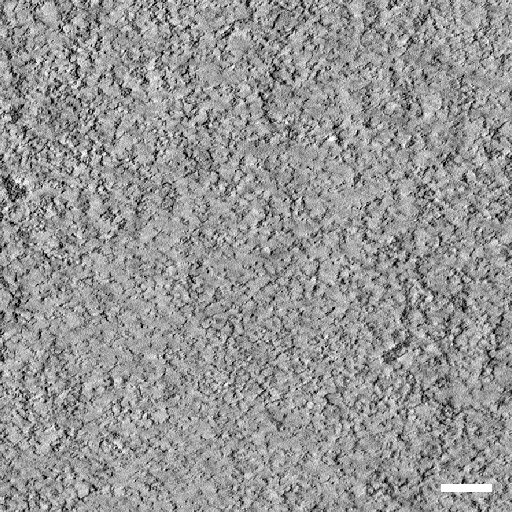

Supplement: Supplementary file 10 — Source data Fig. 5 [file 44318_2025_652_MOESM10_ESM.zip › Figure 5/5A/5A_ETii_stack/modv0107.jpg]

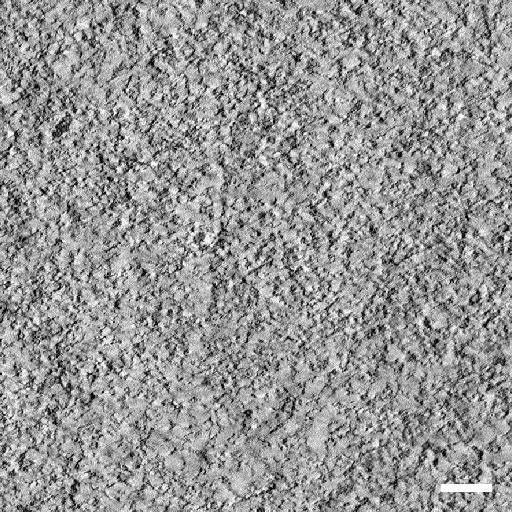

Supplement: Supplementary file 10 — Source data Fig. 5 [file 44318_2025_652_MOESM10_ESM.zip › Figure 5/5A/5A_ETii_stack/modv0028.jpg]

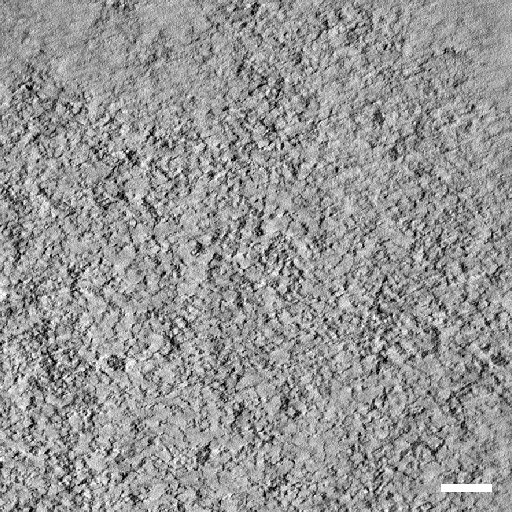

Supplement: Supplementary file 10 — Source data Fig. 5 [file 44318_2025_652_MOESM10_ESM.zip › Figure 5/5A/5A_ETii_stack/modv0014.jpg]

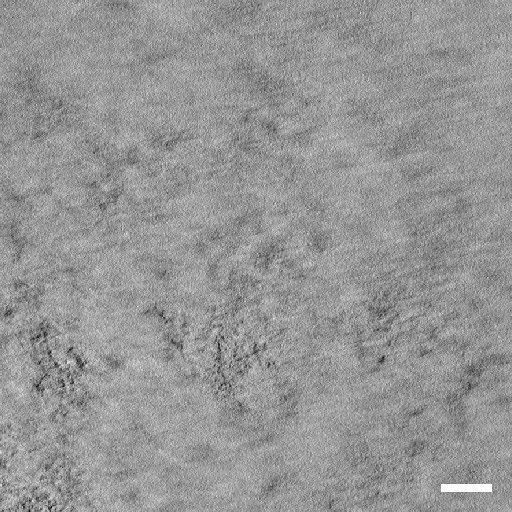

Supplement: Supplementary file 10 — Source data Fig. 5 [file 44318_2025_652_MOESM10_ESM.zip › Figure 5/5A/5A_ETii_stack/modv0000.jpg]

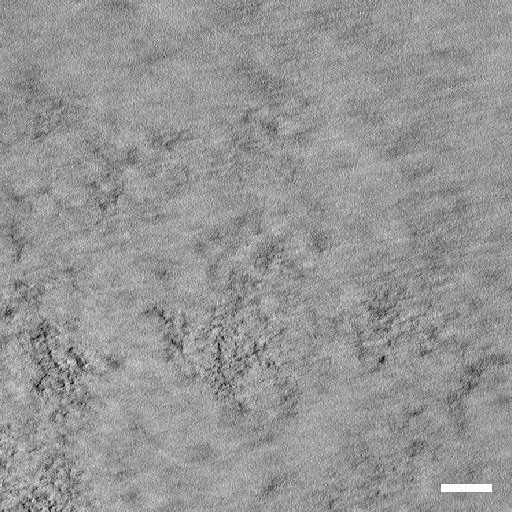

Supplement: Supplementary file 10 — Source data Fig. 5 [file 44318_2025_652_MOESM10_ESM.zip › Figure 5/5A/5A_ETii_stack/modv0001.jpg]

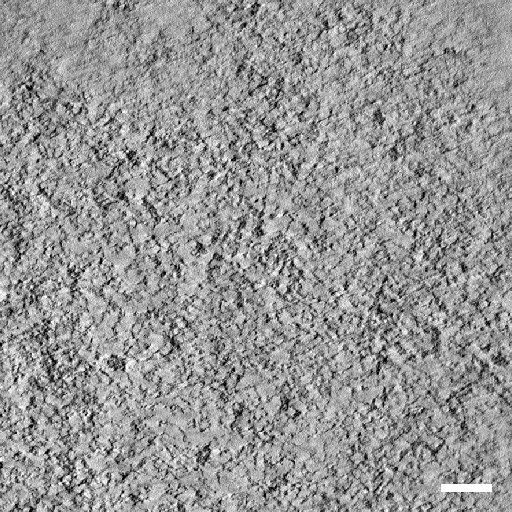

Supplement: Supplementary file 10 — Source data Fig. 5 [file 44318_2025_652_MOESM10_ESM.zip › Figure 5/5A/5A_ETii_stack/modv0015.jpg]

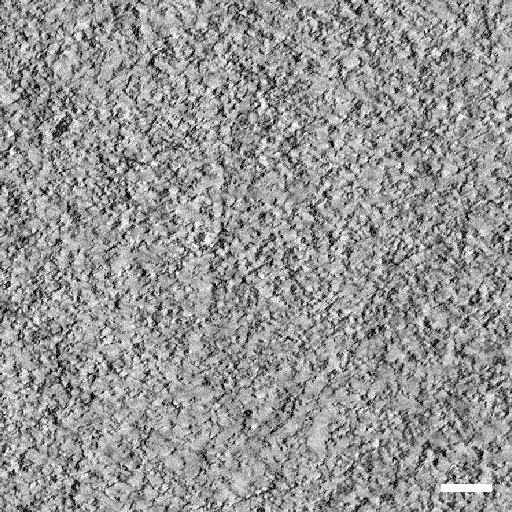

Supplement: Supplementary file 10 — Source data Fig. 5 [file 44318_2025_652_MOESM10_ESM.zip › Figure 5/5A/5A_ETii_stack/modv0029.jpg]

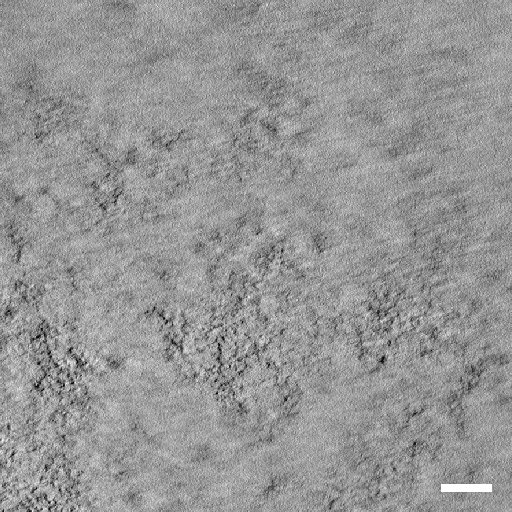

Supplement: Supplementary file 10 — Source data Fig. 5 [file 44318_2025_652_MOESM10_ESM.zip › Figure 5/5A/5A_ETii_stack/modv0003.jpg]

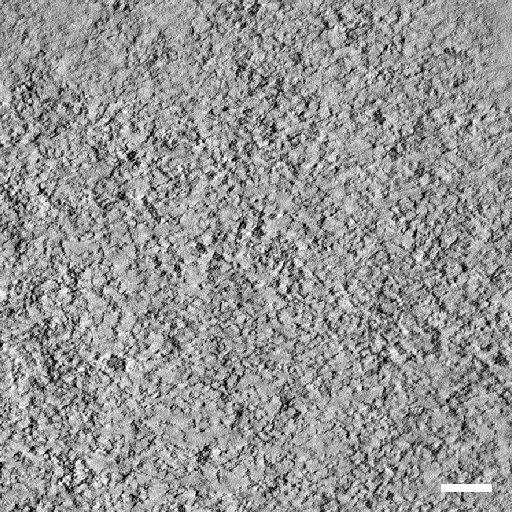

Supplement: Supplementary file 10 — Source data Fig. 5 [file 44318_2025_652_MOESM10_ESM.zip › Figure 5/5A/5A_ETii_stack/modv0017.jpg]
