# Supplementary figures and images for: Lenacapavir-induced capsid damage uncovers HIV-1 genomes emanating from nuclear speckles (part 2 of 3)
Source: EMBO J. 2025 Dec 1;45(2):449–70. doi: 10.1038/s44318-025-00652-5 (PMC12811339; doi:10.1038/s44318-025-00652-5)

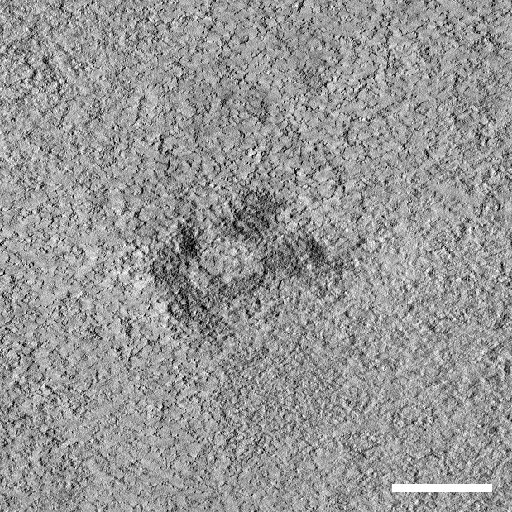

Supplement: Supplementary file 10 — Source data Fig. 5 [file 44318_2025_652_MOESM10_ESM.zip › Figure 5/5A/5A_ETi_stack/modv0314.jpg]

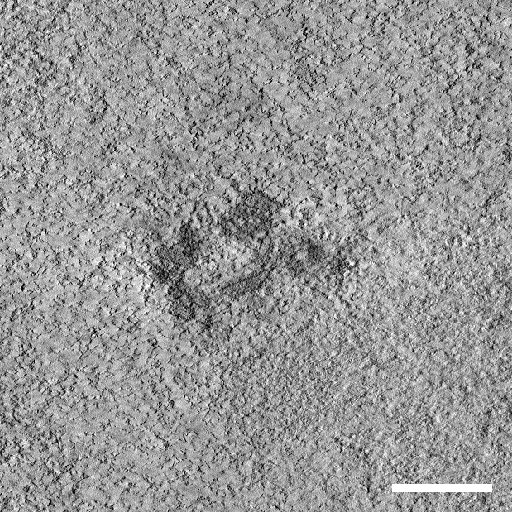

Supplement: Supplementary file 10 — Source data Fig. 5 [file 44318_2025_652_MOESM10_ESM.zip › Figure 5/5A/5A_ETi_stack/modv0300.jpg]

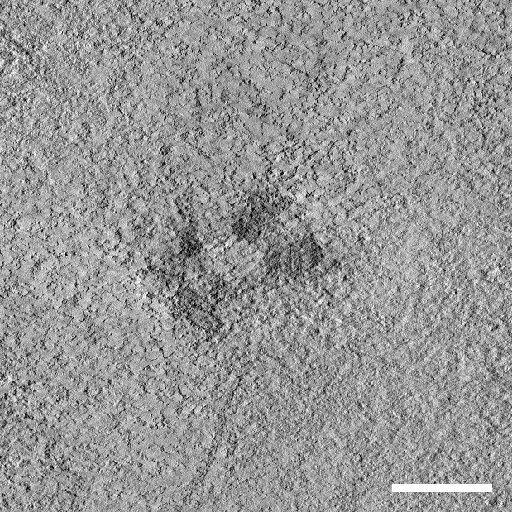

Supplement: Supplementary file 10 — Source data Fig. 5 [file 44318_2025_652_MOESM10_ESM.zip › Figure 5/5A/5A_ETi_stack/modv0328.jpg]

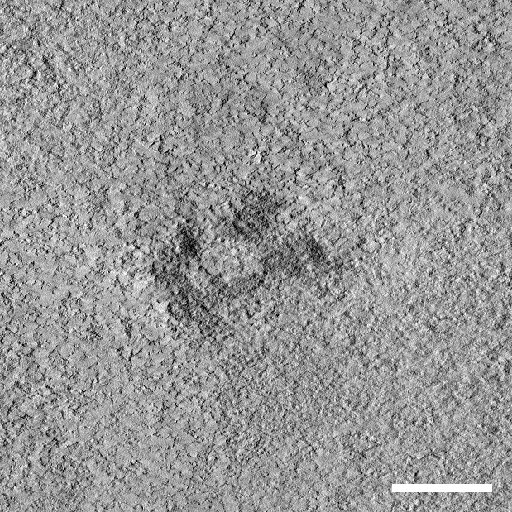

Supplement: Supplementary file 10 — Source data Fig. 5 [file 44318_2025_652_MOESM10_ESM.zip › Figure 5/5A/5A_ETi_stack/modv0316.jpg]

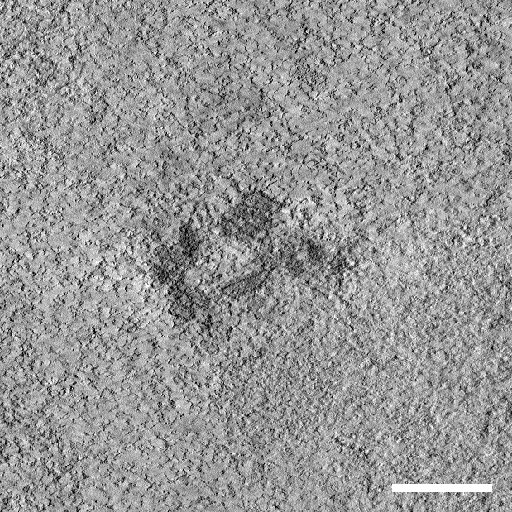

Supplement: Supplementary file 10 — Source data Fig. 5 [file 44318_2025_652_MOESM10_ESM.zip › Figure 5/5A/5A_ETi_stack/modv0302.jpg]

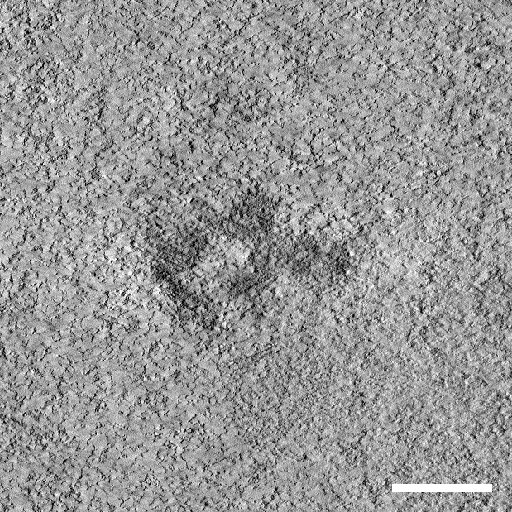

Supplement: Supplementary file 10 — Source data Fig. 5 [file 44318_2025_652_MOESM10_ESM.zip › Figure 5/5A/5A_ETi_stack/modv0289.jpg]

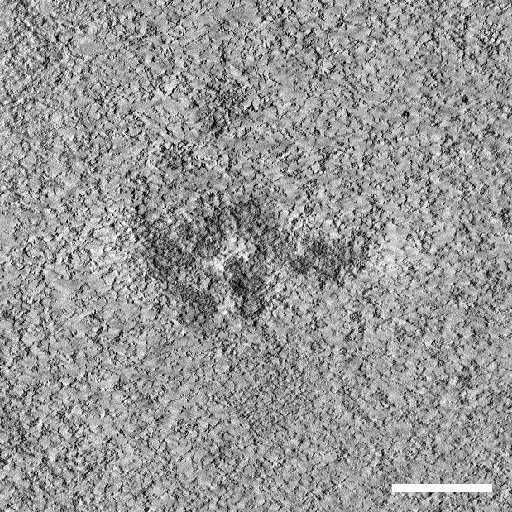

Supplement: Supplementary file 10 — Source data Fig. 5 [file 44318_2025_652_MOESM10_ESM.zip › Figure 5/5A/5A_ETi_stack/modv0262.jpg]

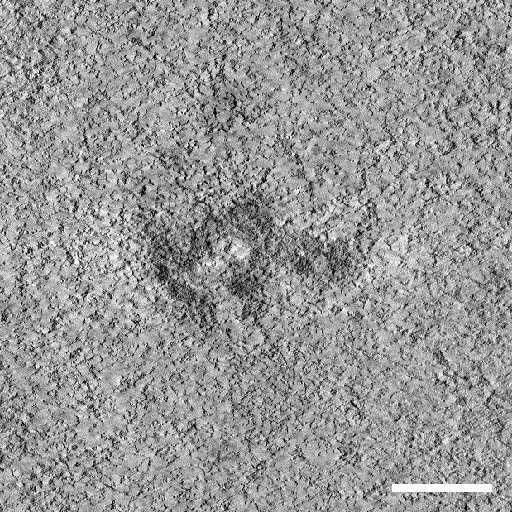

Supplement: Supplementary file 10 — Source data Fig. 5 [file 44318_2025_652_MOESM10_ESM.zip › Figure 5/5A/5A_ETi_stack/modv0276.jpg]

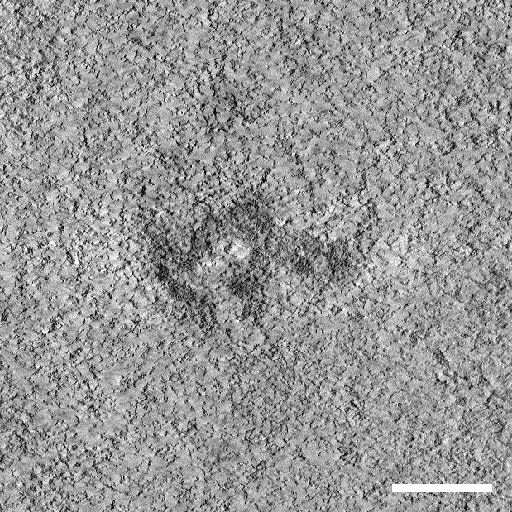

Supplement: Supplementary file 10 — Source data Fig. 5 [file 44318_2025_652_MOESM10_ESM.zip › Figure 5/5A/5A_ETi_stack/modv0277.jpg]

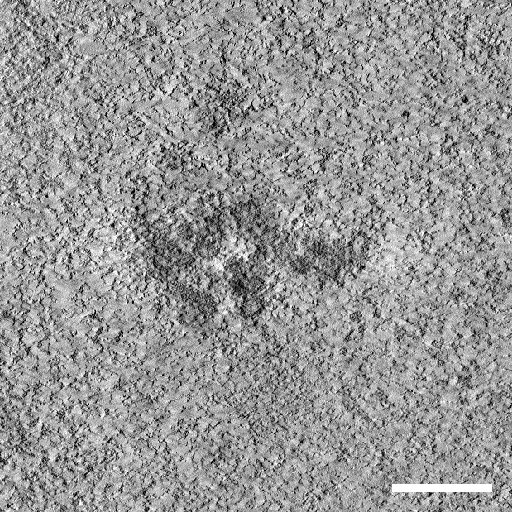

Supplement: Supplementary file 10 — Source data Fig. 5 [file 44318_2025_652_MOESM10_ESM.zip › Figure 5/5A/5A_ETi_stack/modv0263.jpg]

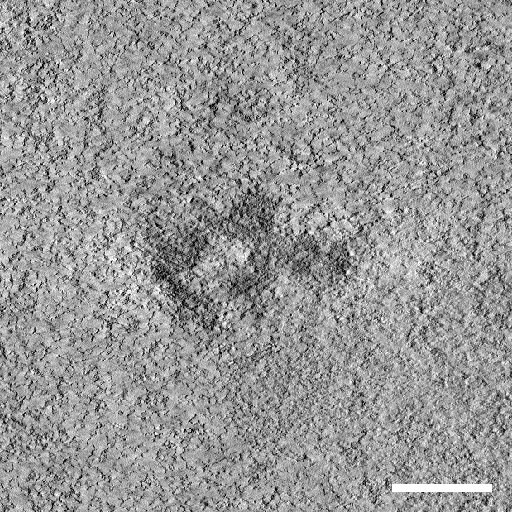

Supplement: Supplementary file 10 — Source data Fig. 5 [file 44318_2025_652_MOESM10_ESM.zip › Figure 5/5A/5A_ETi_stack/modv0288.jpg]

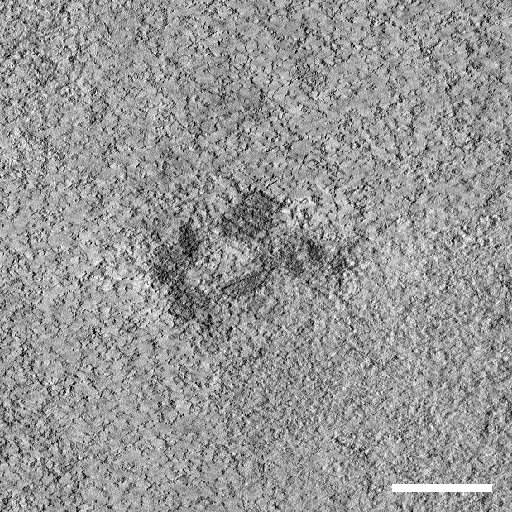

Supplement: Supplementary file 10 — Source data Fig. 5 [file 44318_2025_652_MOESM10_ESM.zip › Figure 5/5A/5A_ETi_stack/modv0303.jpg]

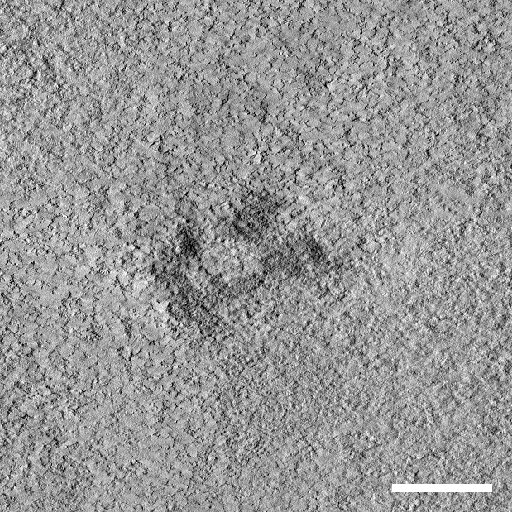

Supplement: Supplementary file 10 — Source data Fig. 5 [file 44318_2025_652_MOESM10_ESM.zip › Figure 5/5A/5A_ETi_stack/modv0317.jpg]

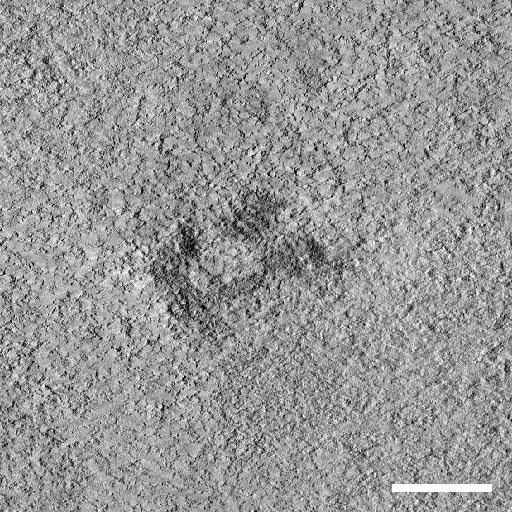

Supplement: Supplementary file 10 — Source data Fig. 5 [file 44318_2025_652_MOESM10_ESM.zip › Figure 5/5A/5A_ETi_stack/modv0313.jpg]

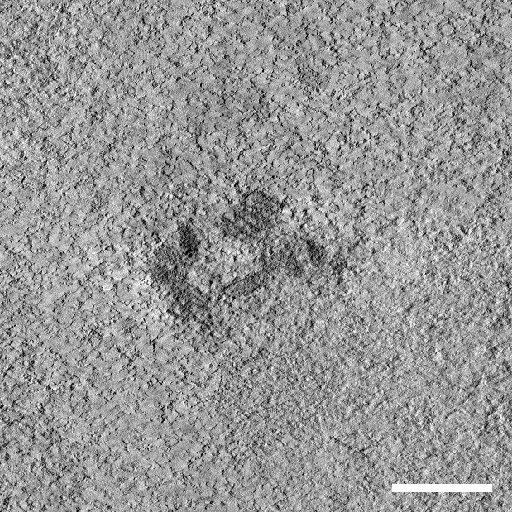

Supplement: Supplementary file 10 — Source data Fig. 5 [file 44318_2025_652_MOESM10_ESM.zip › Figure 5/5A/5A_ETi_stack/modv0307.jpg]

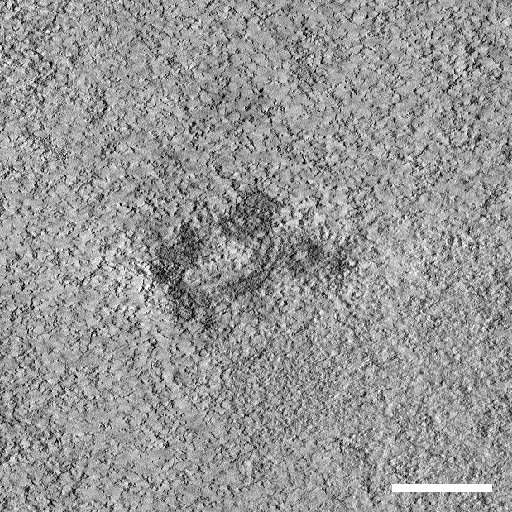

Supplement: Supplementary file 10 — Source data Fig. 5 [file 44318_2025_652_MOESM10_ESM.zip › Figure 5/5A/5A_ETi_stack/modv0298.jpg]

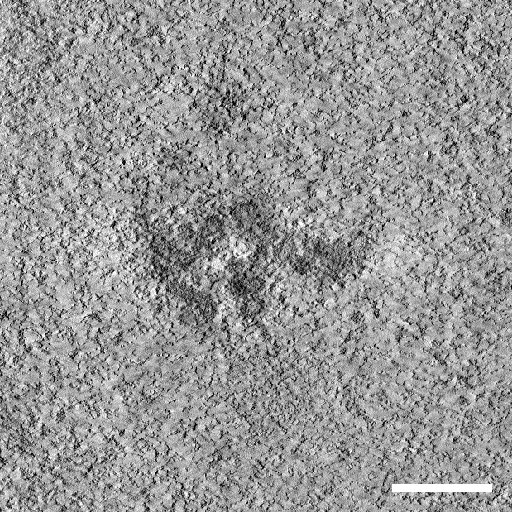

Supplement: Supplementary file 10 — Source data Fig. 5 [file 44318_2025_652_MOESM10_ESM.zip › Figure 5/5A/5A_ETi_stack/modv0267.jpg]

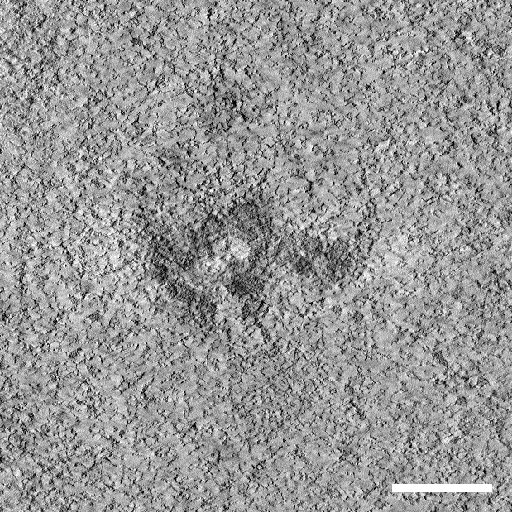

Supplement: Supplementary file 10 — Source data Fig. 5 [file 44318_2025_652_MOESM10_ESM.zip › Figure 5/5A/5A_ETi_stack/modv0273.jpg]

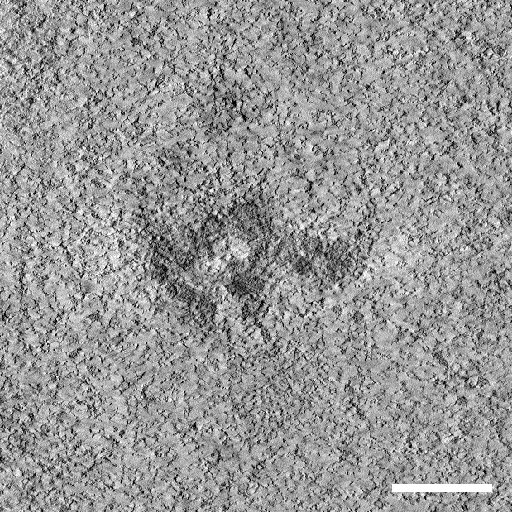

Supplement: Supplementary file 10 — Source data Fig. 5 [file 44318_2025_652_MOESM10_ESM.zip › Figure 5/5A/5A_ETi_stack/modv0272.jpg]

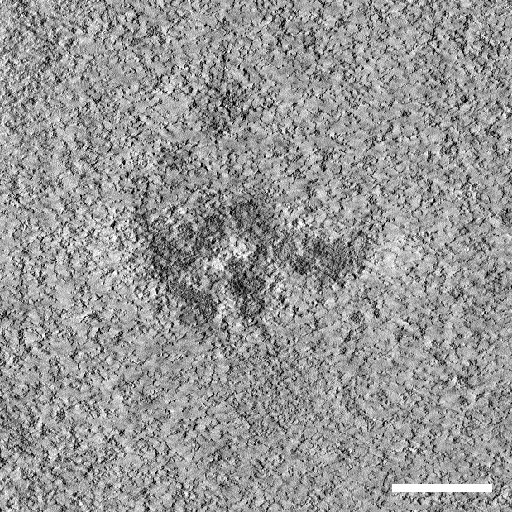

Supplement: Supplementary file 10 — Source data Fig. 5 [file 44318_2025_652_MOESM10_ESM.zip › Figure 5/5A/5A_ETi_stack/modv0266.jpg]

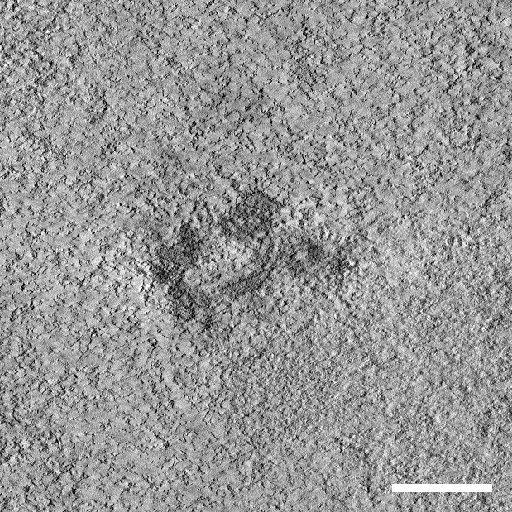

Supplement: Supplementary file 10 — Source data Fig. 5 [file 44318_2025_652_MOESM10_ESM.zip › Figure 5/5A/5A_ETi_stack/modv0299.jpg]

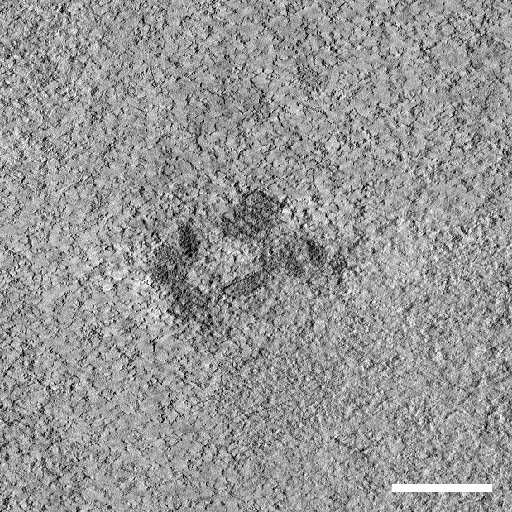

Supplement: Supplementary file 10 — Source data Fig. 5 [file 44318_2025_652_MOESM10_ESM.zip › Figure 5/5A/5A_ETi_stack/modv0306.jpg]

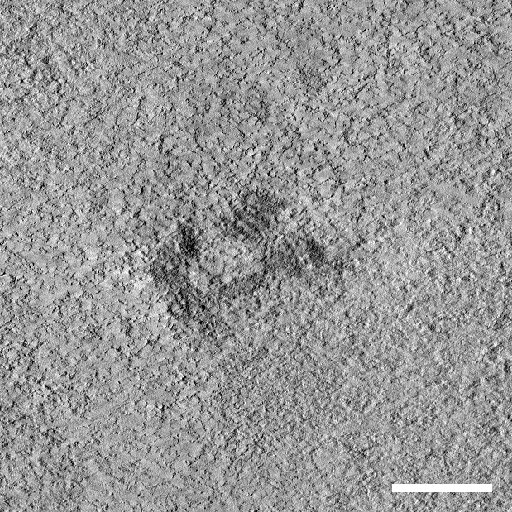

Supplement: Supplementary file 10 — Source data Fig. 5 [file 44318_2025_652_MOESM10_ESM.zip › Figure 5/5A/5A_ETi_stack/modv0312.jpg]

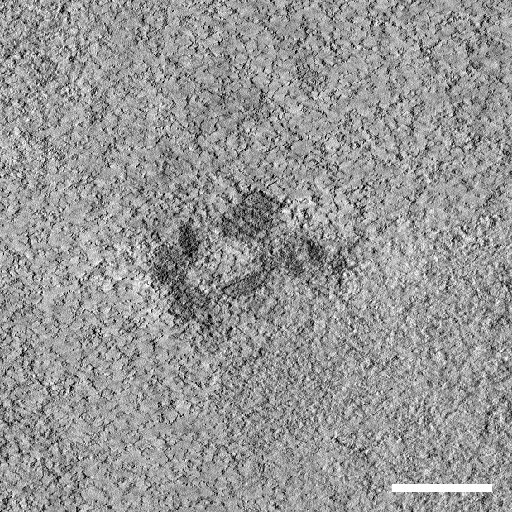

Supplement: Supplementary file 10 — Source data Fig. 5 [file 44318_2025_652_MOESM10_ESM.zip › Figure 5/5A/5A_ETi_stack/modv0304.jpg]

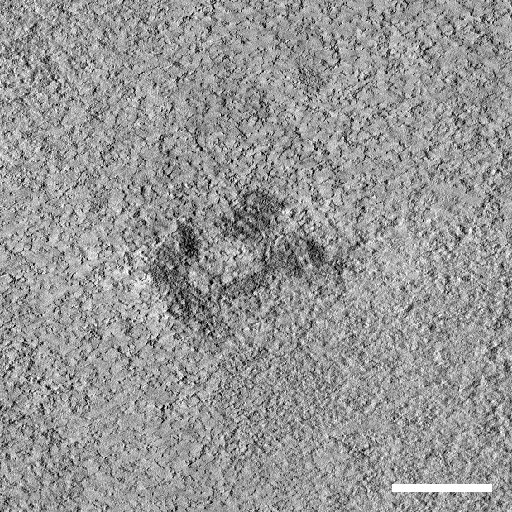

Supplement: Supplementary file 10 — Source data Fig. 5 [file 44318_2025_652_MOESM10_ESM.zip › Figure 5/5A/5A_ETi_stack/modv0310.jpg]

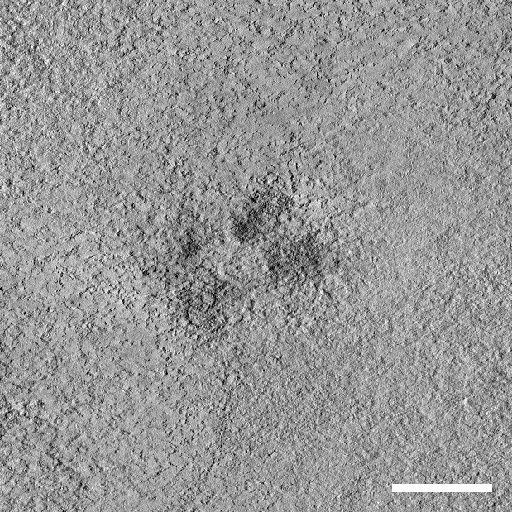

Supplement: Supplementary file 10 — Source data Fig. 5 [file 44318_2025_652_MOESM10_ESM.zip › Figure 5/5A/5A_ETi_stack/modv0338.jpg]

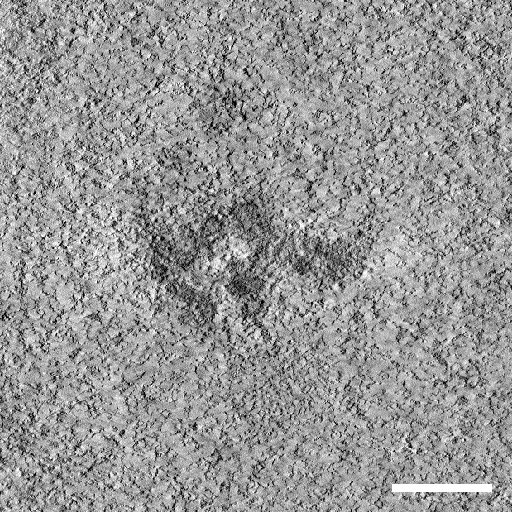

Supplement: Supplementary file 10 — Source data Fig. 5 [file 44318_2025_652_MOESM10_ESM.zip › Figure 5/5A/5A_ETi_stack/modv0270.jpg]

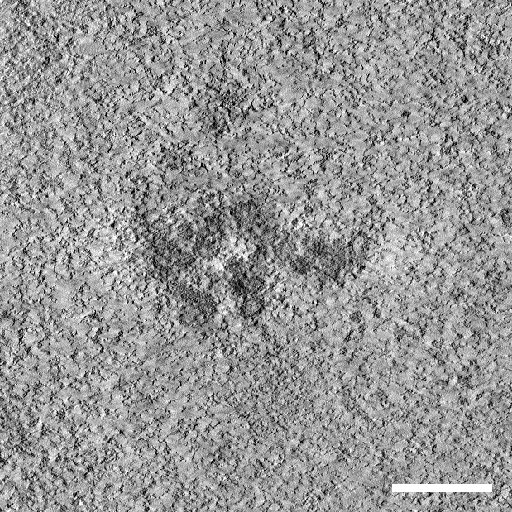

Supplement: Supplementary file 10 — Source data Fig. 5 [file 44318_2025_652_MOESM10_ESM.zip › Figure 5/5A/5A_ETi_stack/modv0264.jpg]

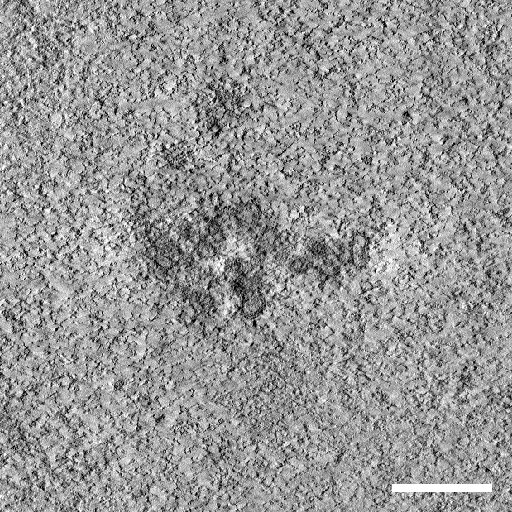

Supplement: Supplementary file 10 — Source data Fig. 5 [file 44318_2025_652_MOESM10_ESM.zip › Figure 5/5A/5A_ETi_stack/modv0258.jpg]

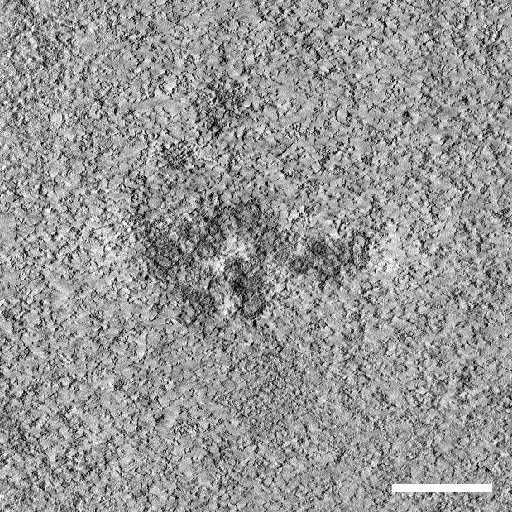

Supplement: Supplementary file 10 — Source data Fig. 5 [file 44318_2025_652_MOESM10_ESM.zip › Figure 5/5A/5A_ETi_stack/modv0259.jpg]

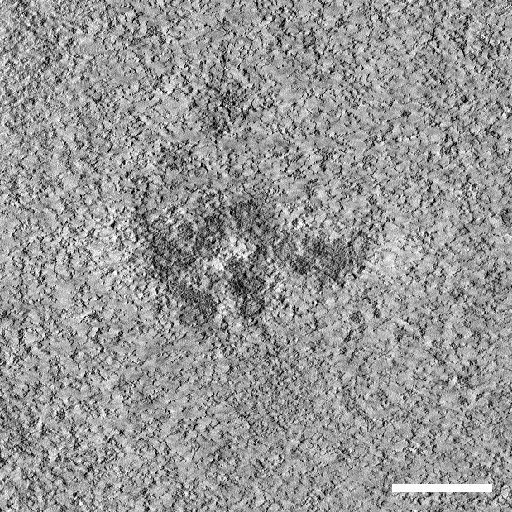

Supplement: Supplementary file 10 — Source data Fig. 5 [file 44318_2025_652_MOESM10_ESM.zip › Figure 5/5A/5A_ETi_stack/modv0265.jpg]

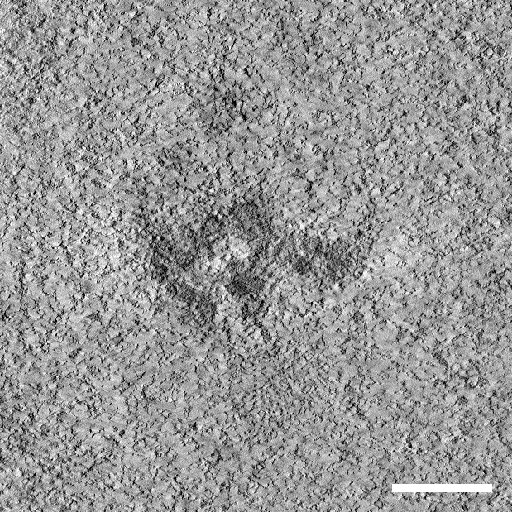

Supplement: Supplementary file 10 — Source data Fig. 5 [file 44318_2025_652_MOESM10_ESM.zip › Figure 5/5A/5A_ETi_stack/modv0271.jpg]

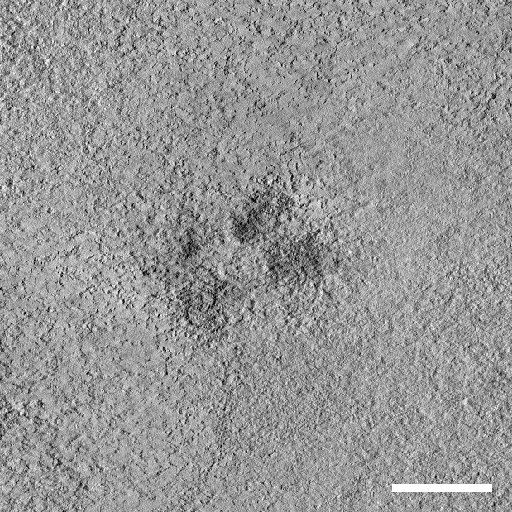

Supplement: Supplementary file 10 — Source data Fig. 5 [file 44318_2025_652_MOESM10_ESM.zip › Figure 5/5A/5A_ETi_stack/modv0339.jpg]

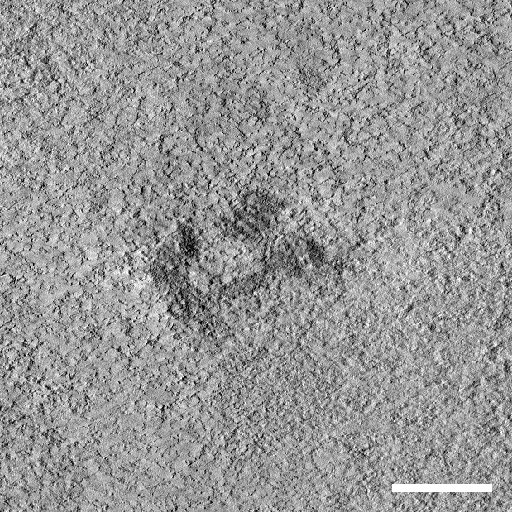

Supplement: Supplementary file 10 — Source data Fig. 5 [file 44318_2025_652_MOESM10_ESM.zip › Figure 5/5A/5A_ETi_stack/modv0311.jpg]

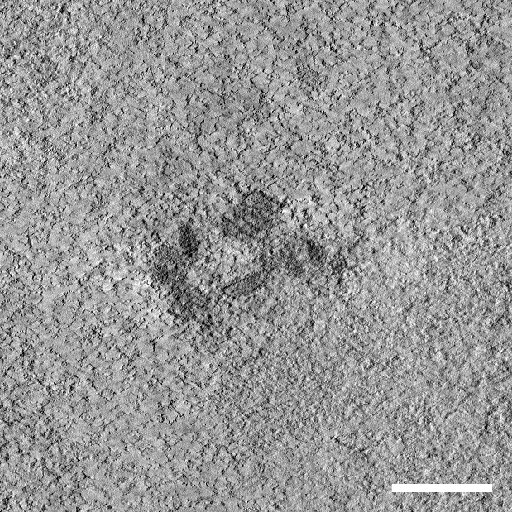

Supplement: Supplementary file 10 — Source data Fig. 5 [file 44318_2025_652_MOESM10_ESM.zip › Figure 5/5A/5A_ETi_stack/modv0305.jpg]

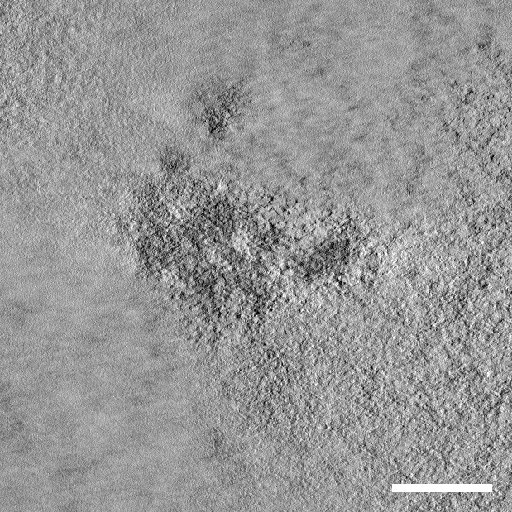

Supplement: Supplementary file 10 — Source data Fig. 5 [file 44318_2025_652_MOESM10_ESM.zip › Figure 5/5A/5A_ETi_stack/modv0216.jpg]

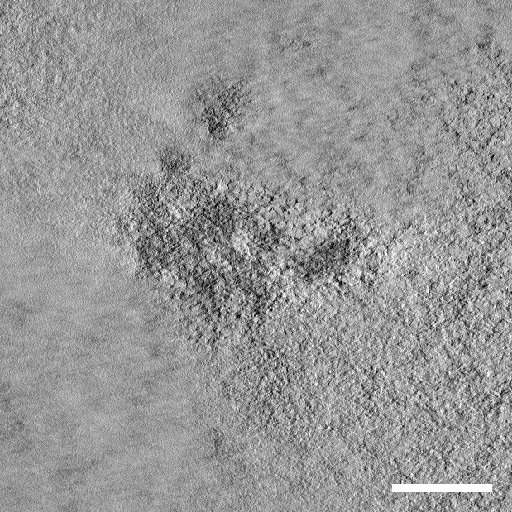

Supplement: Supplementary file 10 — Source data Fig. 5 [file 44318_2025_652_MOESM10_ESM.zip › Figure 5/5A/5A_ETi_stack/modv0217.jpg]

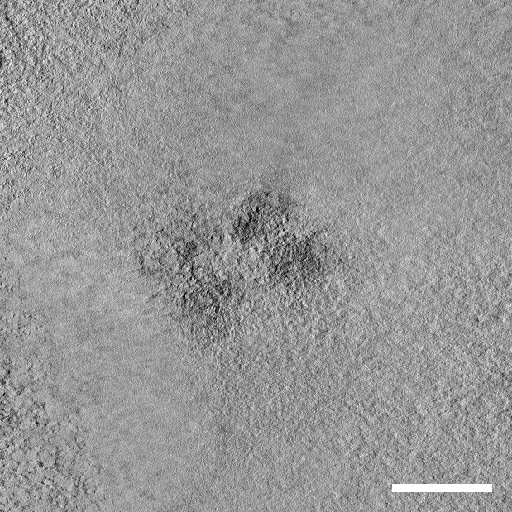

Supplement: Supplementary file 10 — Source data Fig. 5 [file 44318_2025_652_MOESM10_ESM.zip › Figure 5/5A/5A_ETi_stack/modv0349.jpg]

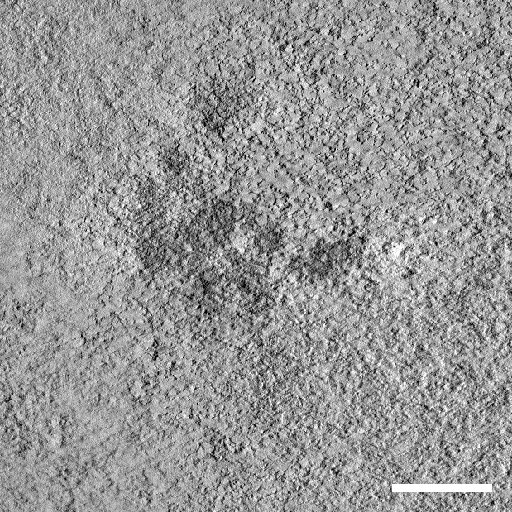

Supplement: Supplementary file 10 — Source data Fig. 5 [file 44318_2025_652_MOESM10_ESM.zip › Figure 5/5A/5A_ETi_stack/modv0229.jpg]

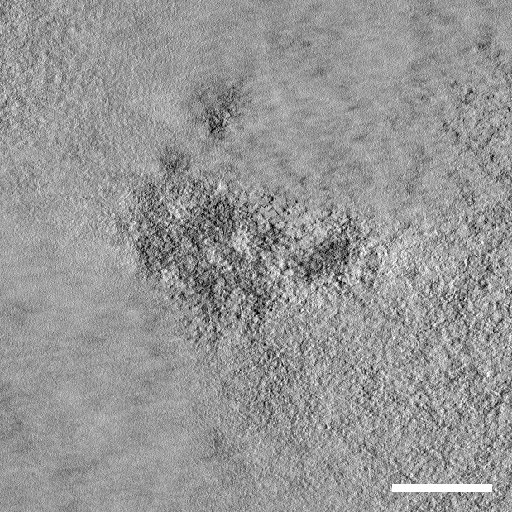

Supplement: Supplementary file 10 — Source data Fig. 5 [file 44318_2025_652_MOESM10_ESM.zip › Figure 5/5A/5A_ETi_stack/modv0215.jpg]

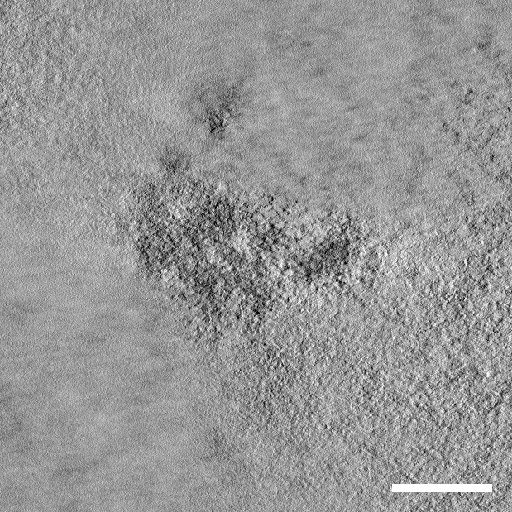

Supplement: Supplementary file 10 — Source data Fig. 5 [file 44318_2025_652_MOESM10_ESM.zip › Figure 5/5A/5A_ETi_stack/modv0214.jpg]

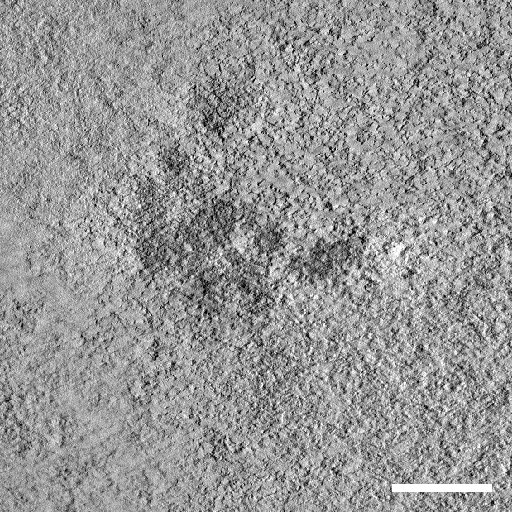

Supplement: Supplementary file 10 — Source data Fig. 5 [file 44318_2025_652_MOESM10_ESM.zip › Figure 5/5A/5A_ETi_stack/modv0228.jpg]

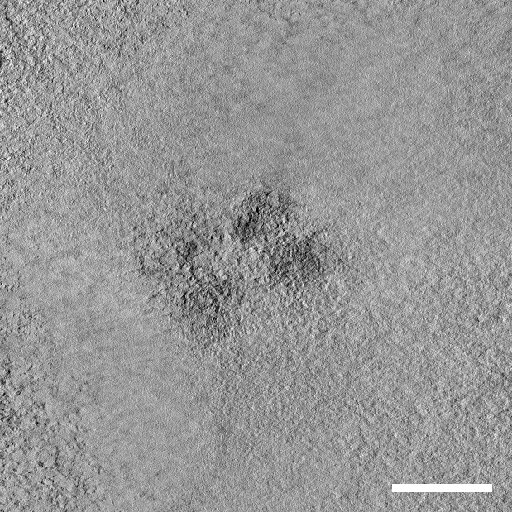

Supplement: Supplementary file 10 — Source data Fig. 5 [file 44318_2025_652_MOESM10_ESM.zip › Figure 5/5A/5A_ETi_stack/modv0348.jpg]

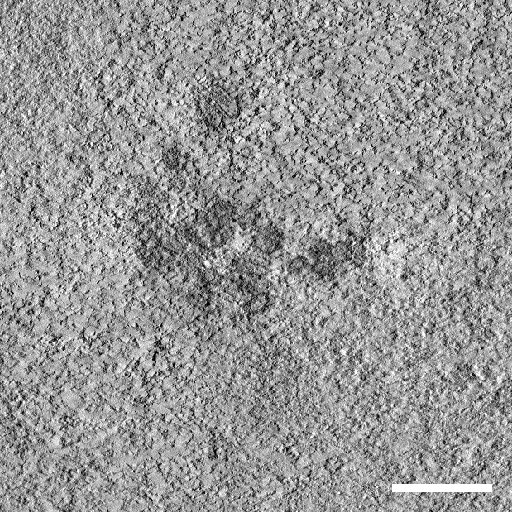

Supplement: Supplementary file 10 — Source data Fig. 5 [file 44318_2025_652_MOESM10_ESM.zip › Figure 5/5A/5A_ETi_stack/modv0238.jpg]

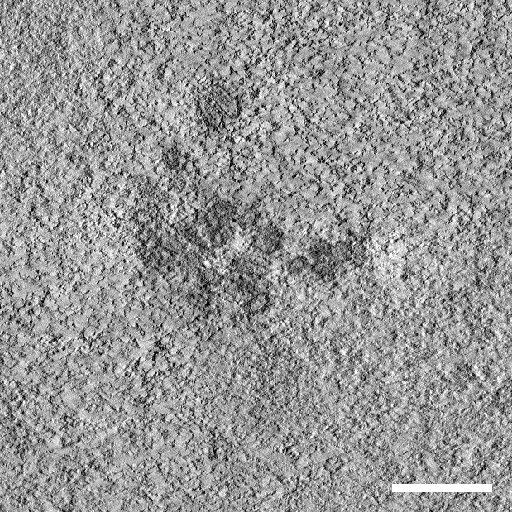

Supplement: Supplementary file 10 — Source data Fig. 5 [file 44318_2025_652_MOESM10_ESM.zip › Figure 5/5A/5A_ETi_stack/modv0239.jpg]

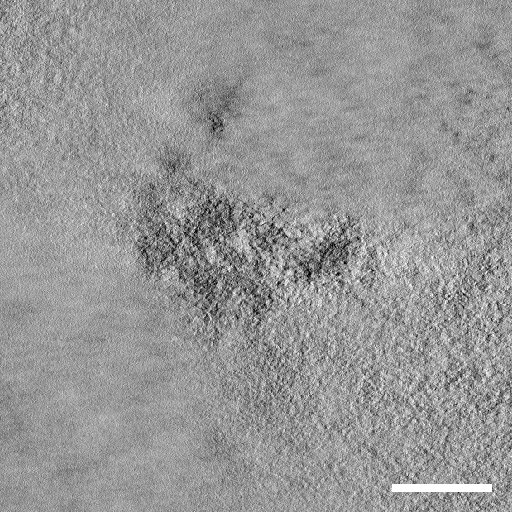

Supplement: Supplementary file 10 — Source data Fig. 5 [file 44318_2025_652_MOESM10_ESM.zip › Figure 5/5A/5A_ETi_stack/modv0211.jpg]

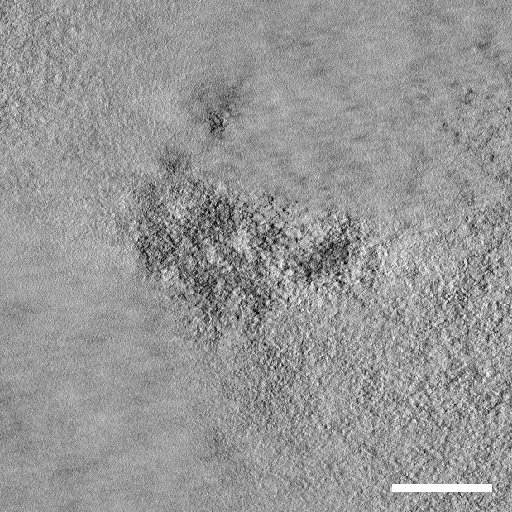

Supplement: Supplementary file 10 — Source data Fig. 5 [file 44318_2025_652_MOESM10_ESM.zip › Figure 5/5A/5A_ETi_stack/modv0213.jpg]

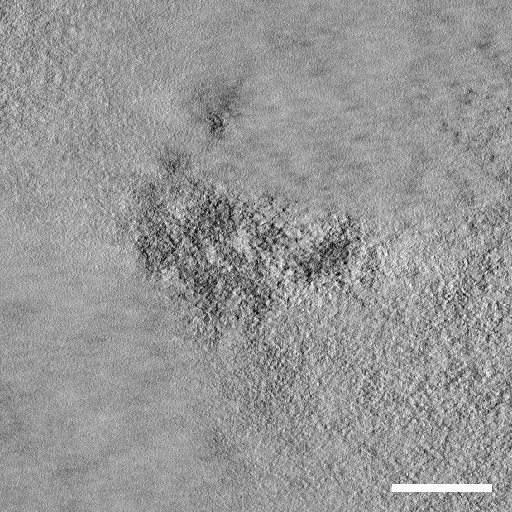

Supplement: Supplementary file 10 — Source data Fig. 5 [file 44318_2025_652_MOESM10_ESM.zip › Figure 5/5A/5A_ETi_stack/modv0212.jpg]

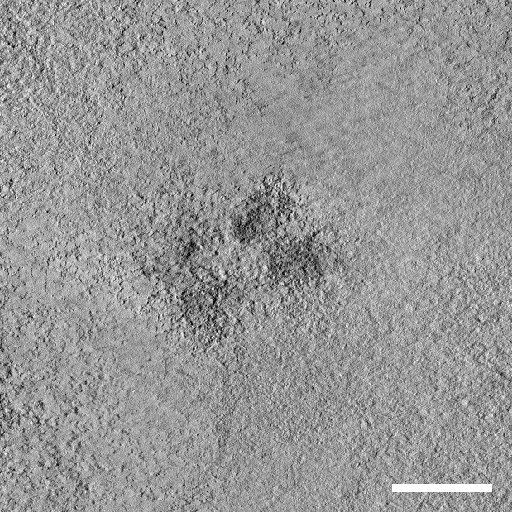

Supplement: Supplementary file 10 — Source data Fig. 5 [file 44318_2025_652_MOESM10_ESM.zip › Figure 5/5A/5A_ETi_stack/modv0343.jpg]

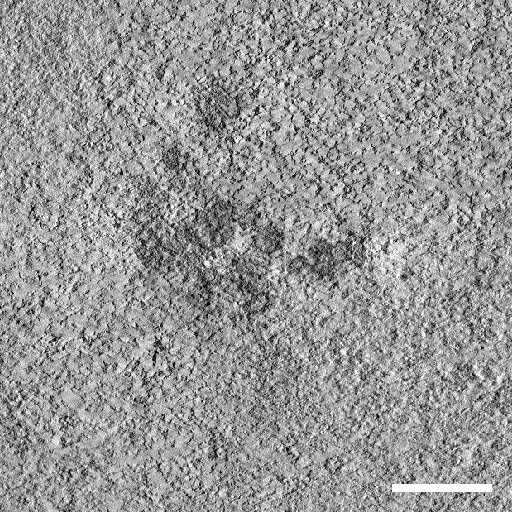

Supplement: Supplementary file 10 — Source data Fig. 5 [file 44318_2025_652_MOESM10_ESM.zip › Figure 5/5A/5A_ETi_stack/modv0237.jpg]

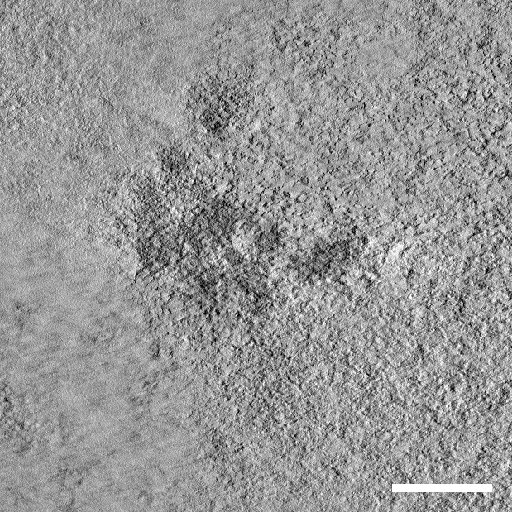

Supplement: Supplementary file 10 — Source data Fig. 5 [file 44318_2025_652_MOESM10_ESM.zip › Figure 5/5A/5A_ETi_stack/modv0223.jpg]

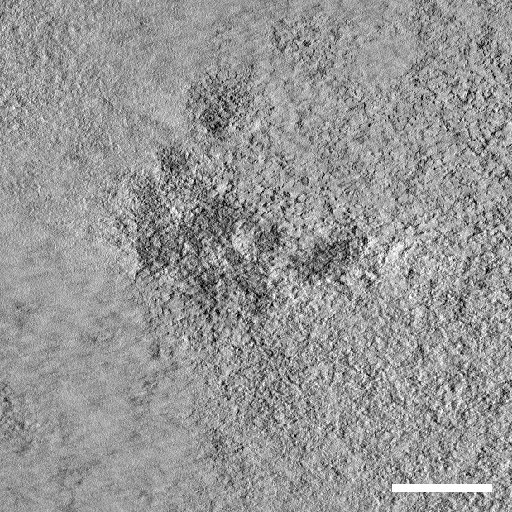

Supplement: Supplementary file 10 — Source data Fig. 5 [file 44318_2025_652_MOESM10_ESM.zip › Figure 5/5A/5A_ETi_stack/modv0222.jpg]

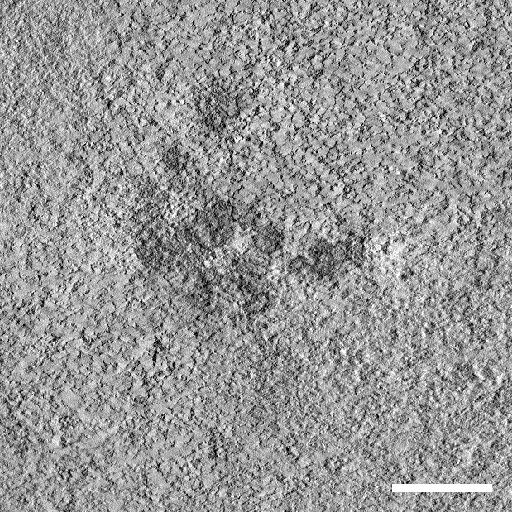

Supplement: Supplementary file 10 — Source data Fig. 5 [file 44318_2025_652_MOESM10_ESM.zip › Figure 5/5A/5A_ETi_stack/modv0236.jpg]

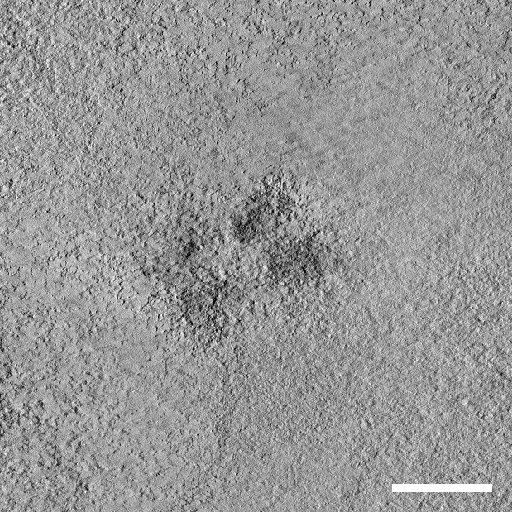

Supplement: Supplementary file 10 — Source data Fig. 5 [file 44318_2025_652_MOESM10_ESM.zip › Figure 5/5A/5A_ETi_stack/modv0342.jpg]

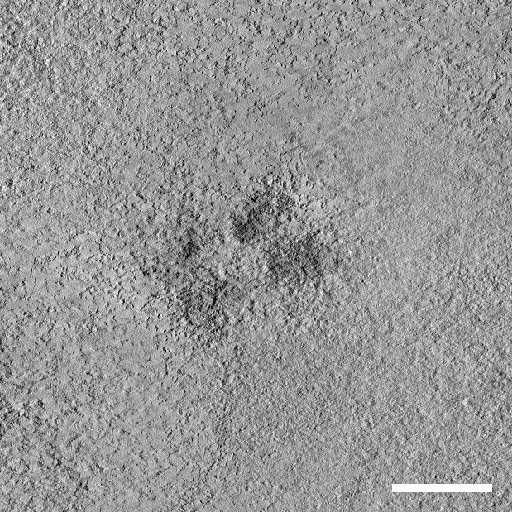

Supplement: Supplementary file 10 — Source data Fig. 5 [file 44318_2025_652_MOESM10_ESM.zip › Figure 5/5A/5A_ETi_stack/modv0340.jpg]

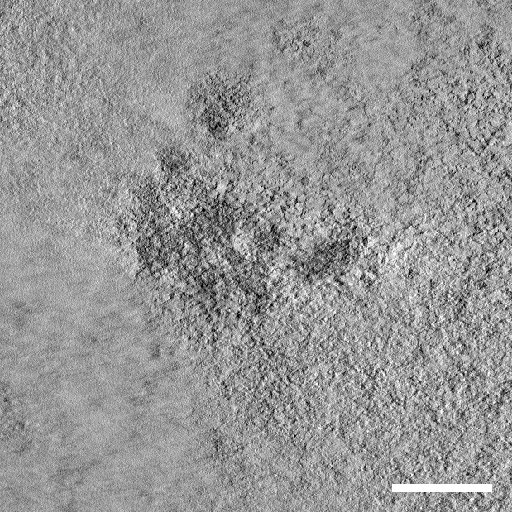

Supplement: Supplementary file 10 — Source data Fig. 5 [file 44318_2025_652_MOESM10_ESM.zip › Figure 5/5A/5A_ETi_stack/modv0220.jpg]

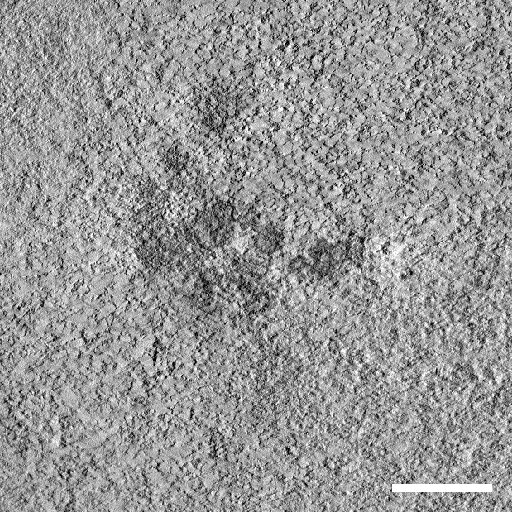

Supplement: Supplementary file 10 — Source data Fig. 5 [file 44318_2025_652_MOESM10_ESM.zip › Figure 5/5A/5A_ETi_stack/modv0234.jpg]

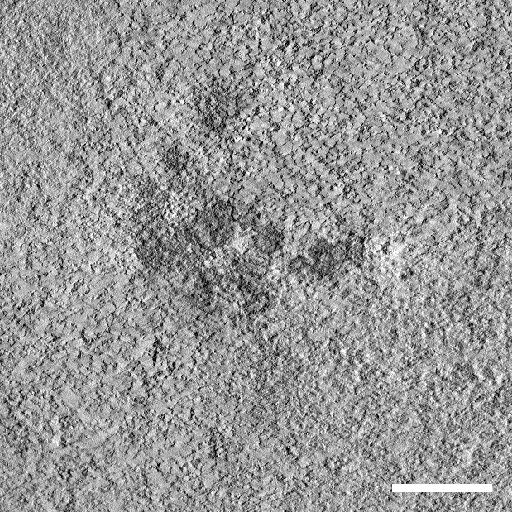

Supplement: Supplementary file 10 — Source data Fig. 5 [file 44318_2025_652_MOESM10_ESM.zip › Figure 5/5A/5A_ETi_stack/modv0235.jpg]

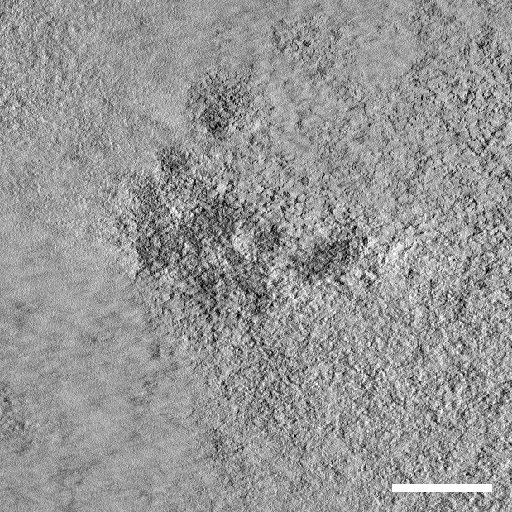

Supplement: Supplementary file 10 — Source data Fig. 5 [file 44318_2025_652_MOESM10_ESM.zip › Figure 5/5A/5A_ETi_stack/modv0221.jpg]

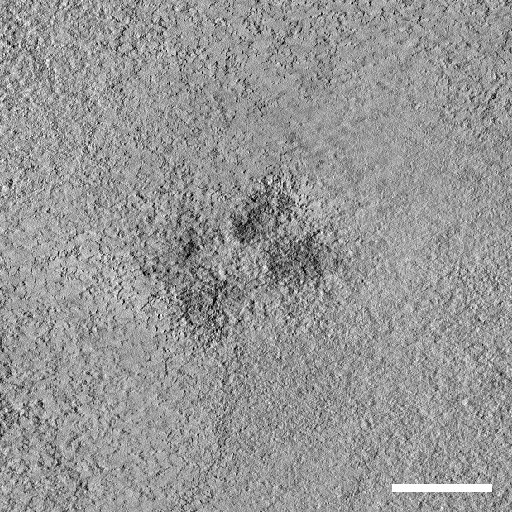

Supplement: Supplementary file 10 — Source data Fig. 5 [file 44318_2025_652_MOESM10_ESM.zip › Figure 5/5A/5A_ETi_stack/modv0341.jpg]

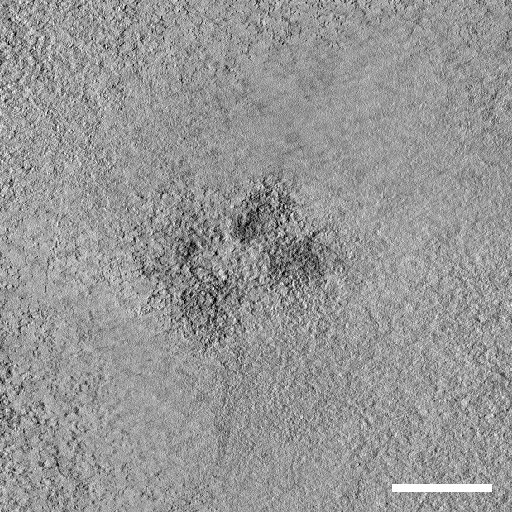

Supplement: Supplementary file 10 — Source data Fig. 5 [file 44318_2025_652_MOESM10_ESM.zip › Figure 5/5A/5A_ETi_stack/modv0345.jpg]

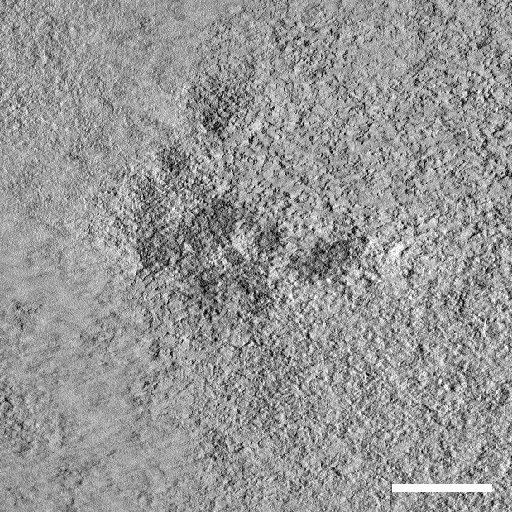

Supplement: Supplementary file 10 — Source data Fig. 5 [file 44318_2025_652_MOESM10_ESM.zip › Figure 5/5A/5A_ETi_stack/modv0225.jpg]

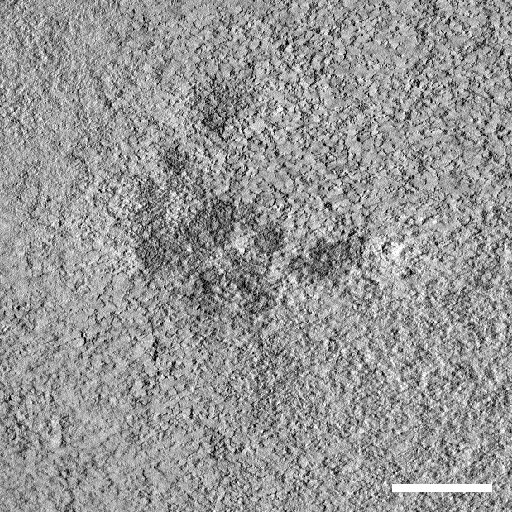

Supplement: Supplementary file 10 — Source data Fig. 5 [file 44318_2025_652_MOESM10_ESM.zip › Figure 5/5A/5A_ETi_stack/modv0231.jpg]

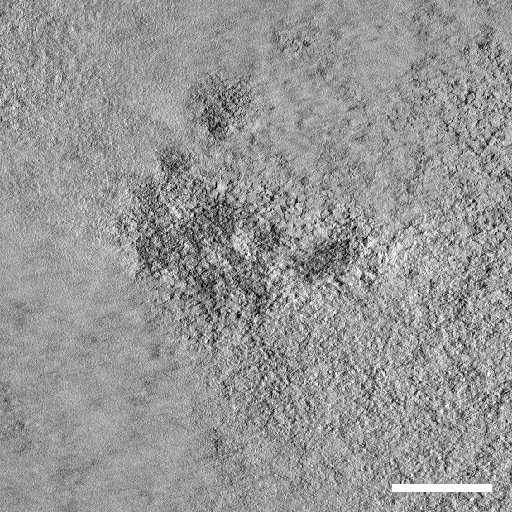

Supplement: Supplementary file 10 — Source data Fig. 5 [file 44318_2025_652_MOESM10_ESM.zip › Figure 5/5A/5A_ETi_stack/modv0219.jpg]

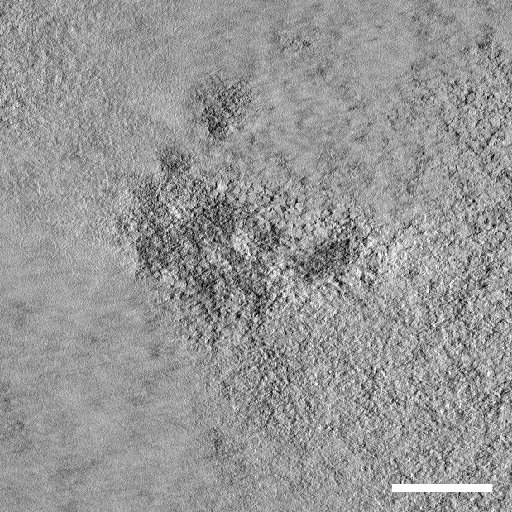

Supplement: Supplementary file 10 — Source data Fig. 5 [file 44318_2025_652_MOESM10_ESM.zip › Figure 5/5A/5A_ETi_stack/modv0218.jpg]

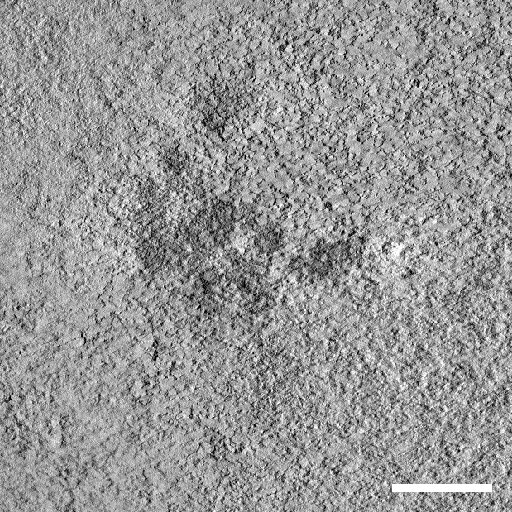

Supplement: Supplementary file 10 — Source data Fig. 5 [file 44318_2025_652_MOESM10_ESM.zip › Figure 5/5A/5A_ETi_stack/modv0230.jpg]

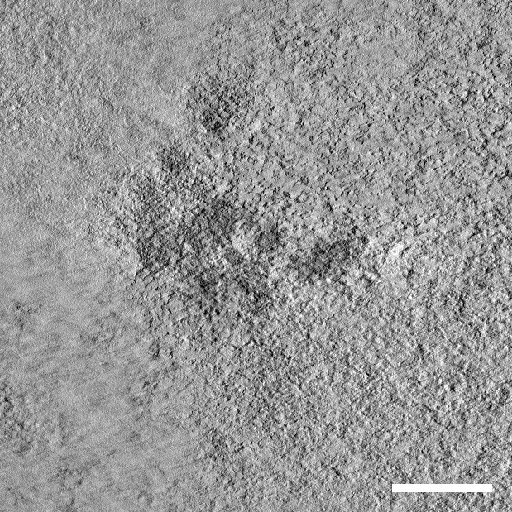

Supplement: Supplementary file 10 — Source data Fig. 5 [file 44318_2025_652_MOESM10_ESM.zip › Figure 5/5A/5A_ETi_stack/modv0224.jpg]

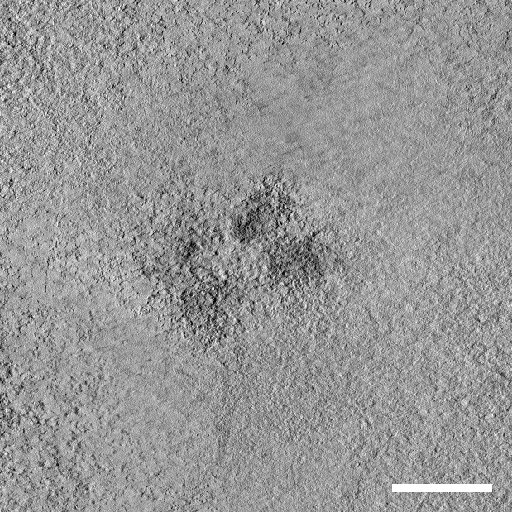

Supplement: Supplementary file 10 — Source data Fig. 5 [file 44318_2025_652_MOESM10_ESM.zip › Figure 5/5A/5A_ETi_stack/modv0344.jpg]

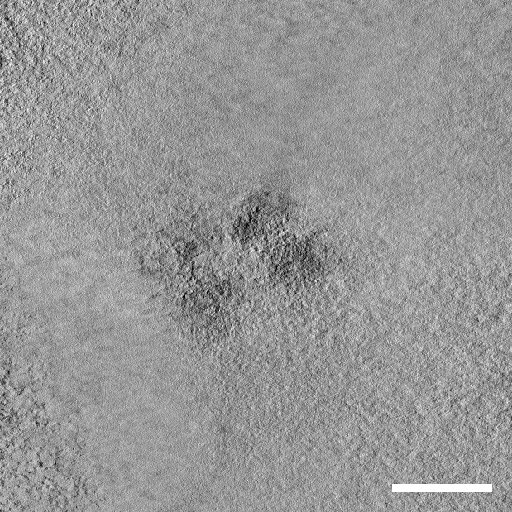

Supplement: Supplementary file 10 — Source data Fig. 5 [file 44318_2025_652_MOESM10_ESM.zip › Figure 5/5A/5A_ETi_stack/modv0350.jpg]

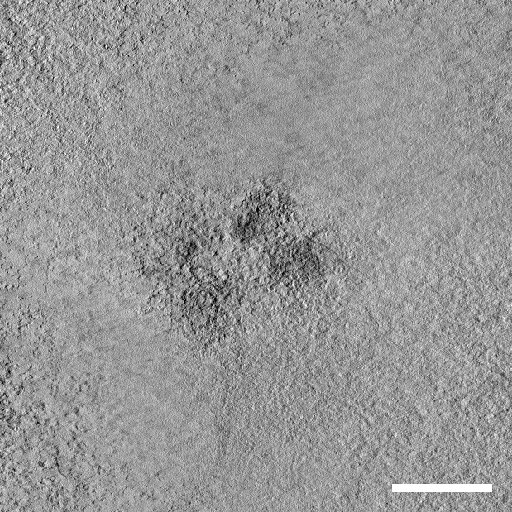

Supplement: Supplementary file 10 — Source data Fig. 5 [file 44318_2025_652_MOESM10_ESM.zip › Figure 5/5A/5A_ETi_stack/modv0346.jpg]

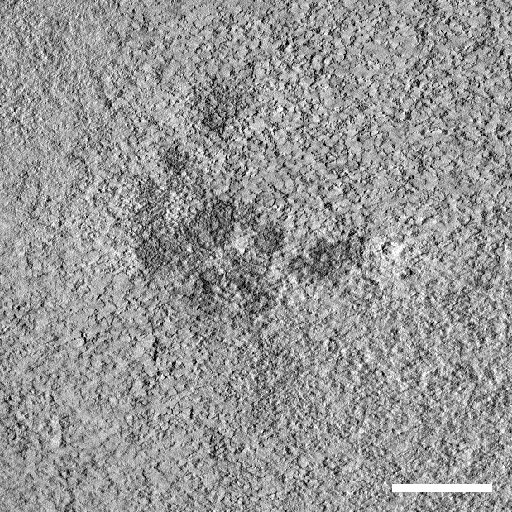

Supplement: Supplementary file 10 — Source data Fig. 5 [file 44318_2025_652_MOESM10_ESM.zip › Figure 5/5A/5A_ETi_stack/modv0232.jpg]

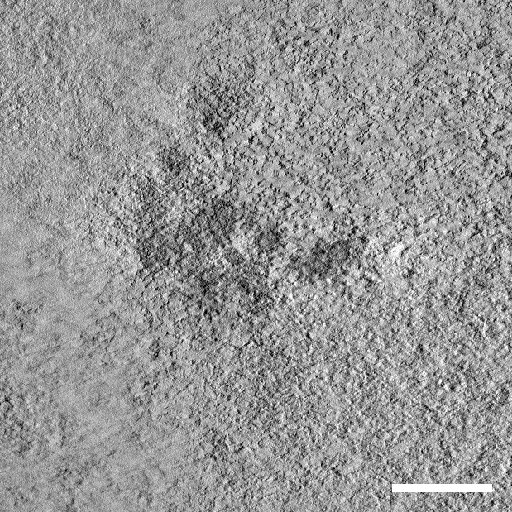

Supplement: Supplementary file 10 — Source data Fig. 5 [file 44318_2025_652_MOESM10_ESM.zip › Figure 5/5A/5A_ETi_stack/modv0226.jpg]

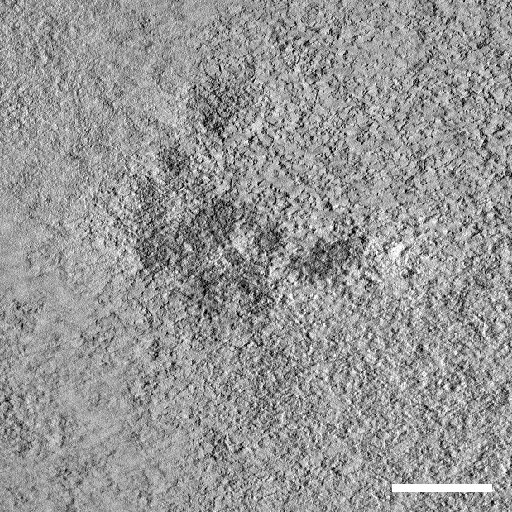

Supplement: Supplementary file 10 — Source data Fig. 5 [file 44318_2025_652_MOESM10_ESM.zip › Figure 5/5A/5A_ETi_stack/modv0227.jpg]

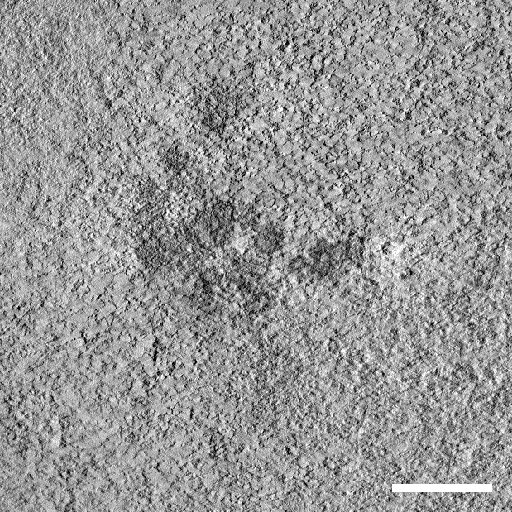

Supplement: Supplementary file 10 — Source data Fig. 5 [file 44318_2025_652_MOESM10_ESM.zip › Figure 5/5A/5A_ETi_stack/modv0233.jpg]

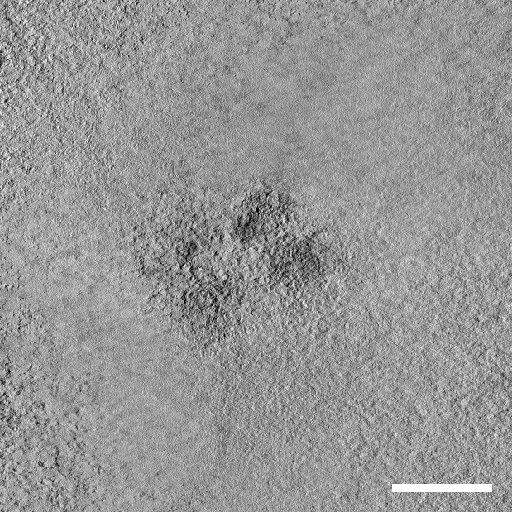

Supplement: Supplementary file 10 — Source data Fig. 5 [file 44318_2025_652_MOESM10_ESM.zip › Figure 5/5A/5A_ETi_stack/modv0347.jpg]

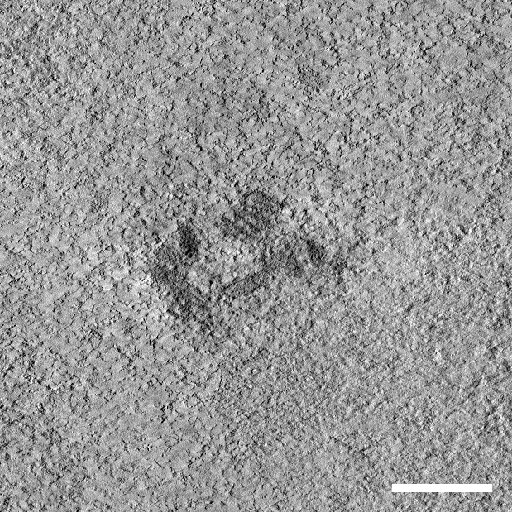

Supplement: Supplementary file 10 — Source data Fig. 5 [file 44318_2025_652_MOESM10_ESM.zip › Figure 5/5A/5A_ETi_stack/modv0308.jpg]

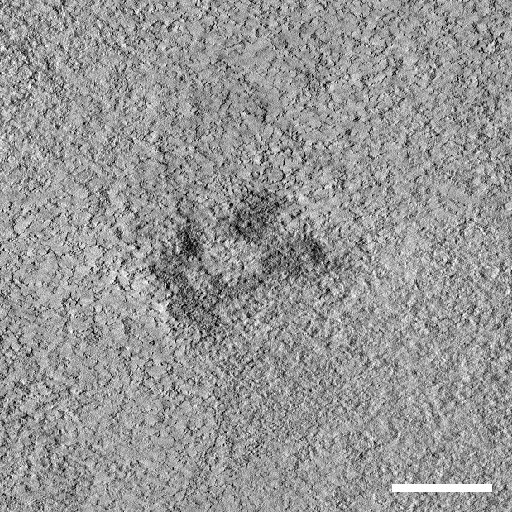

Supplement: Supplementary file 10 — Source data Fig. 5 [file 44318_2025_652_MOESM10_ESM.zip › Figure 5/5A/5A_ETi_stack/modv0320.jpg]

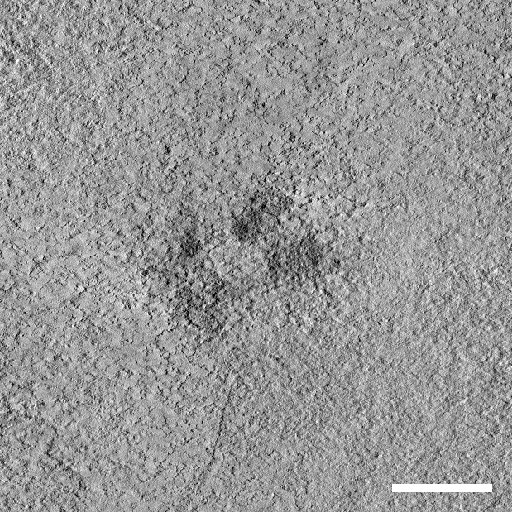

Supplement: Supplementary file 10 — Source data Fig. 5 [file 44318_2025_652_MOESM10_ESM.zip › Figure 5/5A/5A_ETi_stack/modv0334.jpg]

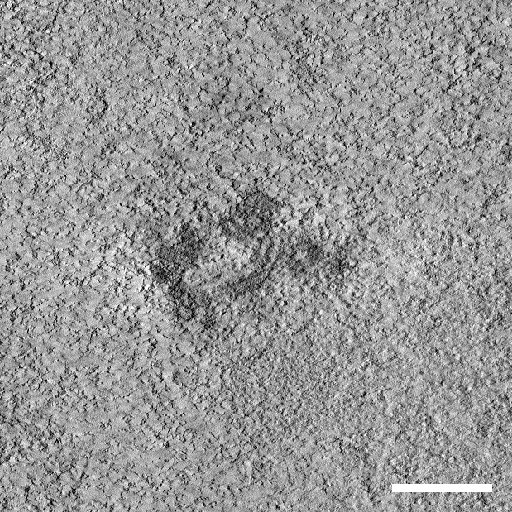

Supplement: Supplementary file 10 — Source data Fig. 5 [file 44318_2025_652_MOESM10_ESM.zip › Figure 5/5A/5A_ETi_stack/modv0297.jpg]

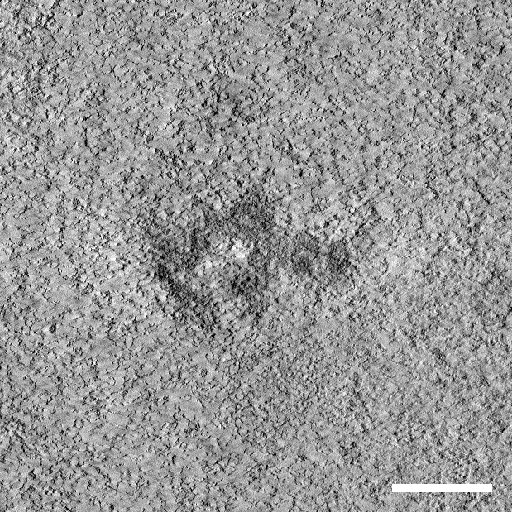

Supplement: Supplementary file 10 — Source data Fig. 5 [file 44318_2025_652_MOESM10_ESM.zip › Figure 5/5A/5A_ETi_stack/modv0283.jpg]

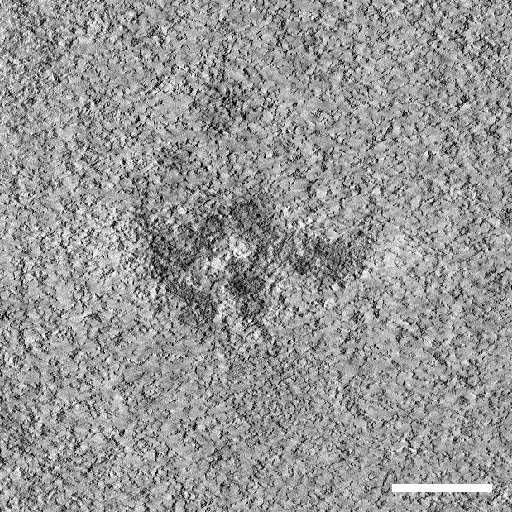

Supplement: Supplementary file 10 — Source data Fig. 5 [file 44318_2025_652_MOESM10_ESM.zip › Figure 5/5A/5A_ETi_stack/modv0268.jpg]

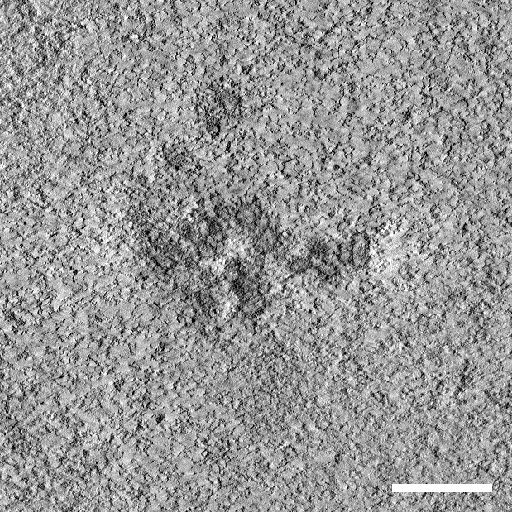

Supplement: Supplementary file 10 — Source data Fig. 5 [file 44318_2025_652_MOESM10_ESM.zip › Figure 5/5A/5A_ETi_stack/modv0254.jpg]

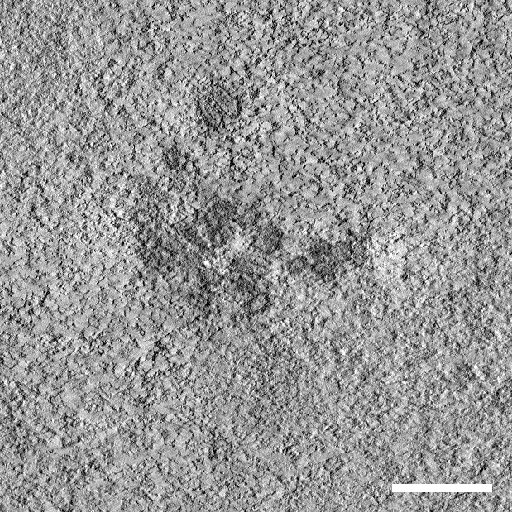

Supplement: Supplementary file 10 — Source data Fig. 5 [file 44318_2025_652_MOESM10_ESM.zip › Figure 5/5A/5A_ETi_stack/modv0240.jpg]

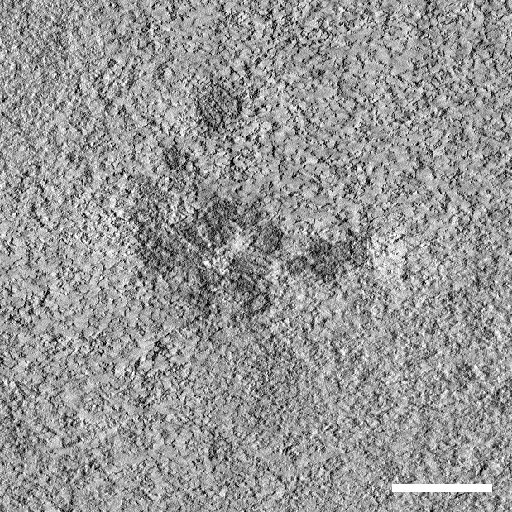

Supplement: Supplementary file 10 — Source data Fig. 5 [file 44318_2025_652_MOESM10_ESM.zip › Figure 5/5A/5A_ETi_stack/modv0241.jpg]

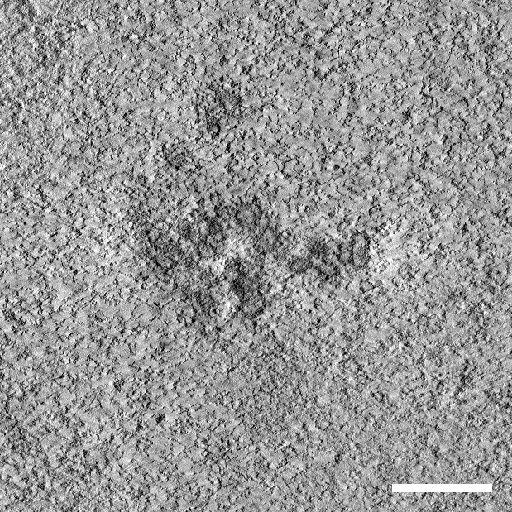

Supplement: Supplementary file 10 — Source data Fig. 5 [file 44318_2025_652_MOESM10_ESM.zip › Figure 5/5A/5A_ETi_stack/modv0255.jpg]

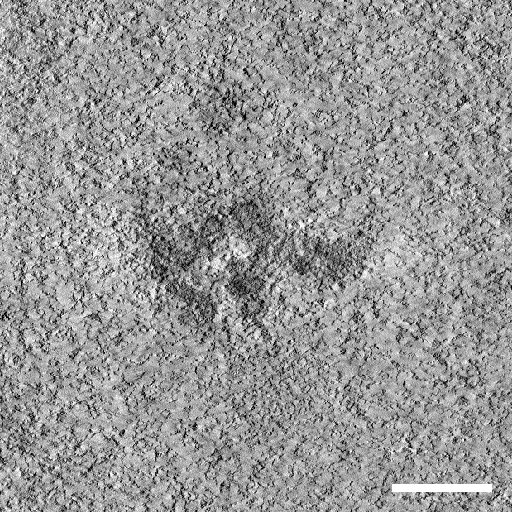

Supplement: Supplementary file 10 — Source data Fig. 5 [file 44318_2025_652_MOESM10_ESM.zip › Figure 5/5A/5A_ETi_stack/modv0269.jpg]

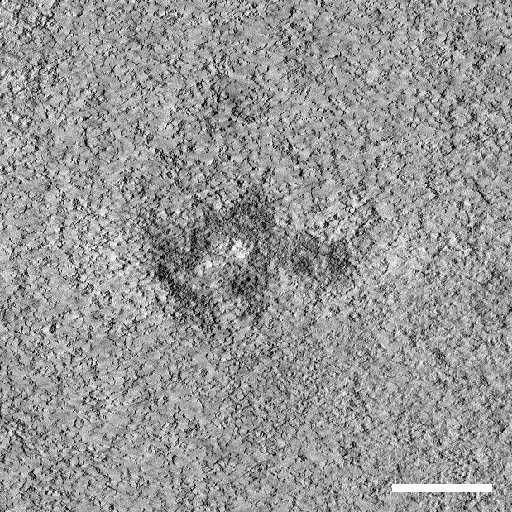

Supplement: Supplementary file 10 — Source data Fig. 5 [file 44318_2025_652_MOESM10_ESM.zip › Figure 5/5A/5A_ETi_stack/modv0282.jpg]

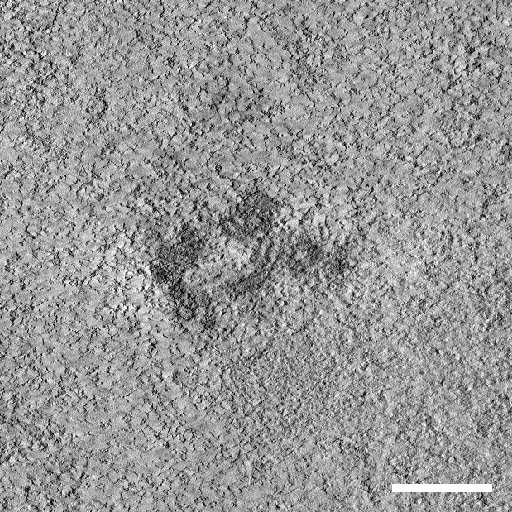

Supplement: Supplementary file 10 — Source data Fig. 5 [file 44318_2025_652_MOESM10_ESM.zip › Figure 5/5A/5A_ETi_stack/modv0296.jpg]

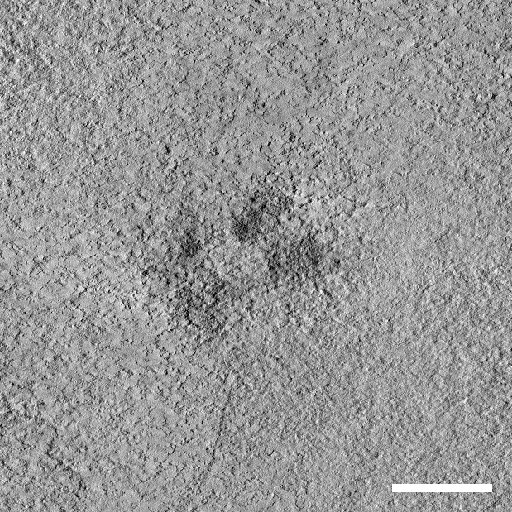

Supplement: Supplementary file 10 — Source data Fig. 5 [file 44318_2025_652_MOESM10_ESM.zip › Figure 5/5A/5A_ETi_stack/modv0335.jpg]

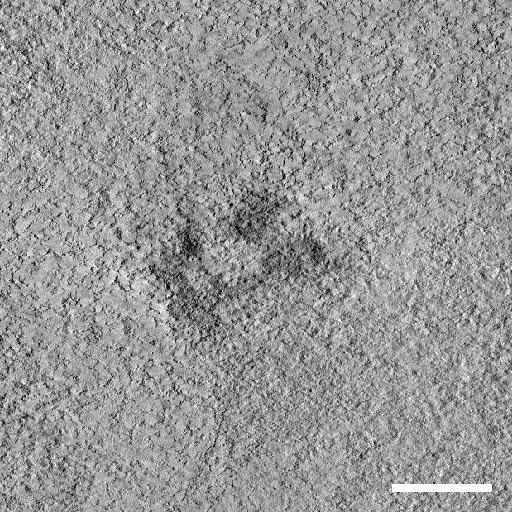

Supplement: Supplementary file 10 — Source data Fig. 5 [file 44318_2025_652_MOESM10_ESM.zip › Figure 5/5A/5A_ETi_stack/modv0321.jpg]

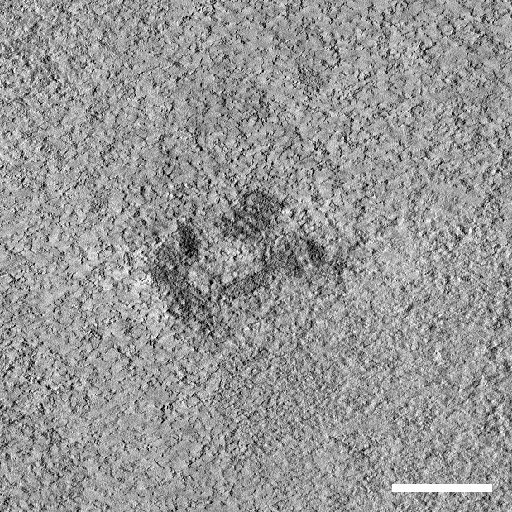

Supplement: Supplementary file 10 — Source data Fig. 5 [file 44318_2025_652_MOESM10_ESM.zip › Figure 5/5A/5A_ETi_stack/modv0309.jpg]

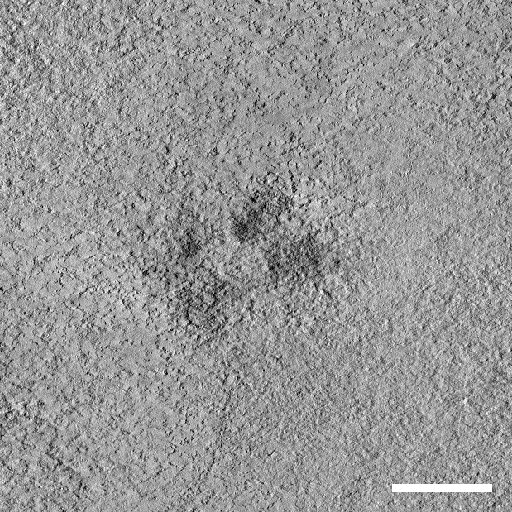

Supplement: Supplementary file 10 — Source data Fig. 5 [file 44318_2025_652_MOESM10_ESM.zip › Figure 5/5A/5A_ETi_stack/modv0337.jpg]

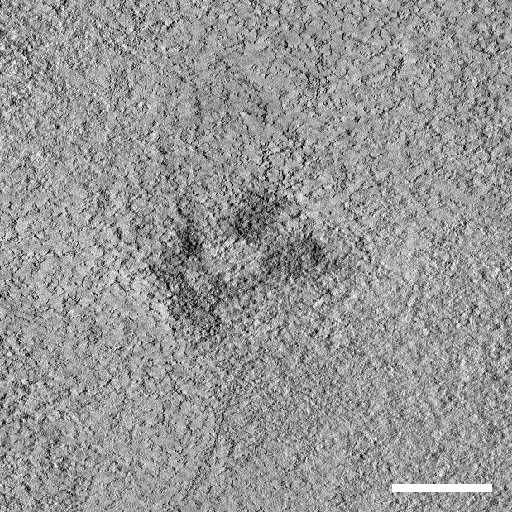

Supplement: Supplementary file 10 — Source data Fig. 5 [file 44318_2025_652_MOESM10_ESM.zip › Figure 5/5A/5A_ETi_stack/modv0323.jpg]

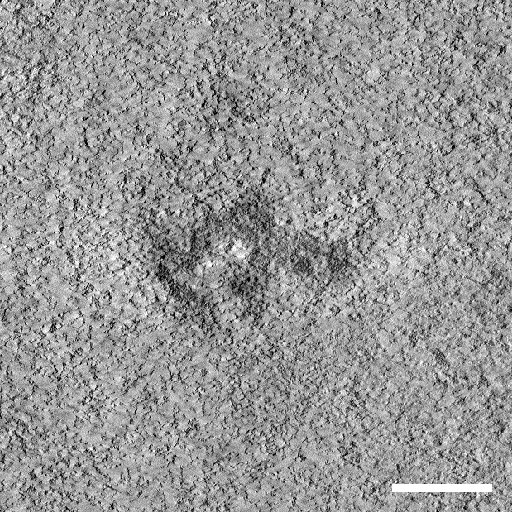

Supplement: Supplementary file 10 — Source data Fig. 5 [file 44318_2025_652_MOESM10_ESM.zip › Figure 5/5A/5A_ETi_stack/modv0280.jpg]

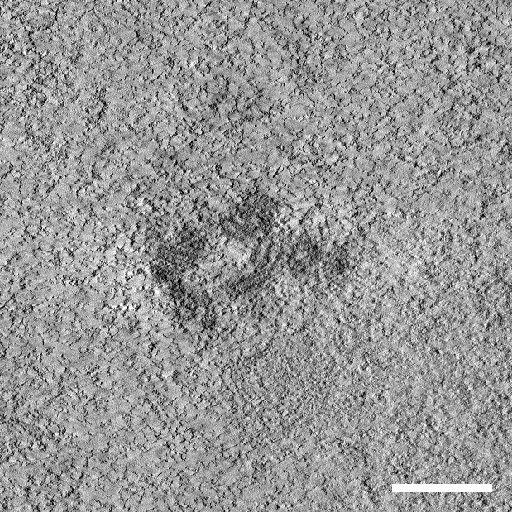

Supplement: Supplementary file 10 — Source data Fig. 5 [file 44318_2025_652_MOESM10_ESM.zip › Figure 5/5A/5A_ETi_stack/modv0294.jpg]

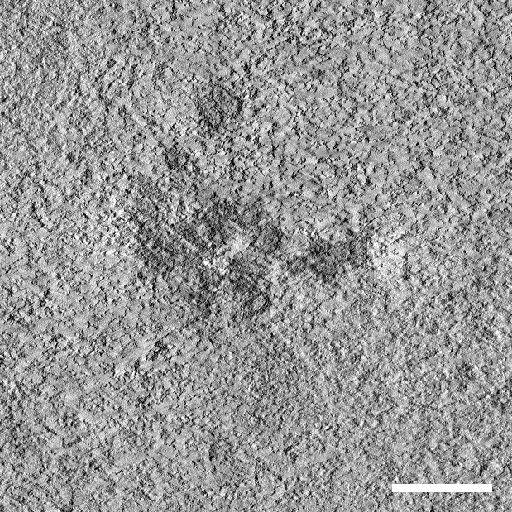

Supplement: Supplementary file 10 — Source data Fig. 5 [file 44318_2025_652_MOESM10_ESM.zip › Figure 5/5A/5A_ETi_stack/modv0243.jpg]

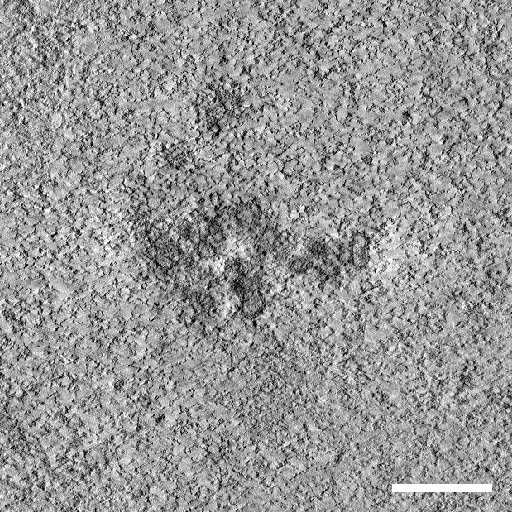

Supplement: Supplementary file 10 — Source data Fig. 5 [file 44318_2025_652_MOESM10_ESM.zip › Figure 5/5A/5A_ETi_stack/modv0257.jpg]

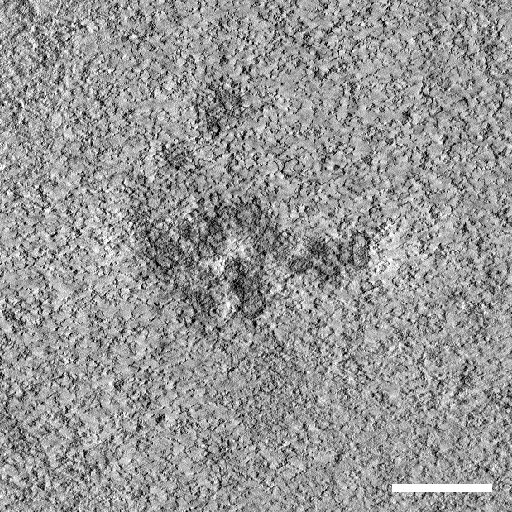

Supplement: Supplementary file 10 — Source data Fig. 5 [file 44318_2025_652_MOESM10_ESM.zip › Figure 5/5A/5A_ETi_stack/modv0256.jpg]

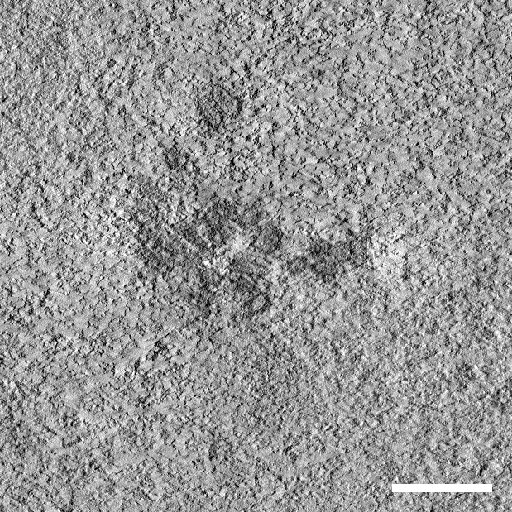

Supplement: Supplementary file 10 — Source data Fig. 5 [file 44318_2025_652_MOESM10_ESM.zip › Figure 5/5A/5A_ETi_stack/modv0242.jpg]

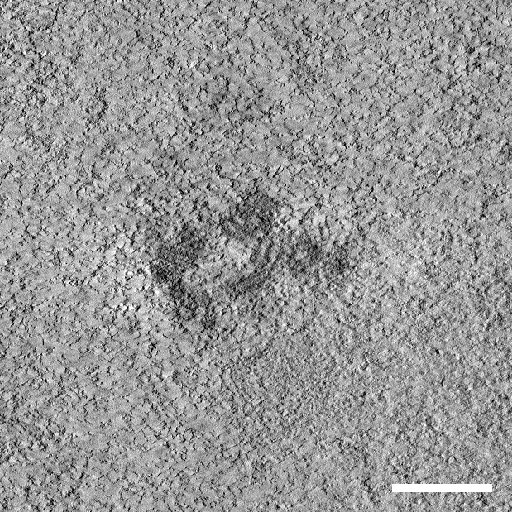

Supplement: Supplementary file 10 — Source data Fig. 5 [file 44318_2025_652_MOESM10_ESM.zip › Figure 5/5A/5A_ETi_stack/modv0295.jpg]
