# Supplementary material for: Lenacapavir-induced capsid damage uncovers HIV-1 genomes emanating from nuclear speckles
Source: EMBO J. 2025 Dec 1;45(2):449–70. doi: 10.1038/s44318-025-00652-5 (PMC12811339; doi:10.1038/s44318-025-00652-5)
Supplement: Supplementary file 11 — Expanded View Figures [file 44318_2025_652_MOESM11_ESM.pdf]

Expanded View Figures

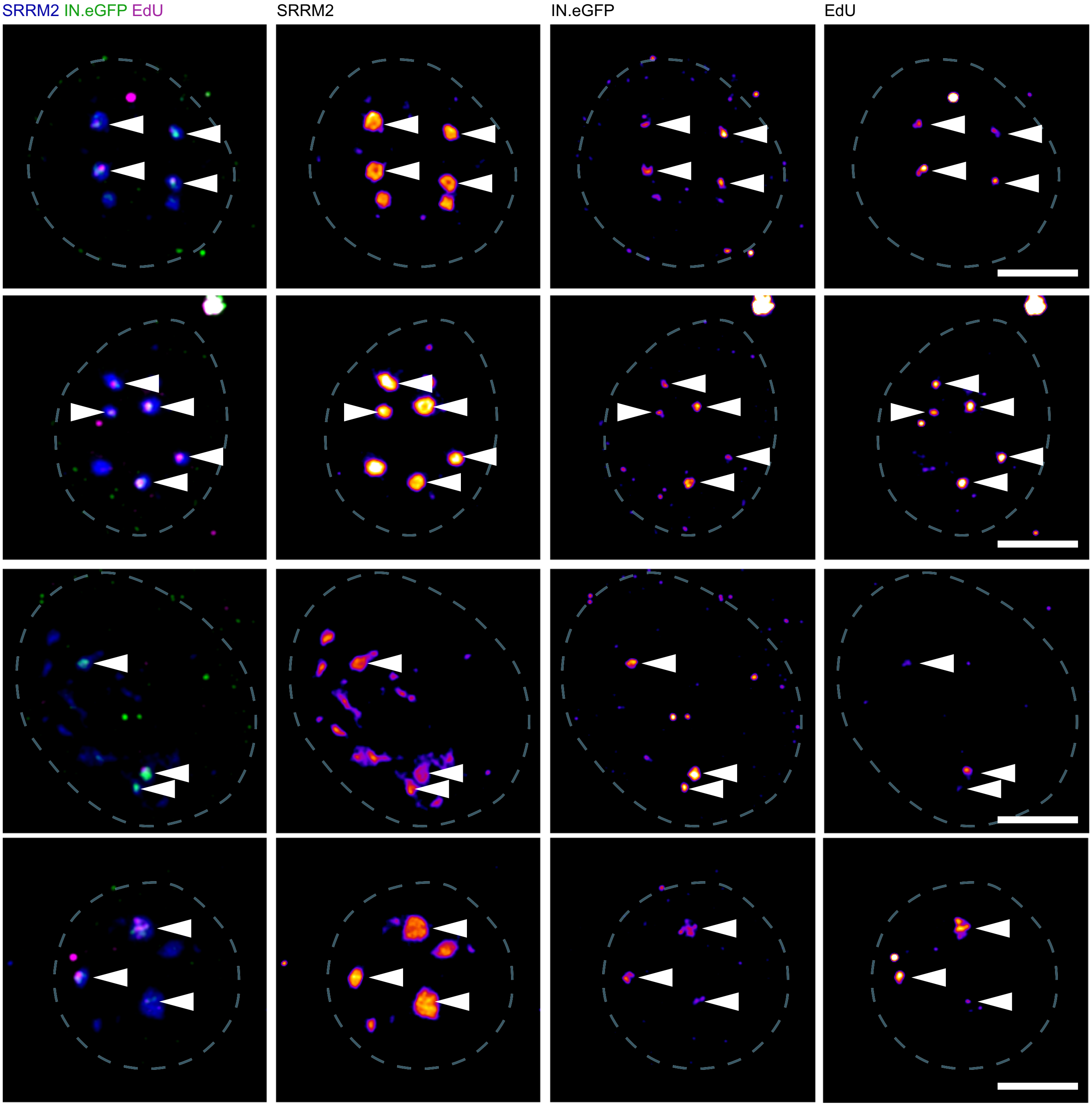

Figure EV1. Additional examples of subviral complexes in nuclear speckles of primary MDM.

Super-resolution analysis of HIV-1 cDNA within nuclear speckles of monocyte-derived macrophages (MDM) showing EdU (magenta) and IN.eGFP signals (green) in the center of SRRM2 condensates (blue). Shown are four additional maximum intensity projection of MDM nuclei (white dashed line) infected for 72 h with VSV-G pseudotyped NNHIV in presence of EdU followed by fixation, EdU click labeling and immunofluorescence staining using an antibody against SRRM2 (SC35). White arrowheads indicate a selection of nuclear IN.eGFP objects for clarity. Samples were imaged using Airyscan microscopy. Scale bars: 5 µm.

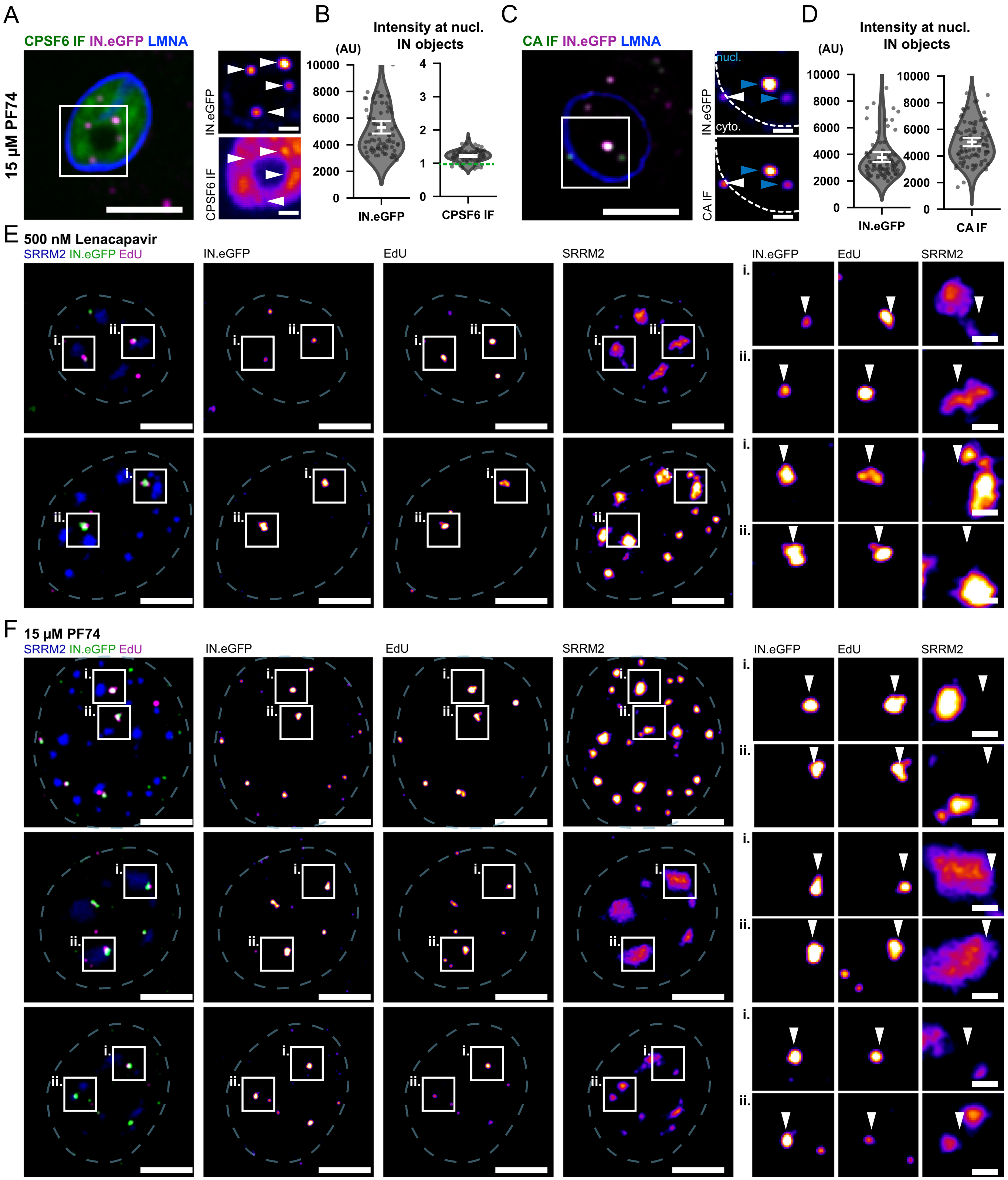
A
CPSF6 IF IN.eGFP LMNA
15 µM PF74
IN.eGFP
CPSF6 IF
B
Intensity at nucl. IN objects
(AU)
10000
8000
6000
4000
2000
0
4
3
2
1
0
IN.eGFP
CPSF6 IF
C
CA IF IN.eGFP LMNA
nucl.
cyto.
IN.eGFP
CA IF
D
Intensity at nucl. IN objects
(AU)
IN.eGFP
CA IF
E 500 nM Lenacapavir
SRRM2 IN.eGFP EdU
IN.eGFP
EdU
SRRM2
i.
ii.
F 15 µM PF74
SRRM2 IN.eGFP EdU
IN.eGFP
EdU
SRRM2

Figure EV2. Effect of PF74 on CA and CPSF6 signals in MDM and additional examples of LEN and PF74 induced subviral particle exit from nuclear speckles.

(**A–F**) MDM were infected using IN.eGFP labeled VSV-G pseudotyped NNHIV IN.SNAP for 72 h before addition of indicated concentrations of PF74 or LEN for 1 h. Cells were fixed and immunostained before 3D SDCM imaging (**A–D**) or 3D Airyscan imaging (**E**, **F**). Samples were stained for CPSF6 (**A**, **B**), CA (**C**, **D**) or SRRM2 (**E**, **F**). Maximum intensity projections are shown. Error bars represent SEM. Scale bars: 5 µm (overviews) and 1 µm (enlargements). (**A–D**) Stripping of pre-assembled CPSF6 (**A**, **B**) and exposure of masked CA epitopes (**C**, **D**) by 15 µm PF74. (**A**, **E**, **F**) White arrowheads indicate a selection of nuclear IN.eGFP objects for clarity. (**C**) White arrowheads indicate cytoplasmic IN.eGFP objects whereas blue arrowheads indicate nuclear IN.eGFP objects. Dotted lines indicate nuclear boundary. (**B**, **D**) Images were analyzed by automated quantification using custom-made Python code as described in the Methods and protocols section. CPSF6 signals (**B**) were normalized to the mean nuclear CPSF6 expression level of the respective cell (green dotted line at $y = 1$). (**E**, **F**) Displacement of IN.eGFP objects from nuclear speckles. Cells were infected in presence of 10 µm EdU and click labeled prior to immunofluorescence staining. Shown are two representative cell nuclei treated with 500 nM LEN for 1 h (**E**) and three nuclei treated with 15 µM PF74 for 1 h (**F**).

© The Author(s)

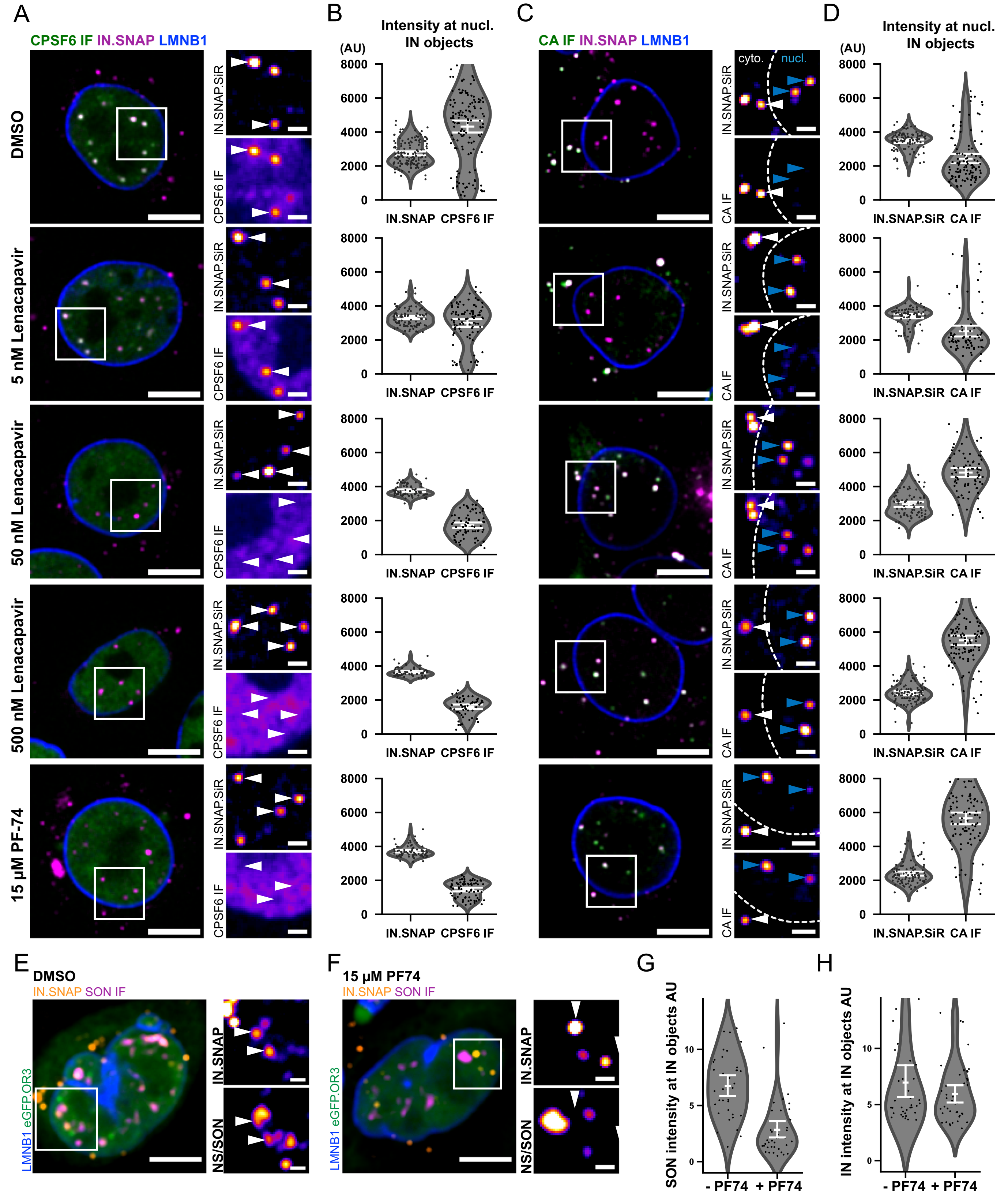
A
CPSF6 IF IN.SNAP LMNB1
DMSO
5 nM Lenacapavir
50 nM Lenacapavir
500 nM Lenacapavir
15 µM PF-74
IN.SNAP.SiR
CPSF6 IF
B
Intensity at nucl. IN objects
(AU)
IN.SNAP CPSF6 IF
C
CA IF IN.SNAP LMNB1
cyto.
nucl.
CA IF
D
Intensity at nucl. IN objects
(AU)
IN.SNAP.SiR CA IF
E
DMSO
IN.SNAP SON IF
LMNB1 eGFP.OR3
IN.SNAP
NS/SON
F
15 µM PF74
IN.SNAP SON IF
LMNB1 eGFP.OR3
G
SON intensity at IN objects AU
- PF74 + PF74
H
IN intensity at IN objects AU
- PF74 + PF74

 © The Author(s)

Figure EV3. Lenacapavir and PF74 strip CPSF6 from nuclear capsids, concomitantly expose masked CA epitopes, and lead to capsid exit from nuclear speckles in HeLa-based TZM-bl cells.

(**A–H**) Cells were infected using IN.SNAP labeled VSV-G pseudotyped NNHIV for 24 h before addition of indicated amounts of Lenacapavir, PF74 or DMSO for 1 h. Cells were fixed and immunostained before 3D confocal spinning disc imaging. Samples were stained for CPSF6 (**A**, **B**), CA (**C**, **D**) or SON (**E–H**). Shown is one of three independent experiments. Error bars represent SEM. Scale bars: 5 µm (overviews) and 1 µm (enlargements). (**A–D**) Stripping of pre-assembled CPSF6 (**A**, **B**) and concomitant exposure of masked CA epitopes (**C**, **D**) by Lenacapavir and PF74. (**A**, **E**, **F**) White arrowheads indicate a selection of nuclear IN.SNAP objects for clarity. (**C**) White arrowheads indicate cytoplasmic IN.SNAP objects whereas blue arrowheads indicate nuclear IN.SNAP objects. (**B**, **D**) Images were analyzed by automated quantification using custom-made Python code. Nuclear 3D IN.SNAP objects were segmented and mean intensities in the respective channels quantified. (**E–H**) Displacement of IN.SNAP objects from nuclear speckles. Shown are representative cells treated with DMSO (**E**) or 15 µM PF74 (**F**) for 1 h. (**G**, **H**) Quantification of SON (**G**) or IN.SNAP (**H**) mean intensities shown in (**E**, **F**) at nuclear 3D IN.SNAP objects in presence and absence of 15 µM PF74.

© The Author(s)

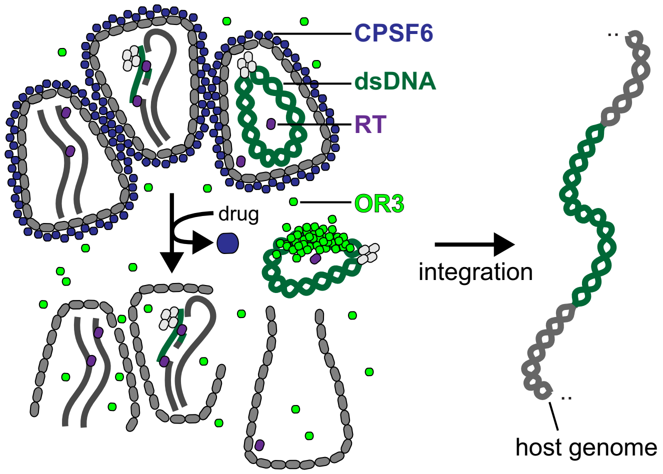

Figure EV4. Scheme of LEN/PF74 induced exposure of HIV-1 dsDNA.

LEN or PF74 displace CPSF6 from HIV-1 capsids clustered within nuclear speckles, thereby relocalizing these complexes to speckle-adjacent sites. Concomitantly, capsid structures are damaged, with bifurcated protrusions appearing at the narrow end of capsids. Functionally complete reverse-transcribed HIV-1 genomes are released and integrate into the host-cell chromatin.

 © The Author(s)
